# Supplementary material for: Association between physical measures of spinopelvic alignment and physical functioning with patient reported outcome measures after total hip arthroplasty: Systematic review and narrative synthesis
Source: PLoS One. 2025 Dec 29;20(12):e0339615. doi: 10.1371/journal.pone.0339615 (PMC12747333; doi:10.1371/journal.pone.0339615)
Supplement: S2 Appendix — (DOCX) [file pone.0339615.s002.docx]

**Search strategy in MEDLINE (Ovid)**

1 (hip adj4 (osteoarthriti* or arthriti* or arthros* or osteoarthrosi*)).tw,kf.

2 Hip Joint/ or Osteoarthritis, Hip/ or Arthroplasty, Replacement, Hip/ or Hip Prosthesis/ or Hip/

3 (hip joint prosthes* or hip prosthes* or THA).tw,kf.

4 (hip adj4 (remov* or surg* or replac* or arthroplast*)).tw,kf.

5 coxarthrosi*.tw,kf.

6 (hip implant* or artificial hip joint*).tw,kf.

7 or/1-6

8 (spin* align* or vertebra* align* or spin* column or vertebra* column or spinopelvic align* or lumbosacral align* or sagittal align*).tw,kf.

9 (spine-hip relation* or cervic* vertebra* or thoracic* align* or lumbar* align* or sacral* align* or hip* angle* or spinopelvic* or alignment*).tw,kf.

10 ((pelvis* or pelvic* or hip* or spin*) adj4 alignment).tw,kf.

11 cervical lordos*.tw,kf.

12 thoracic kyphos*.tw,kf.

13 lumbar lordos*.tw,kf.

14 lumbar scolios*.tw,kf.

15 T1 spinopelvic inclination*.tw,kf.

16 pelvic tilt*.tw,kf.

17 T1 pelvic*.tw,kf.

18 sacral slope*.tw,kf.

19 pelvic incidence*.tw,kf.

20 pelvic inclination*.tw,kf.

21 anterior pelvic plane*.tw,kf.

22 sagittal vertical axis*.tw,kf.

23 spinosacral*.tw,kf.

24 (cup inclination* or acetabular inclination*).tw,kf.

25 cup anteversion*.tw,kf.

26 acetabular anteversion*.tw,kf.

27 or/8-26

28 (physical outcome* or physical* measure* or physical* assess* or objective* outcome* or objective* assess* or objective* measure* or physical function* or function* measure*).tw,kf.

29 "Range of Motion, Articular"/

30 (range of motion or ROM).tw,kf.

31 Schober.tw,kf.

32 (finger* adj4 (floor or toe or knee or fibular head)).tw,kf.

33 (wrist crease adj3 floor).tw,kf.

34 (straight leg raise* or SLR or Lasegue*).tw,kf.

35 (isometric strength or isokinetic strength).tw,kf.

36 (motor control or movement control or functional movement screen* or FMS or sitting one leg knee extension or posterior pelvic tilt or waiter's bow or one leg stance).tw,kf.

37 gait/ or gait analysis/

38 walking speed/

39 (spatiotemporal gait or spatio temporal gait or stride length or stride duration or gait speed or cadence or gait asymmetry or stance phase or swing phase or double limb support or single limb support).tw,kf.

40 (inclinometer or goniometer or kyphometer or electromagnetic tracking).tw,kf.

41 muscle strength dynamometer/

42 (dynamometer or manual muscle test or MedX or Cybex or Kin-Com or RehaGait or JAMAR).tw,kf.

43 (aerobic capacity or VO2* or bicycle ergomet* or maximal graded exercise* or steep ramp).tw,kf.

44 (Biering Sorensen or modified Sorensen).tw,kf.

45 Roman chair.tw,kf.

46 (sternum adj3 (ground or floor)).tw,kf.

47 muscle endurance.tw,kf.

48 ((back or body or trunk) adj3 endurance).tw,kf.

49 prone bridge.tw,kf.

50 ((lower extremities or (hips and knees)) adj5 (90deg* or "90 degree*")).tw,kf.

51 (arch-up* or sit-up* or squat* or dumbbell press*).tw,kf.

52 (double limb stance or single limb stance or stork stand* or flamingo balance or y-balance).tw,kf.

53 CTSIB.tw,kf.

54 (clinical test* adj2 sensory interaction adj2 balance).tw,kf.

55 (clinical test* adj2 sensory integration adj2 balance).tw,kf.

56 (Berg balance scale or Tinetti* or performance oriented mobility assessment* or tandem walk*).tw,kf.

57 lower extremity motor coordination test*.tw,kf.

58 (chair adj3 (stand* or rise*)).tw,kf.

59 (sit to stand or stand up or stand ups or roll*).tw,kf.

60 (lie adj2 sit).tw,kf.

61 (bed adj2 chair).tw,kf.

62 step*.tw,kf.

63 ((stand or standing) adj2 continuous).tw,kf.

64 functional capacity evaluation.tw,kf.

65 lifting/

66 functional reach.tw,kf.

67 (lift* or progressive isoinertial lifting evaluation or pile).tw,kf.

68 forward reach.tw,kf.

69 walk test/

70 (self-paced walk* or 4-meter walk* or 4-metre walk* or 5-meter walk* or 5-metre walk* or 10-meter walk* or 10-metre walk* or 15-meter walk* or 15-metre walk* or 50-meter walk* or 50-metre walk* or 50-foot walk* or 5-minute walk* or 6-minute walk* or treadmill or overground walk*).tw,kf.

71 (walk adj3 hall*).tw,kf.

72 shuttle walk*.tw,kf.

73 (stair* adj2 climb*).tw,kf.

74 ("timed up and go" or TUG or "8 foot up and go").tw,kf.

75 (Physical capability assessment tool or PCAT or aggregated functional performance test or aggregated assessment of physical function or short physical performance battery or cumulated ambulation score or functional independence measure or Katz ADL index).tw,kf.

76 (Activity measure for post-acute care 6 clicks or Activity measure for postacute care 6 clicks).tw,kf.

77 (Physiotherapy functional mobility profile or Barthel index).tw,kf.

78 lying.tw,kf.

79 (time adj5 stand*).tw,kf.

80 (constant postures or active postures or sedentary postures or sedentary activity or walking time or walking distance or claudication index or walking speed or daily walking events or light intensity or moderate intensity or vigorous intensity or activity count or gait cycles or gait posture index or physical activity).tw,kf.

81 accelerometry/

82 wearable electronic devices/ or fitness trackers/

83 (acceleromet* or activity monitor or pedomet* or GPS or watch or smartwatch).tw,kf.

84 (6WT adj3 app*).tw,kf.

85 (muscle function or muscle length or muscle strength or muscle activity or EMG activity).tw,kf.

86 (muscle activ* or electromyography activ* or muscle stength test* or muscle strength grad* or muscular* assess*).tw,kf.

87 or/28-86

88 27 or 87

89 Pain Measurement/

90 Disability Evaluation/ or "International Classification of Functioning, Disability and Health"/

91 Patient Satisfaction/ or Motivation/ or Psychological/ or Personal Satisfaction/ or Self Efficacy/ or Self Care/

92 Interpersonal Relations/ or Social Behavior/ or Social Participation/ or Social Support/ or Social Isolation/

93 "Quality of Life"/ or Anxiety/ or Depression/ or Emotions/ or Stress, Psychological/ or Anger/ or Cognition/

94 Kinesiophobia/

95 "Surveys and Questionnaires"/

96 Patient expect*.tw,kf.

97 Visual Analogue Scale.tw,kf.

98 Physical functioning patient reported outcome*.tw,kf.

99 Treatment Outcome/ or Patient Reported Outcome Measures/ or Patient Outcome Assessment/

100 Back Pain/ or Low Back Pain/

101 (subjective* outcomes or subjectively measured outcome* or subjective assessment of outcome* or patient reported* or subjective* measure or PROM*).tw,kf.

102 ((health-related quality of life or HRQOL) adj2 questionnaire).tw,kf.

103 ((Roland-Morris Disability or RMD) adj2 questionnaire).tw,kf.

104 (Low* back* pain* or low* back* ache* or backache* or lumbar vertebra* pain or lumbar spin* pain or lumbosacral pain or lumbo-sacral pain or lumbar* pain or lumbago* or sciatica or radiculopath* or radicular pain*).tw,kf.

105 Oxford Hip Score.tw,kf.

106 harris hip score.tw,kf.

107 EQ-5D.tw,kf.

108 SF-36*.tw,kf.

109 ((hip disability and osteoarthritis outcome*) or HOOS*).tw,kf.

110 ((Western Ontario and McMaster Universities Arthritis Index) or WOMAC).tw,kf.

111 (Numeric* Rating Scale or NRS).tw,kf.

112 McGill Pain Questionnaire.tw,kf.

113 Tampa Scale of Kinesiophobia.tw,kf.

114 Beck Depression Inventory.tw,kf.

115 Nottingham Health Profile.tw,kf.

116 fear-avoidance beliefs questionnaire.tw,kf.

117 UCLA loneliness scale.tw,kf.

118 (Japanese Orthopaedic Association Hip-Disease Evaluation Questionnaire or JHEQ).tw,kf.

119 HOOS JR.tw,kf.

120 (Forgotten Joint Score-12 or FJS-12).tw,kf.

121 (short questionnaire to assess health-enhancing physical activity or SQUASH*).tw,kf.

122 (The Veterans RAND 12-Item Health Survey or VR-12*).tw,kf.

123 Likert scale.tw,kf.

124 ((The Copenhagen Hip and Groin Outcome Score) or HAGOS*).tw,kf.

125 ((The pain and function of the hip) or PFH*).tw,kf.

126 (the postoperative recovery profile or PRP*).tw,kf.

127 The Pain Disability Questionnaire.tw,kf.

128 Chronic Pain Self Efficacy Scale.tw,kf.

129 Pain Self-Efficacy Questionnaire.tw,kf.

130 The Pain Disability Index.tw,kf.

131 (Questionnaire for Physical Activity Decline in Pain or PAD).tw,kf.

132 Daily Activity Diary for Chronic Pain Patients.tw,kf.

133 the Multidimensional Pain Inventory.tw,kf.

134 the Brief Pain Inventory.tw,kf.

135 ((The Impact on Participation and Autonomy) or IPA).tw,kf.

136 ((The Impact on Participation and Autonomy Questionnaire) or IPAQ).tw,kf.

137 The Physical Activity Questionnaire.tw,kf.

138 (The Quality of Well-Being Scale or QWB).tw,kf.

139 (The Sickness Impact Profile or SIP).tw,kf.

140 (Work Limitations Questionnaire or WLQ).tw,kf.

141 Human Activity Profile.tw,kf.

142 Motor Fitness Scale.tw,kf.

143 PROMIS.tw,kf.

144 (Population Surveys of Chronic Disease and Disability).tw,kf.

145 the CDC HRQOL-14.tw,kf.

146 The Duke-UNC Health Profile.tw,kf.

147 Oswestry Disability Index.tw,kf.

148 Health Assessment Questionnaire.tw,kf.

149 Functional Status Questionnaire.tw,kf.

150 Rosow Breslau Index of Mobility.tw,kf.

151 Short Musculoskeletal Function Assessment Questionnaire.tw,kf.

152 Musculoskeletal Functional Limitation Index.tw,kf.

153 Patient-specific activity scoring scheme.tw,kf.

154 or/89-153

155 (observational adj5 (study or studies or design or analysis or analyses)).ti,ab,kf.

156 (prospective adj5 (study or studies or design or analysis or analyses)).ti,ab,kf.

157 (retrospective adj6 (study or studies or design or analysis or analyses or data)).ti,ab,kf.

158 ((longitudinal or longterm or long term) adj6 (study or studies or design or analysis or analyses or data)).ti,ab,kf.

159 (cross sectional adj8 (study or studies or design or research or analysis or analyses or survey or findings)).ti,ab,kf.

160 (associat* or regression* or correlat* or relat*).tw,kf.

161 cohort*.ti,ab,kf.

162 Cohort Studies/

163 Observational Study/

164 Cross-Sectional Studies/

165 Prospective Studies/

166 Retrospective Studies/

167 Longitudinal Studies/

168 or/155-167

169 7 and 88 and 154 and 168

**Search strategy in EMBASE (Ovid)**

1 (hip adj4 (osteoarthriti* or arthriti* or arthros* or osteoarthrosi*)).tw,kf.

2 (hip joint prosthes* or hip prosthes* or THA).tw,kf.

3 (hip adj4 (remov* or surg* or replac* or arthroplast*)).tw,kf.

4 coxarthrosi*.tw,kf.

5 (hip implant* or artificial hip joint*).tw,kf.

6 total hip replacement/ or total hip prosthesis/ or hip prosthesis/ or hip arthroplasty/ or hip surgery/ or hip osteoarthritis/ or coxitis/

7 1 or 2 or 3 or 4 or 5 or 6

8 (spin* align* or vertebra* align* or spin* column or vertebra* column or spinopelvic align* or lumbosacral align* or sagittal align*).tw,kf.

9 (spine-hip relation* or cervic* vertebra* or thoracic* align* or lumbar* align* or sacral* align* or hip* angle* or spinopelvic* or alignment*).tw,kf.

10 ((pelvis* or pelvic* or hip* or spin*) adj4 alignment).tw,kf.

11 cervical lordos*.tw,kf.

12 thoracic kyphos*.tw,kf.

13 lumbar lordos*.tw,kf.

14 lumbar scolios*.tw,kf.

15 T1 spinopelvic inclination*.tw,kf.

16 pelvic tilt*.tw,kf.

17 T1 pelvic*.tw,kf.

18 sacral slope*.tw,kf.

19 pelvic incidence*.tw,kf.

20 pelvic inclination*.tw,kf.

21 anterior pelvic plane*.tw,kf.

22 sagittal vertical axis*.tw,kf.

23 spinosacral*.tw,kf.

24 (cup inclination* or acetabular inclination*).tw,kf.

25 cup anteversion*.tw,kf.

26 acetabular anteversion*.tw,kf.

27 lumbar lordosis angle/ or lordosis/ or cervical lordosis angle/ or thoracic kyphosis angle/ or kyphosis/ or scoliosis/ or pelvic tilt/ or pelvic inclination/ or pelvic incidence/ or acetabular inclination/ or acetabular anteversion/

28 8 or 9 or 10 or 11 or 12 or 13 or 14 or 15 or 16 or 17 or 18 or 19 or 20 or 21 or 22 or 23 or 24 or 25 or 27

29 (physical outcome* or physical* measure* or physical* assess* or objective* outcome* or objective* assess* or objective* measure* or physical function* or function* measure*).tw,kf.

30 "range of motion"/

31 (range of motion or ROM).tw,kf.

32 Schober.tw,kf.

33 (finger* adj4 (floor or toe or knee or fibular head)).tw,kf.

34 (wrist crease adj3 floor).tw,kf.

35 (straight leg raise* or SLR or Lasegue*).tw,kf.

36 (isometric strength or isokinetic strength).tw,kf.

37 (motor control or movement control or functional movement screen* or FMS or sitting one leg knee extension or posterior pelvic tilt or waiter's bow or one leg stance).tw,kf.

38 gait/

39 walking speed/

40 (spatiotemporal gait or spatio temporal gait or stride length or stride duration or gait speed or cadence or gait asymmetry or stance phase or swing phase or double limb support or single limb support).tw,kf.

41 (inclinometer or goniometer or kyphometer or electromagnetic tracking).tw,kf. 5969

42 dynamometer/

43 (dynamometer or manual muscle test or MedX or Cybex or Kin-Com or RehaGait or JAMAR).tw,kf.

44 (aerobic capacity or VO2* or bicycle ergomet* or maximal graded exercise* or steep ramp).tw,kf.

45 (Biering Sorensen or modified Sorensen).tw,kf.

46 Roman chair.tw,kf.

47 (sternum adj3 (ground or floor)).tw,kf.

48 muscle endurance.tw,kf.

49 ((back or body or trunk) adj3 endurance).tw,kf.

50 prone bridge.tw,kf.

51 ((lower extremities or (hips and knees)) adj5 (90deg* or "90 degree*")).tw,kf.

52 (arch-up* or sit-up* or squat* or dumbbell press*).tw,kf.

53 (double limb stance or single limb stance or stork stand* or flamingo balance or y-balance).tw,kf.

54 CTSIB.tw,kf.

55 (clinical test* adj2 sensory interaction adj2 balance).tw,kf.

56 (clinical test* adj2 sensory integration adj2 balance).tw,kf.

57 (Berg balance scale or Tinetti* or performance oriented mobility assessment* or tandem walk*).tw,kf.

58 lower extremity motor coordination test*.tw,kf.

59 (chair adj3 (stand* or rise*)).tw,kf.

60 (sit to stand or stand up or stand ups or roll*).tw,kf.

61 (lie adj2 sit).tw,kf.

62 (bed adj2 chair).tw,kf.

63 step*.tw,kf.

64 ((stand or standing) adj2 continuous).tw,kf.

65 functional capacity evaluation.tw,kf.

66 biomechanics/

67 functional reach.tw,kf.

68 (lift* or progressive isoinertial lifting evaluation or pile).tw,kf.

69 forward reach.tw,kf.

70 walk test/

71 (self-paced walk* or 4-meter walk* or 4-metre walk* or 5-meter walk* or 5-metre walk* or 10-meter walk* or 10-metre walk* or 15-meter walk* or 15-metre walk* or 50-meter walk* or 50-metre walk* or 50-foot walk* or 5-minute walk* or 6-minute walk* or treadmill or overground walk*).tw,kf.

72 (walk adj3 hall*).tw,kf.

73 shuttle walk*.tw,kf.

74 (stair* adj2 climb*).tw,kf.

75 ("timed up and go" or TUG or "8 foot up and go").tw,kf.

76 (Physical capability assessment tool or PCAT or aggregated functional performance test or aggregated assessment of physical function or short physical performance battery or cumulated ambulation score or functional independence measure or Katz ADL index).tw,kf.

77 (Activity measure for post-acute care 6 clicks or Activity measure for postacute care 6 clicks).tw,kf.

78 (Physiotherapy functional mobility profile or Barthel index).tw,kf.

79 lying.tw,kf.

80 (time adj5 stand*).tw,kf.

81 (constant postures or active postures or sedentary postures or sedentary activity or walking time or walking distance or claudication index or walking speed or daily walking events or light intensity or moderate intensity or vigorous intensity or activity count or gait cycles or gait posture index or physical activity).tw,kf.

82 accelerometry/

83 wearable computer/

84 activity tracker/

85 (acceleromet* or activity monitor or pedomet* or GPS or watch or smartwatch).tw,kf.

86 (6WT adj3 app*).tw,kf.

87 (muscle function or muscle length or muscle strength or muscle activity or EMG activity).tw,kf.

88 (muscle activ* or electromyography activ* or muscle stength test* or muscle strength grad* or muscular* assess*).tw,kf.

89 muscle strength/ or muscle length/ or muscle contraction/ or muscle function/ or electromyography/

90 29 or 30 or 31 or 32 or 33 or 34 or 35 or 36 or 37 or 38 or 39 or 40 or 41 or 42 or 43 or 44 or 45 or 46 or 47 or 48 or 49 or 50 or 51 or 52 or 53 or 54 or 55 or 56 or 57 or 58 or 59 or 60 or 61 or 62 or 63 or 64 or 65 or 66 or 67 or 68 or 69 or 70 or 71 or 72 or 73 or 74 or 75 or 76 or 77 or 78 or 79 or 80 or 81 or 82 or 83 or 84 or 85 or 86 or 87 or 88 or 89

91 28 or 90

92 pain assessment/ or pain measurement/

93 disability assessment/

94 fear/ or Tampa scale for kinesiophobia/ or phobia/

95 patient satisfaction/ or satisfaction/ or self concept/ or self care/

96 motivation/

97 "quality of life"/ or social behavior/ or social participation/ or social interaction/ or social support/ or social isolation/

98 low back pain/

99 Patient expect*.tw,kf.

100 Visual Analogue Scale.tw,kf.

101 Physical functioning patient reported outcome*.tw,kf.

102 treatment outcome/ or patient-reported outcome/

103 anxiety/ or depression/ or mental stress/ or anger/ or cognition/

104 outcome assessment/

105 (subjective* outcomes or subjectively measured outcome* or subjective assessment of outcome* or patient reported* or subjective* measure or PROM*).tw,kf.

106 ((health-related quality of life or HRQOL) adj2 questionnaire).tw,kf.

107 ((Roland-Morris Disability or RMD) adj2 questionnaire).tw,kf.

108 (Low* back* pain* or low* back* ache* or backache* or lumbar vertebra* pain or lumbar spin* pain or lumbosacral pain or lumbo-sacral pain or lumbar* pain or lumbago* or sciatica or radiculopath* or radicular pain*).tw,kf.

109 Oxford Hip Score.tw,kf.

110 harris hip score.tw,kf.

111 EQ-5D.tw,kf.

112 SF-36*.tw,kf.

113 ((hip disability and osteoarthritis outcome*) or HOOS*).tw,kf.

114 ((Western Ontario and McMaster Universities Arthritis Index) or WOMAC).tw,kf.

115 "Western Ontario and McMaster Universities Osteoarthritis Index"/

116 (Numeric* Rating Scale or NRS).tw,kf.

117 UCLA loneliness scale.tw,kf.

118 (Japanese Orthopaedic Association Hip-Disease Evaluation Questionnaire or JHEQ).tw,kf.

119 HOOS JR.tw,kf.

120 "Hip Disability and Osteoarthritis Outcome Score"/

121 (Forgotten Joint Score-12 or FJS-12).tw,kf.

122 (short questionnaire to assess health-enhancing physical activity or SQUASH*).tw,kf.

123 (The Veterans RAND 12-Item Health Survey or VR-12*).tw,kf.

124 Likert scale.tw,kf.

125 ((The Copenhagen Hip and Groin Outcome Score) or HAGOS*).tw,kf.

126 ((The pain and function of the hip) or PFH*).tw,kf.

127 (the postoperative recovery profile or PRP*).tw,kf.

128 The Pain Disability Questionnaire.tw,kf.

129 Chronic Pain Self Efficacy Scale.tw,kf.

130 Pain Self-Efficacy Questionnaire.tw,kf.

131 (Questionnaire for Physical Activity Decline in Pain or PAD).tw,kf.

132 Daily Activity Diary for Chronic Pain Patients.tw,kf.

133 The Pain Disability Index.tw,kf.

134 the Multidimensional Pain Inventory.tw,kf.

135 the Brief Pain Inventory.tw,kf.

136 questionnaire/

137 McGill Pain Questionnaire.tw,kf.

138 Tampa Scale of Kinesiophobia.tw,kf.

139 Beck Depression Inventory.tw,kf.

140 Nottingham Health Profile.tw,kf.

141 Fear-avoidance beliefs questionnaire.tw,kf.

142 ((The Impact on Participation and Autonomy) or IPA).tw,kf.

143 ((The Impact on Participation and Autonomy Questionnaire) or IPAQ).tw,kf.

144 The Physical Activity Questionnaire.tw,kf.

145 (The Quality of Well-Being Scale or QWB).tw,kf.

146 (The Sickness Impact Profile or SIP).tw,kf.

147 (Work Limitations Questionnaire or WLQ).tw,kf.

148 Human Activity Profile.tw,kf.

149 Motor Fitness Scale.tw,kf.

150 PROMIS.tw,kf.

151 (Population Surveys of Chronic Disease and Disability).tw,kf.

152 the CDC HRQOL-14.tw,kf.

153 The Duke-UNC Health Profile.tw,kf.

154 Oswestry Disability Index.tw,kf.

155 Health Assessment Questionnaire.tw,kf.

156 Functional Status Questionnaire.tw,kf.

157 Rosow Breslau Index of Mobility.tw,kf.

158 Short Musculoskeletal Function Assessment Questionnaire.tw,kf.

159 Musculoskeletal Functional Limitation Index.tw,kf.

160 Patient-specific activity scoring scheme.tw,kf.

161 92 or 93 or 94 or 95 or 96 or 97 or 98 or 99 or 100 or 101 or 102 or 103 or 104 or 105 or 106 or 107 or 108 or 109 or 110 or 111 or 112 or 113 or 114 or 115 or 116 or 117 or 118 or 119 or 120 or 121 or 122 or 123 or 124 or 125 or 126 or 127 or 128 or 129 or 130 or 131 or 132 or 133 or 134 or 135 or 136 or 137 or 138 or 139 or 140 or 141 or 142 or 143 or 144 or 145 or 146 or 147 or 148 or 149 or 150 or 151 or 152 or 153 or 154 or 155 or 156 or 157 or 158 or 159 or 160

162 cohort analysis/

163 cross-sectional study/

164 observational study/

165 prospective study/

166 retrospective study/

167 longitudinal study/

168 (observational adj5 (study or studies or design or analysis or analyses)).ti,ab,kf.

169 (prospective adj5 (study or studies or design or analysis or analyses)).ti,ab,kf.

170 (retrospective adj6 (study or studies or design or analysis or analyses or data)).ti,ab,kf.

171 ((longitudinal or longterm or long term) adj6 (study or studies or design or analysis or analyses or data)).ti,ab,kf.

172 (cross sectional adj8 (study or studies or design or research or analysis or analyses or survey or findings)).ti,ab,kf.

173 (associat* or regression* or correlat* or relat*).tw,kf.

174 cohort*.ti,ab,kf.

175 162 or 163 or 164 or 165 or 166 or 167 or 168 or 169 or 170 or 171 or 172 or 173 or 174

176 7 and 91 and 161 and 175

**Search strategy in CINAHL**

S1 (hip N4 (osteoarthriti* or arthriti* or arthros* or osteoarthrosi*))

S2 (MH "Hip Joint") OR (MH "Arthroplasty, Replacement, Hip") OR (MH "Osteoarthritis, Hip") OR (MH "Hip") OR (MH "Hip Surgery")

S3 (hip joint prosthes* or hip prosthes* or total hip arthroplasty)

S4 (hip N4 (remov* or surg* or replac* or arthroplast*))

S5 coxarthrosi*

S6 (hip implant* or artificial hip joint*)

S7 (S1 OR S2 OR S3 OR S4 OR S5 OR S6)

S8 (spin* align* or vertebra* align* or spin* column or vertebra* column or spinopelvic align* or lumbosacral align* or sagittal align*)

S9 (spine-hip relation* or cervic* vertebra* or thoracic* align* or lumbar* align* or sacral* align* or hip* angle* or spinopelvic* or alignment*)

S10 ((pelvis* or pelvic* or hip* or spin*) N4 alignment)

S11 cervical lordos*

S12 thoracic kyphos*

S13 lumbar lordos*

S14 lumbar scolios*

S15 T1 spinopelvic inclination*

S16 pelvic tilt*

S17 T1 pelvic*

S18 sacral slope*

S19 pelvic incidence*

S20 pelvic inclination*

S21 anterior pelvic plane*

S22 sagittal vertical axis*

S23 spinosacral*

S24 (cup inclination* or acetabular inclination*)

S25 cup anteversion*

S26 acetabular anteversion*

S27 (S8 OR S9 OR S10 OR S11 OR S12 OR S13 OR S14 OR S15 OR S16 OR S17 OR S18 OR S19 OR S20 OR S21 OR S22 OR S23 OR S24 OR S25 OR S26)

S28 (physical outcome* or physical* measure* or physical* assess* or objective* outcome* or objective* assess* or objective* measure* or physical function* or function* measure*)

S29 (MH "Range of Motion") OR (MH "Motion")

S30 Schober*

S31 (finger* N4 (floor or toe or knee or fibular head))

S32 (wrist crease N3 floor)

S33 (straight leg raise* or SLR or Lasegue*)

S34 isometric strength*

S35 (motor control or movement control or functional movement screen* or FMS or sitting one leg knee extension or posterior pelvic tilt or waiter's bow or one leg stance)

S36 (MH "Gait") OR (MH "Gait Analysis") OR (MH "Walking Speed")

S37 (spatiotemporal gait or spatio temporal gait or stride length or stride duration or gait speed or cadence or gait asymmetry or stance phase or swing phase or double limb support or single limb support)

S38 (inclinometer or goniometer or kyphometer or electromagnetic tracking)

S39 (MH "Dynamometry")

S40 (dynamometer or manual muscle test or MedX or Cybex or Kin-Com or RehaGait or JAMAR)

S41 (aerobic capacity or VO2* or bicycle ergomet* or maximal graded exercise* or steep ramp)

S42 (Biering Sorensen* or modified Sorensen*)

S43 Roman chair*

S44 (sternum N3 (ground or floor))

S45 muscle endurance*

S46 ((back or body or trunk) N3 endurance)

S47 prone bridge*

S48 ((lower extremities or (hips and knees)) N5 (90deg* or "90 degree*"))

S49 (arch-up* or sit-up* or squat* or dumbbell press*)

S50 (double limb stance or single limb stance or stork stand* or flamingo balance or y-balance)

S51 isokinetic strength*

S52 tandem walk*

S53 CTSIB*

S54 (clinical test* N2 sensory interaction N2 balance)

S55 (clinical test* N2 sensory integration N2 balance)

S56 (Berg balance scale or Tinetti* or performance oriented mobility assessment*)

S57 lower extremity motor coordination test*

S58 (chair N3 (stand* or rise*))

S59 (sit to stand or stand up or stand ups or roll*)

S60 (lie N2 sit)

S61 (bed N2 chair)

S62 step*

S63 ((stand or standing) N2 continuous)

S64 functional capacity evaluation*

S65 (MH "Lifting")

S66 functional reach*

S67 (lift* or progressive isoinertial lifting evaluation or pile)

S68 forward reach*

S69 walk test*

S70 (self-paced walk* or 4-meter walk* or 4-metre walk* or 5-meter walk* or 5-metre walk* or 10-meter walk* or 10-metre walk* or 15-meter walk* or 15-metre walk* or 50-meter walk* or 50-metre walk* or 50-foot walk* or 5-minute walk* or 6-minute walk* or treadmill or overground walk*)

S71 (walk N3 hall*)

S72 shuttle walk*

S73 (stair* N2 climb*)

S74 ("timed up and go" or TUG or "8 foot up and go")

S75 (Physical capability assessment tool or PCAT or aggregated functional performance test or aggregated assessment of physical function or short physical performance battery or cumulated ambulation score or functional independence measure or Katz ADL index)

S76 (Activity measure for post-acute care 6 clicks or Activity measure for postacute care 6 clicks)

S77 (Physiotherapy functional mobility profile or Barthel index)

S78 lying*

S79 (time N5 stand*)

S80 (constant postures or active postures or sedentary postures or sedentary activity or walking time or walking distance or claudication index or walking speed or daily walking events or light intensity or moderate intensity or vigorous intensity or activity count or gait cycles or gait posture index or physical activity)

S81 (MH "Accelerometry")

S82 wearable electronic device

S83 (MH "Fitness Trackers")

S84 (acceleromet* or activity monitor or pedomet* or GPS or watch or smartwatch)

S85 (6WT N3 app*).

S86 (muscle function* or muscle length* or muscle strength* or muscle activ* or EMG activ*)

S87 (electromyography activ* or muscular* assess*)

S88 (S28 OR S29 OR S30 OR S31 OR S32 OR S33 OR S34 OR S35 OR S36 OR S37 OR S38 OR S39 OR S40 OR S41 OR S42 OR S43 OR S44 OR S45 OR S46 OR S47 OR S48 OR S49 OR S50 OR S51 OR S52 OR S53 OR S54 OR S55 OR S56 OR S57 OR S58 OR S59 OR S60 OR S61 OR S62 OR S63 OR S64 OR S65 OR S66 OR S67 OR S68 OR S69 OR S70 OR S71 OR S72 OR S73 OR S74 OR S75 OR S76 OR S77 OR S78 OR S79 OR S80 OR S81 OR S82 OR S83 OR S84 OR S85 OR S86 OR S87)

S89 (S27 OR S88)

S90 (MM "Pain Measurement") OR (MM "Pelvic Pain") OR (MM "Outcome Assessment") OR (MM "Back Pain") OR (MM "Low Back Pain") OR (MM "Pain") OR (MH "Musculoskeletal Pain") OR (MM "Brief Pain Inventory")

S91 (MM "Disability Evaluation")

S92 (MH "Patient Satisfaction") OR (MH "Personal Satisfaction") OR (MM "Self-Efficacy") OR (MM "Self Care")

S93 (MH "Motivation")

S94 (MH "Quality of Life") OR (MH "Social Participation") OR (MH "Social Behavior") OR (MH "Social Isolation") OR (MH "Social Support Index")

S95 (MH "Anxiety") OR (MH "Depression") OR (MH "Beck Depression Inventory, Revised Edition") OR (MH "Stress") OR (MH "Anger") OR (MH "Cognition") OR (MH "Kinesiophobia")

S96 (MH "Visual Analog Scaling")

S97 Physical functioning patient reported outcome*

S98 (MH "Treatment Outcomes") OR (MH "Outcome Assessment") OR (MH "Patient-Reported Outcomes")

S99 (MH "Patient Assessment") OR (MH "Outcomes (Health Care)")

S100 (subjective* outcomes or subjectively measured outcome* or subjective assessment of outcome* or patient reported* or subjective* measure or PROM*)

S101 ((health-related quality of life or HRQOL) N2 questionnaire)

S102 ((Roland-Morris Disability or RMD) N2 questionnaire)

S103 Low* back* pain* or low* back* ache* or backache* or lumbar vertebra* pain or lumbar spin* pain or lumbosacral pain or lumbo-sacral pain or lumbar* pain or lumbago* or sciatica or radiculopath* or radicular pain*

S104 Oxford Hip Score

S105 harris hip score

S106 (MH "Short Form-36 Health Survey (SF-36)")

S107 ((hip disability and osteoarthritis outcome*) or HOOS*)

S108 ((Western Ontario and McMaster Universities Arthritis Index) or WOMAC)

S109 (Numeric* Rating Scale or NRS)

S110 UCLA loneliness scale

S111 (Japanese Orthopaedic Association Hip-Disease Evaluation Questionnaire or JHEQ)

S112 HOOS JR

S113 (Forgotten Joint Score-12 or FJS-12)

S114 (short questionnaire to assess health-enhancing physical activity or SQUASH*)

S115 (The Veterans RAND 12-Item Health Survey or VR-12*)

S116 Likert scale

S117 ((The Copenhagen Hip and Groin Outcome Score) or HAGOS*)

S118 ((The pain and function of the hip) or PFH*)

S119 (the postoperative recovery profile or PRP*)

S120 The Pain Disability Questionnaire

S121 Chronic Pain Self Efficacy Scale.

S122 Pain Self-Efficacy Questionnaire

S123 (Questionnaire for Physical Activity Decline in Pain or PAD)

S124 Daily Activity Diary for Chronic Pain Patients

S125 The Pain Disability Index

S126 the Multidimensional Pain Inventory

S127 Patient expect*

S128 McGill Pain Questionnaire

S129 ((The Impact on Participation and Autonomy) or IPA)

S130 ((The Impact on Participation and Autonomy Questionnaire) or IPAQ)

S131 The Physical Activity Questionnaire

S132 (The Quality of Well-Being Scale or QWB)

S133 (The Sickness Impact Profile or SIP)

S134 (Work Limitations Questionnaire or WLQ)

S135 Human Activity Profile

S136 Motor Fitness Scale

S137 PROMIS

S138 (Population Surveys of Chronic Disease and Disability)

S139 the CDC HRQOL-14

S140 The Duke-UNC Health Profile

S141 Oswestry Disability*

S142 Rosow Breslau Index*

S143 Health Assessment Questionnaire

S144 fear-avoidance beliefs questionnaire

S145 Functional Status Questionnaire

S146 Tampa Scale of Kinesiophobia

S147 Beck Depression Inventory

S148 Nottingham Health Profile

S149 Short Musculoskeletal Function Assessment Questionnaire

S150 Health Assessment Questionnaire

S151 Musculoskeletal Functional Limitation Index

S152 Patient-specific activity scor*

S153 (S90 OR S91 OR S92 OR S93 OR S94 OR S95 OR S96 OR S97 OR S98 OR S99 OR S100 OR S101 OR S102 OR S103 OR S104 OR S105 OR S106 OR S107 OR S108 OR S109 OR S110 OR S111 OR S112 OR S113 OR S114 OR S115 OR S116 OR S117 OR S118 OR S119 OR S120 OR S121 OR S122 OR S123 OR S124 OR S125 OR S126 OR S127 OR S128 OR S129 OR S130 OR S131 OR S132 OR S133 OR S134 OR S135 OR S136 OR S137 OR S138 OR S139 OR S140 OR S141 OR S142 OR S143 OR S144 OR S145 OR S146 OR S147 OR S148 OR S149 OR S150 OR S151 OR S152)

S154 (MH "Prospective Studies") OR (MH "Cross Sectional Studies") OR (MH "Retrospective Design")

S155 (observational N5 (study or studies or design or analysis or analyses))

S156 (prospective N5 (study or studies or design or analysis or analyses))

S157 (retrospective N6 (study or studies or design or analysis or analyses or data)).

S158 ((longitudinal or longterm or long term) N6 (study or studies or design or analysis or analyses or data))

S159 (cross sectional N8 (study or studies or design or research or analysis or analyses or survey or findings))

S160 (associat* or regression* or correlat* or relat* or cohort*)

S161 S154 OR S155 OR S156 OR S157 OR S158 OR S159 OR S160

S162 (S7 AND S89 AND S153 AND S161)

**Search strategy in Scopus**

( TITLE-ABS-KEY ( ( hip W/4 ( osteoarthriti* OR arthriti* OR arthros* OR osteoarthrosi* ) ) OR "hip joint prosthes*" OR "hip prosthes*" OR tha OR ( hip W/4 ( remov* OR surg* OR replac* OR arthroplast* ) ) OR coxarthrosi* OR "hip implant*" OR "artificial hip joint*" ) ) AND ( ( TITLE-ABS-KEY ( "spin* align*" OR "vertebra* align*" OR "spin* column" OR "vertebra* column" OR "spinopelvic align*" OR "lumbosacral align*" OR "sagittal align*" OR "spine-hip relation*" OR "cervic* vertebra*" OR "thoracic* align*" OR "lumbar* align*" OR "sacral*align*" OR "hip* angle*" OR spinopelvic* OR alignment* OR ( ( pelvis* OR pelvic* OR hip* OR spin* ) W/4 alignment ) OR "cervical lordos*" OR "thoracic kyphos*" OR "lumbar lordos*" OR "lumbar scolios*" OR "T1 spinopelvic inclination*" OR "pelvic tilt*" OR "T1 pelvic*" OR "sacral slope*" OR "pelvic incidence*" OR "pelvic inclination*" OR "anterior pelvic plane*" OR "sagittal vertical axis*" OR spinosacral* OR "cup inclination*" OR "acetabular inclination*" OR "cup anteversion*" OR "acetabular anteversion*" ) ) OR ( TITLE-ABS-KEY ( "physical outcome*" OR "physical* measure*" OR "physical* assess*" OR "objective* outcome*" OR "objective* assess*" OR "objective* measure*" OR "physical function*" OR "function* measure*" OR "range of motion" OR rom OR schober OR ( finger* W/4 ( floor OR toe OR knee OR "fibular head" ) ) OR ( "wrist crease" W/3 floor ) OR "straight leg raise*" OR slr OR lasegue* OR "isometric strength" OR "isokinetic strength" OR "motor control" OR "movement control" OR "functional movement screen*" OR fms OR "sitting one leg knee extension" OR "posterior pelvic tilt" OR "waiter&apos;s bow" OR "one leg stance" OR "spatiotemporal gait" OR "spatio temporal gait" OR "stride length" OR "stride duration" OR "gait speed" OR cadence OR "gait asymmetry" OR "stance phase" OR "swing phase" OR "double limb support" OR "single limb support" OR inclinometer OR goniometer OR kyphometer OR "electromagnetic tracking" OR dynamometer OR "manual muscle test" OR medx OR cybex OR kin-com OR rehagait OR jamar OR "aerobic capacity" OR vo2* OR "bicycle ergomet*" OR "maximal graded exercise*" OR "steep ramp" OR "Biering Sorensen" OR "modified Sorensen" OR "Roman chair" OR ( sternum W/3 ( ground OR floor ) ) OR "muscle endurance" OR ( ( back OR body OR trunk ) W/3 endurance ) OR "prone bridge" OR ( ( ( "lower extremities" OR ( hips AND knees ) ) W/5 ( 90deg* OR "90 degree*" ) ) ) OR arch-up* OR sit-up* OR squat* OR "dumbbell press*" OR "double limb stance" OR "single limb stance" OR "stork stand*" OR "flamingo balance" OR y-balance OR ctsib OR ( clinical AND test* W/2 "sensory interaction" W/2 balance ) OR ( "clinical test*" W/2 "sensory integration" W/2 balance ) OR "Berg balance scale" OR "Katz Index of Independence in Activities of Daily Living" OR tinetti* OR "performance oriented mobility assessment*" OR "tandem walk*" OR "lower extremity motor coordination test*" OR ( chair W/3 ( stand* OR rise* ) ) OR "sit to stand" OR "stand up" OR "stand ups" OR roll* OR ( lie W/2 sit ) OR ( bed W/2 chair ) OR step* OR ( ( stand OR standing ) W/2 continuous ) OR "functional capacity evaluation" OR lift* OR "progressive isoinertial lifting evaluation" OR pile OR "forward reach" OR "functional reach" OR "self-paced walk*" OR "4-meter walk*" OR "4-metre walk*" OR "5-meter walk*" OR "5-metre walk*" OR "10-meter walk*" OR "10-metre walk*" OR "15-meter walk*" OR "15-metre walk*" OR "50-meter walk*" OR "50-metre walk*" OR "50-foot walk*" OR "5-minute walk*" OR "6-minute walk*" OR treadmill OR "overground walk*" OR ( walk W/3 hall* ) OR "shuttle walk*" OR ( stair* W/2 climb* ) OR "timed up and go" OR tug OR "8 foot up and go" OR "Physical capability assessment tool" OR pcat OR "aggregated functional performance test" OR "aggregated assessment of physical function" OR "short physical performance battery" OR "cumulated ambulation score" OR "functional independence measure" OR "activity measure for post-acute care 6 clicks" OR "activity measure for postacute care 6 clicks" OR "Physiotherapy functional mobility profile" OR "Barthel index" OR lying OR ( time W/5 stand* ) OR "constant postures" OR "active postures" OR "sedentary postures" OR "sedentary activity" OR "walking time" OR "walking distance" OR "claudication index" OR "walking speed" OR "daily walking events" OR "light intensity" OR "moderate intensity" OR "vigorous intensity" OR "activity count" OR "gait cycles" OR "gait posture index" OR "physical activity" OR acceleromet* OR "activity monitor" OR pedomet* OR gps OR watch OR smartwatch OR ( 6wt W/3 app* ) OR "muscle function" OR "muscle length" OR "muscle strength" OR "muscle activity" OR "EMG activity" OR "muscle activ*" OR "electromyography activ*" OR "muscle strength test*" OR "muscle strength grad*" OR "muscular* assess*" ) ) ) AND ( TITLE-ABS-KEY ( "Pain Measure*" OR disability* OR "patient satisfaction*" OR motivation* OR "Personal Satisfaction*" OR "Self Efficacy*" OR "Self Care*" OR "Social Behavior*" OR "Social Participation*" OR "Social Support*" OR "Social Isolation*" OR anxiety* OR depression* OR "Quality of Life" OR cogniti* OR kinesiophobia OR "Patient expect*" OR "Visual Analogue Scale" OR "Physical functioning patient reported outcome*" OR "treatment outcome*" OR "Patient Reported Outcome*" OR "Patient Outcome Assess*" OR "subjective* outcomes" OR "subjectively measured outcome*" OR "subjective assessment of outcome*" OR "patient reported*" OR "subjective* measure" OR prom* OR "Low* back* pain*" OR "low* back* ache*" OR backache* OR "lumbar vertebra* pain" OR "lumbar spin* pain" OR "lumbosacral pain" OR "lumbo-sacral pain" OR "lumbar* pain" OR lumbago* OR sciatica OR radiculopath* OR "radicular pain*" OR ( ( "health-related quality of life" OR hrqol ) W/2 questionnaire ) OR ( ( "Roland-Morris Disability" OR rmd ) W/2 questionnaire ) OR "Oswestry Disability Index" OR "Oxford Hip Score" OR "harris hip score" OR eq-5d OR sf-36* OR "hip disability and osteoarthritis outcome*" OR hoos* OR "Western Ontario and McMaster Universities Arthritis Index" OR womac OR "Numeric* Rating Scale" OR nrs OR "McGill Pain Questionnaire" OR "Tampa Scale of Kinesiophobia" OR "Beck Depression Inventory" OR "Nottingham Health Profile" OR "fear-avoidance beliefs questionnaire" OR "UCLA loneliness scale" OR "Japanese Orthopaedic Association Hip-Disease Evaluation Questionnaire" OR jheq OR "HOOS JR" OR "Forgotten Joint Score-12" OR fjs-12 OR "short questionnaire to assess health-enhancing physical activity" OR squash* OR "The Veterans RAND 12-Item Health Survey" OR vr-12* OR "Likert scale" OR "The Copenhagen Hip and Groin Outcome Score" OR hagos* OR "The pain and function of the hip" OR pfh* OR "the postoperative recovery profile" OR prp* OR "The Pain Disability Questionnaire" OR "Chronic Pain Self Efficacy Scale" OR "Pain Self-Efficacy Questionnaire" OR "Questionnaire for Physical Activity Decline in Pain" OR pad OR "Daily Activity Diary for Chronic Pain Patients" OR "The Pain Disability Index" OR "the Multidimensional Pain Inventory" OR "the Brief Pain Inventory" OR "The Impact on Participation and Autonomy" OR ipa OR "The Impact on Participation and Autonomy Questionnaire" OR ipaq OR "The Physical Activity Questionnaire" OR "The Quality of Well-Being Scale" OR qwb OR "The Sickness Impact Profile" OR sip OR "Work Limitations Questionnaire" OR wlq OR "Human Activity Profile" OR "Motor Fitness Scale" OR promis OR "Population Surveys of Chronic Disease and Disability" OR "the CDC HRQOL-14" OR "The Duke-UNC Health Profile" OR "Rosow Breslau Index of Mobility" OR "Health Assessment Questionnaire" OR " Functional Status Questionnaire" OR "Short Musculoskeletal Function Assessment Questionnaire" OR "Musculoskeletal Functional Limitation Index" OR "Patient-specific activity scoring scheme" ) ) AND ( TITLE-ABS-KEY ( cohort* OR ( observational W/5 ( study OR studies OR design OR analysis OR analyses ) ) OR ( prospective W/5 ( study OR studies OR design OR analysis OR analyses ) ) OR ( retrospective W/6 ( study OR studies OR design OR analysis OR analyses OR data ) ) OR ( ( longitudinal OR longterm OR "long term" ) W/6 ( study OR studies OR design OR analysis OR analyses OR data ) ) OR ( "cross sectional" W/8 ( study OR studies OR design OR research OR analysis OR analyses OR survey OR findings ) ) OR associat* OR regression* OR correlat* OR relat* ) )

**Search strategy in Web of Science**

(TS=((Hip NEAR/4 (osteoarthriti* or arthriti* or arthros* or osteoarthrosi*)) OR "hip joint prosthes*" or "hip prosthes*" or THA OR (hip NEAR/4 (remov* or surg* or replac* or arthroplast*)) OR coxarthrosi* OR "hip implant*" or "artificial hip joint*")) AND ((TS=("spin* align*" or "vertebra* align*" or "spin* column" or "vertebra* column" or "spinopelvic align*" or "lumbosacral align*" or "sagittal align*" OR "spine-hip relation*" or "cervic* vertebra*" or "thoracic* align*" or "lumbar* align*" or "sacral*align*" or "hip* angle*" or spinopelvic* or alignment* OR ((pelvis* or pelvic* or hip* or spin*) NEAR/4 alignment) OR "cervical lordos*" OR "thoracic kyphos*" OR "lumbar lordos*" OR "lumbar scolios*" OR "T1 spinopelvic inclination*" OR "pelvic tilt*" OR "T1 pelvic*" OR "sacral slope*" OR "pelvic incidence*" OR "pelvic inclination*" OR "anterior pelvic plane*" OR "sagittal vertical axis*" OR spinosacral* OR "cup inclination*" or "acetabular inclination*" OR "cup anteversion*" OR "acetabular anteversion*")) OR (TS=("physical outcome*" or "physical* measure*" or "physical* assess*" or "objective* outcome*" or "objective* assess*" or "objective* measure*" or "physical function*" or "function* measure*" OR "range of motion" or ROM OR Schober OR (finger* NEAR/4 (floor or toe or knee or "fibular head")) OR ("wrist crease" NEAR/3 floor) OR "straight leg raise*" or SLR or Lasegue* OR "isometric strength" or "isokinetic strength" OR "motor control" or "movement control" or "functional movement screen*" or FMS or "sitting one leg knee extension" or "posterior pelvic tilt" or "waiter’s bow" or "one leg stance" OR "spatiotemporal gait" or "spatio temporal gait" or "stride length" or "stride duration" or "gait speed" or cadence or "gait asymmetry" or "stance phase" or "swing phase" or "double limb support" or "single limb support" OR inclinometer or goniometer or kyphometer or "electromagnetic tracking" OR dynamometer or "manual muscle test" or MedX or Cybex or Kin-Com or RehaGait or JAMAR OR "aerobic capacity" or VO2* or "bicycle ergomet*" or "maximal graded exercise*" or "steep ramp" OR "Biering Sorensen" or "modified Sorensen" OR "Roman chair" OR (sternum NEAR/3 (ground or floor)) OR "muscle endurance" OR ((back or body or trunk) NEAR/3 endurance) OR "prone bridge" OR ((("lower extremities" or (hips OR knees)) NEAR/5 (90deg* or "90 degree*"))) OR arch-up* or sit-up* or squat* or "dumbbell press*" OR "double limb stance" or "single limb stance" or "stork stand*" or "flamingo balance" or y-balance OR CTSIB OR (“clinical test*” NEAR/2 "sensory interaction" NEAR/2 balance) OR ("clinical test*" NEAR/2 "sensory integration" NEAR/2 balance) OR "Berg balance scale" or Tinetti* or "performance oriented mobility assessment*" or "tandem walk*" OR "lower extremity motor coordination test*" OR (chair NEAR/3 (stand* or rise*)) OR "sit to stand" or "stand up" or "stand ups" or roll* OR (lie NEAR/2 sit) OR (bed NEAR/2 chair) OR step* OR ((stand or standing) NEAR/2 continuous) OR "functional capacity evaluation" OR lift* or "Katz Index of Independence in Activities of Daily Living" or "progressive isoinertial lifting evaluation" or pile OR "forward reach" or "functional reach" OR "self-paced walk*" or "4-meter walk*" or "4-metre walk*" or "5-meter walk*" or "5-metre walk*" or "10-meter walk*" or "10-metre walk*" or "15-meter walk*" or "15-metre walk*" or "50-meter walk*" or "50-metre walk*" or "50-foot walk*" or "5-minute walk*" or "6-minute walk*" or treadmill or "overground walk*" OR (walk NEAR/3 hall*) OR "shuttle walk*" OR (stair* NEAR/2 climb*) OR "timed up and go" or TUG or "8 foot up and go" OR "Physical capability assessment tool" or PCAT or "aggregated functional performance test" or "aggregated assessment of physical function" or "short physical performance battery" or "cumulated ambulation score" or "functional independence measure" OR "activity measure for post-acute care 6 clicks" or "activity measure for postacute care 6 clicks" OR "Physiotherapy functional mobility profile" or "Barthel index" OR lying OR (time NEAR/5 stand*) OR "constant postures" or "active postures" or "sedentary postures" or "sedentary activity" or "walking time" or "walking distance" or "claudication index" or "walking speed" or "daily walking events" or "light intensity" or "moderate intensity" or "vigorous intensity" or "activity count" or "gait cycles" or "gait posture index" or "physical activity" OR acceleromet* or "activity monitor" or pedomet* or GPS or watch or smartwatch OR (6WT NEAR/3 app*) OR "muscle function" or "muscle length" or "muscle strength" or "muscle activity" or "EMG activity" OR "muscle activ*" or "electromyography activ*" or "muscle strength test*" or "muscle strength grad*" or "muscular* assess*"))) AND (TS=("Pain Measure*" OR disability* OR "patient satisfaction*" OR motivation* OR "Personal Satisfaction*" OR "Self Efficacy*" OR "Self Care*" OR "Social Behavior*" OR "Social Participation*" OR "Social Support*" OR "Social Isolation*" OR Anxiety* OR Depression* OR “Quality of Life” OR Cogniti* OR Kinesiophobia OR "Patient expect*" OR "Visual Analogue Scale" OR "Physical functioning patient reported outcome*" OR "treatment outcome*" OR "Patient Reported Outcome*" OR "Patient Outcome Assess*" OR "subjective* outcomes" OR "subjectively measured outcome*" OR "subjective assessment of outcome*" OR "patient reported*" OR "subjective* measure" OR PROM* OR "Low* back* pain*" or "low* back* ache*" or backache* or "lumbar vertebra* pain" or "lumbar spin* pain" or "lumbosacral pain" or "lumbo-sacral pain" or "lumbar* pain" or lumbago* or sciatica or radiculopath* or "radicular pain*" OR (("health-related quality of life" or HRQOL) NEAR/2 questionnaire) OR (("Roland-Morris Disability" or RMD) NEAR/2 questionnaire) OR "Oswestry Disability Index" OR "Oxford Hip Score" OR "harris hip score" OR EQ-5D OR SF-36* OR "hip disability and osteoarthritis outcome*" or HOOS* OR "Western Ontario and McMaster Universities Arthritis Index" or WOMAC OR "Numeric* Rating Scale" or NRS OR "McGill Pain Questionnaire" OR "Tampa Scale of Kinesiophobia" OR "Beck Depression Inventory" OR "Nottingham Health Profile" OR "fear-avoidance beliefs questionnaire" OR "UCLA loneliness scale" OR "Japanese Orthopaedic Association Hip-Disease Evaluation Questionnaire" or JHEQ OR "HOOS JR" OR "Forgotten Joint Score-12" or FJS-12 OR "short questionnaire to assess health-enhancing physical activity" or SQUASH* OR "The Veterans RAND 12-Item Health Survey" or VR-12* OR "Likert scale" OR "The Copenhagen Hip and Groin Outcome Score" or HAGOS* OR "The pain and function of the hip" or PFH* OR "the postoperative recovery profile" or PRP* OR "The Pain Disability Questionnaire" OR "Chronic Pain Self Efficacy Scale" OR "Pain Self-Efficacy Questionnaire" OR "Questionnaire for Physical Activity Decline in Pain" or PAD OR "Daily Activity Diary for Chronic Pain Patients" OR "The Pain Disability Index" OR "the Multidimensional Pain Inventory" OR "the Brief Pain Inventory" OR "The Impact on Participation and Autonomy" or IPA OR "The Impact on Participation and Autonomy Questionnaire" or IPAQ OR "The Physical Activity Questionnaire" OR "The Quality of Well-Being Scale" or QWB OR "The Sickness Impact Profile" or SIP OR "Work Limitations Questionnaire" or WLQ OR "Human Activity Profile" OR "Motor Fitness Scale" OR PROMIS OR "Population Surveys of Chronic Disease and Disability" OR "the CDC HRQOL-14" OR "The Duke-UNC Health Profile" OR "Rosow Breslau Index of Mobility" OR "Health Assessment Questionnaire" OR "Functional Status Questionnaire " OR "Short Musculoskeletal Function Assessment Questionnaire" OR "Musculoskeletal Functional Limitation Index" OR "Patient-specific activity scoring scheme")) AND (TS=(cohort* OR (observational NEAR/5 (study or studies or design or analysis or analyses)) OR (prospective NEAR/5 (study or studies or design or analysis or analyses)) OR (retrospective NEAR/6 (study or studies or design or analysis or analyses or data)) OR ((longitudinal or longterm or "long term") NEAR/6 (study or studies or design or analysis or analyses or data)) OR ("cross sectional" NEAR/8 (study or studies or design or research or analysis or analyses or survey or findings)) OR associat* or regression* or correlat* or relat*))

**Search strategy in** [**ProQuest Dissertations & Theses Global**](https://www.proquest.com/pqdtglobal?accountid=15115)

(noft("hip osteoarthritis") OR noft("total hip arthroplasty") AND noft("spinal alignment" OR "pelvic alignment" OR spinopelvic alignment) OR noft("objective measures" OR physical functioning) AND noft(patient reported outcome))
