# Supplementary material for: Association between physical measures of spinopelvic alignment and physical functioning with patient reported outcome measures after total hip arthroplasty: Systematic review and narrative synthesis
Source: PLoS One. 2025 Dec 29;20(12):e0339615. doi: 10.1371/journal.pone.0339615 (PMC12747333; doi:10.1371/journal.pone.0339615)
Supplement: S3 Appendix — (DOCX) [file pone.0339615.s003.docx]

**Flow Chart: Retrieval of full-text articles**

Request for retrieval of n= 45 full-text articles via author contact or Western University library

**Outcomes of retrieval attempts:**

- Conference abstract or abstract of the included main article (n= 7)
- Conference abstracts or oral presentations only (n= 4)
- Authors responded, but the documents provided were not the full texts of the requested abstracts (n= 4)
- No response from authors (n= 16)
- Pilot study; unable to confirm eligibility. Contacted author for detailed information, but the message failed to deliver (n= 1)
- Delivery error (n= 1)
- Author affiliation not found; full text not located by Western University library (n= 3)
- Full text provided by the authors (n= 6)
- Full text located by Western University library (n= 3)

**Retrieval outcomes:**

- Retrieved 9 full texts
- Unable to retrieve 29 full texts

**Flow Chart: Verification of THA indication**

**Retrieval outcomes:**

- Included n= 11 studies
- Excluded n= 15 studies

Verification of THA indication via author contact (n= 26)

**Outcomes:**

- Author confirmed the indication was hip OA (n= 11)
- Author confirmed the indication included OA and other diagnoses (n= 3)
- Author was unable to confirm the indication (n= 2)
- Author could not confirm if the indication was primary OA only or both primary and secondary OA (n= 1)
- No response from authors (n= 9)

**Flow Chart: Request for Separate THA Results**

**Retrieval outcomes:**

- Included n= 2 studies
- Excluded n= 11 studies

Contact with study authors to obtain separate THA data (n= 13)

**Outcomes:**

- Separate THA results were not available (n= 5)
- Author confirmed THA-only results were the same but did not share data (n= 1)
- Author responded with other THA papers, not the requested study results (n= 1)
- Author sent separate THA results, but indications included OA and other diagnoses (n= 1)
- Delivery error (n= 3)
- Author provided separate THA results and confirmed primary hip OA as the indication (n= 1)
- Author provided separate results for THA (n= 1)
