# Supplementary material for: Association between physical measures of spinopelvic alignment and physical functioning with patient reported outcome measures after total hip arthroplasty: Systematic review and narrative synthesis
Source: PLoS One. 2025 Dec 29;20(12):e0339615. doi: 10.1371/journal.pone.0339615 (PMC12747333; doi:10.1371/journal.pone.0339615)
Supplement: S4 Appendix — (DOCX) [file pone.0339615.s004.docx]

| **Number** | **Author(s)** | **Title** | **Journal** | **Year** | **Included or excluded** | **Reason for exclusion** |
| --- | --- | --- | --- | --- | --- | --- |
|  | Luna et al., | Early patient-reported outcomes versus objective function after total hip and knee arthroplasty: a prospective cohort study | The bone & joint journal | 2017 | Included | Not applicable |
|  | Luna et al., | Objectively measured early physical activity after total hip or knee arthroplasty | Journal of clinical monitoring and computing | 2019 | Included | Not applicable |
|  | Heiberg et al., | Recovery and prediction of physical functioning outcomes during the first year after total hip arthroplasty | Archives of physical medicine and rehabilitation | 2013 | Included | Not applicable |
|  | Heiberg | Heiberg KE. RECOVERY OF PHYSICAL FUNCTIONING AFTER TOTAL HIP ARTHROPLASTY University of Oslo (URL: <https://www.duo.uio.no/bitstream/handle/10852/38225/dravhandling-heiberg.pdf?sequence=1>) | Doctoral thesis at University of Oslo | 2013 | Included | Not applicable |
|  | Abujaber | The relationships between physical impairments, functional limitations and movement asymmetries before and after total hip arthroplasty: A Longitudinal Study: University of Delaware (URL: <https://www.proquest.com/docview/1661456622?pq-origsite=gscholar&fromopenview=true&sourcetype=Dissertations%20&%20Theses> ) | ProQuest Dissertations & Theses | 2014 | Included | Not applicable |
|  | Biggs et al., | Gait function improvements, using Cardiff Classifier, are related to patient-reported function and pain following hip arthroplasty | Journal of orthopaedic research | 2022 | Included | Not applicable |
|  | Boardman et al., | The accuracy of assessing total hip arthroplasty outcomes: a prospective correlation study of walking ability and 2 validated measurement devices | The Journal of arthroplasty | 2000 | Included | Not applicable |
|  | Bolink et al., | Assessment of physical function following total hip arthroplasty: inertial sensor based gait analysis is supplementary to patient-reported outcome measures | Clinical Biomechanics | 2016 | Included | Not applicable |
|  | Cao et al., | Anatomical changes in lumbosacral vertebrae and their correlation with facet joint-derived low back pain in patients with hip osteoarthritis after total hip arthroplasty: a cohort study | Annals of Translational Medicine | 2022 | Included | Not applicable |
|  | Casartelli et al., | Reproducibility and validity of the physical activity scale for the elderly (PASE) questionnaire in patients after total hip arthroplasty | Physical therapy | 2015 | Included | Not applicable |
|  | Cinnamon et al., | Static and dynamic abductor function are both associated with physical function 1 to 5 years after total hip arthroplasty | [Clinical Biomechanics](https://www.sciencedirect.com/journal/clinical-biomechanics) | 2019 | Included | Not applicable |
|  | Davis et al., | The importance of range of motion after total hip arthroplasty | Clinical Orthopaedics and Related Research | 2007 | Included | Not applicable |
|  | Dayton et al., | Performance-Based Versus Self-Reported Outcomes Using the Hip Disability and Osteoarthritis Outcome Score After Total Hip Arthroplasty | American journal of physical medicine & rehabilitation | 2016 | Included | Not applicable |
|  | Eyvazov et al., | Effects of total hip arthroplasty on spinal sagittal alignment and static balance: a prospective study on 28 patients | European spine journal | 2016 | Included | Not applicable |
|  | Fallahzadeh et al., | Objective Activity Parameters Track Patient-specific Physical Recovery Trajectories After Surgery and Link With Individual Preoperative Immune States | Annals of surgery | 2023 | Included | Not applicable |
|  | Foucher et al., | Differences in preferred walking speeds in a gait laboratory compared with the real world after total hip replacement | Archives of physical medicine and rehabilitation | 2010 | Included | Not applicable |
|  | Foucher et al., | Hip abductor strength and fatigue are associated with activity levels more than 1 year after total hip replacement | Journal of Orthopaedic Research | 2018 | Included | Not applicable |
|  | Fujita et al., | Prospective study of physical activity and quality of life in Japanese women undergoing total hip arthroplasty | Journal of orthopaedic science | 2013 | Included | Not applicable |
|  | Fujita et al., | Analysis of factors influencing patient satisfaction after total hip arthroplasty in a Japanese cohort: the significant effect of postoperative physical activity | Journal of Physical Therapy Science | 2022 | Included | Not applicable |
|  | Goeb et al., | Early recovery outcomes in patients undergoing total hip arthroplasty through a posterior approach with modified postoperative precautions | The Journal of Arthroplasty | 2021 | Included | Not applicable |
|  | Harada et al., | Predictors of physical activity recovery after total hip arthroplasty: a prospective observational study | International Orthopaedics | 2024 | Included | Not applicable |
|  | Holm et al., | Surgery-induced changes and early recovery of hip-muscle strength, leg-press power, and functional performance after fast-track total hip arthroplasty: a prospective cohort study | PloS one | 2013 | Included | Not applicable |
|  | Holstege et al., | Preoperative quadriceps strength as a predictor for short-term functional outcome after total hip replacement | Archives of physical medicine and rehabilitation | 2011 | Included | Not applicable |
|  | Huang et al., | Step Length Asymmetry and Its Associations With Mechanical Energy Exchange, Function, and Fatigue After Total Hip Replacement | Journal of orthopaedic research | 2019 | Included | Not applicable |
|  | Jelsma et al., | Only limited correlations between patient-reported outcomes and objectively monitored physical activity 10-years after THA | Acta Orthopædica Belgica | 2021 | Included | Not applicable |
|  | Kamimura et al., | Preoperative predictors of ambulation ability at different time points after total hip arthroplasty in patients with osteoarthritis | Rehabilitation research and practice | 2014 | Included | Not applicable |
|  | Kaufmann et al., | Functional assessment of total hip arthroplasty using inertial measurement units: Improvement in gait kinematics and association with patient‐reported outcome measures | Journal of Orthopaedic Research | 2023 | Included | Not applicable |
|  | Kirschner et al., | Determination of Relationships between Symmetry-Based, Performance-Based, and Functional Outcome Measures in Patients Undergoing Total Hip Arthroplasty | Journal of Personalized Medicine | 2023 | Included | Not applicable |
|  | Kobayashi et al., | Effects of changes in whole-body alignment on ipsilateral knee pain after total hip arthroplasty | Journal of Orthopaedic Science | 2023 | Included | Not applicable |
|  | Lin et al., | Strong relationship of muscle force and fall efficacy, but not of gait kinematics, with number of falls in the year after Total Hip Arthroplasty for osteoarthritis: An exploratory study | Clinical biomechanics | 2022 | Included | Not applicable |
|  | Lindemann et al., | Gait analysis and WOMAC are complementary in assessing functional outcome in total hip replacement | Clinical rehabilitation | 2006 | Included | Not applicable |
|  | Lyman et al., | Monitoring patient recovery after THA or TKA using mobile technology | HSS Journal | 2020 | Included | Not applicable |
|  | Mahmood et al., | Association between changes in global femoral offset after total hip arthroplasty and function, quality of life, and abductor muscle strength | Acta orthopaedica | 2016 | Included | Not applicable |
|  | Mark‐Christensen et al., | Assessment of functional recovery after total hip and knee arthroplasty: An observational study of 95 patients | Musculoskeletal Care | 2019 | Included | Not applicable |
|  | McMeeken et al., | Impairment of muscle performance before and following total hip replacement | International Journal of Therapy and Rehabilitation | 2007 | Included | Not applicable |
|  | Meessen et al., | Association of handgrip strength with patient-reported outcome measures after total hip and knee arthroplasty | Rheumatology international | 2020 | Included | Not applicable |
|  | Melchiorri et al., | Late isometric assessment of hip abductor muscle and its relationship with functional tests in elderly women undergoing replacement of unilateral hip joint | American Journal of Physical Medicine & Rehabilitation | 2015 | Included | Not applicable |
|  | Moellenbeck et al., | Sedentary behavior in older patients before and after total hip arthroplasty: a prospective cohort study | Healthcare | 2020 | Included | Not applicable |
|  | Negrini et al., | The Importance of Cognitive Executive Functions in Gait Recovery After Total Hip Arthroplasty | Archives of Physical Medicine and Rehabilitation | 2020 | Included | Not applicable |
|  | Ochi et al., | Sagittal spinopelvic alignment predicts hip function after total hip arthroplasty | Gait Posture | 2017 | Included | Not applicable |
|  | Okamoto et al., | Worse Patient-Reported Outcomes and Spino-Pelvic Parameters After Total Hip Arthroplasty for Rapidly Progressive Osteoarthritis of the Hip Compared to Osteoarthritis: A Propensity-Matched Cohort Study | The Journal of Arthroplasty | 2024 | Included | Not applicable |
|  | Prüfer et al., | Responsiveness of Isokinetic Dynamometry in Patients with Osteoarthritis after Knee and Hip Arthroplasty: A Prospective Repeated-Measures Cohort Study | Healthcare | 2024 | Included | Not applicable |
|  | Qiu et al., | Preoperative prediction of early physical function in elder patients undergoing hip arthroplasty using a subjective physical activity questionnaire | Chinese Journal of Tissue Engineering Research | 2014 | Included | Not applicable |
|  | Segev-Jacubovski | Functional ability, psychological factors, and rehabilitation outcomes after elective total hip replacement | Canadian Journal of Occupational Therapy | 2023 | Included | Not applicable |
|  | Sliwinski et al., | Gait, quality of life, and their association following total hip arthroplasty | Journal of geriatric physical therapy | 2006 | Included | Not applicable |
|  | Tang et al., | Do physical activity and sleep correlate with patient-reported outcomes in total hip arthroplasty? | The Journal of Hip Surgery | 2021 | Included | Not applicable |
|  | Tolk et al., | Measurement properties of the OARSI core set of performance-based measures for hip osteoarthritis: a prospective cohort study on reliability, construct validity and responsiveness in 90 hip osteoarthritis patients | Acta orthopaedica | 2019 | Included | Not applicable |
|  | Tsukagoshi et al., | Factors associated with restricted hip extension during gait in women after total hip arthroplasty | Hip International | 2015 | Included | Not applicable |
|  | Tugay et al., | High independence level in functional activities reduces hospital stay after total hip arthroplasty regardless of pain intensity | Saudi medical journal | 2004 | Included | Not applicable |
|  | Vergari et al., | The relationship between spino-pelvic-hip mobility and quality of life before and after total hip arthroplasty | Archives of Orthopaedic and Trauma Surgery | 2024 | Included | Not applicable |
|  | Wada et al., | Gait Variability in Women With Hip Osteoarthritis Before and After Total Hip Replacement A Prospective Cohort Study | AMERICAN JOURNAL OF PHYSICAL MEDICINE & REHABILITATION | 2019 | Included | Not applicable |
|  | Wagenmakers et al., | Reliability and validity of the short questionnaire to assess health-enhancing physical activity (SQUASH) in patients after total hip arthroplasty | BMC Musculoskeletal Disorders | 2008 | Included | Not applicable |
|  | Yamaguchi et al., | Relationships between Change in Number of Steps and Changes in Physical Function, Pain, and Self Efficacy after Hospital Discharge Following Total Hip Arthroplasty (人工股関節全置換術術後患者における退院時から退院後 1 ヵ月の歩数変化量と身体機能, 疼痛, 自己効力感との関係) | Physical therapy science (理学療法科学) | 2019 | Included | Not applicable |
|  | Yuksel et al., | Relationships between performance-based tests and patient-based outcome measures in patients with total hip arthroplasty | HIP International | 2016 | Excluded | No full-text available |
|  | Lung et al., | Validation of the 2-minute and 6-minute walk tests as measures of walking capacity among inpatients receiving rehabilitation following total hip or knee arthroplasty | Archives of Physical Medicine and Rehabilitation | 2010 | Excluded | No full-text available |
|  | Bozgeyik et al., | Parameters affecting physical function in total hip artroplasty | Fizyoterapi Rehabilitasyon | 2018 | Excluded | No full-text available |
|  | Kinikli et al., | Factors effecting mobility following total hip arthroplasty | Fizyoterapi Rehabilitasyon | 2014 | Excluded | No full-text available |
|  | Bolink et al., | The association between radiographic and functional outcomes after tha | HIP International | 2018 | Excluded | No full-text available |
|  | Mandl et al., | Is frailty associated with adverse events after total joint arthroplasty? | Osteoarthritis and Cartilage | 2018 | Excluded | No full-text available |
|  | Bednarek et al., | [Functional result and telemetric evaluation of gait after total endoprosthetic arthroplasty of the hip] | Chirurgia narzadow ruchu i ortopedia polska | 1990 | Excluded | No full-text available |
|  | Nelson et al., | The Impact of Arthritis and Arthroplasty on a Group of Older Adults with Multiple Medical Conditions in Hopkins Elder Plus, a PACE Site in Maryland | Journal of the American Geriatrics Society | 2023 | Excluded | No full-text available |
|  | Robertson et al., | Investigation of anterior knee pain after total hip replacement: a pilot study | Physiotherapy research international | 2007 | Excluded | No full-text available  (The study lacked detail for eligibility assessment) |
|  | Lenguerrand et al., | The need for caution in the selection and interpretation of measures of function for patients with severe hip and knee problems | Osteoarthritis and Cartilage | 2014 | Excluded | No full-text available |
|  | Rottinger et al., | Early recovery of muscle power and proprioception in patients after total hip arthroplasty. a comparison of three different surgical approaches | HIP International | 2018 | Excluded | No full-text available |
|  | Eraslan et al., | The correlation between fear of falling and functional status in patients with total hip prosthesis | Fizyoterapi Rehabilitasyon | 2015 | Excluded | No full-text available |
|  | Piazzolla et al., | Hip-spine syndrome: The relationship between femoral neck anteversion, spinopelvic parameters and low back pain in patients with severe unilateral primary hip osteoarthritis | European Spine Journal | 2018 | Excluded | No full-text available |
|  | Gooberman-Hill et al., | Pain recovery following hip and knee replacement: A longitudinal study | Osteoporosis International | 2015 | Excluded | No full-text available |
|  | Daly et al., | An investigation of factors which predict acute short-term functional outcomes in older adults following hip replacement surgery | Irish Journal of Medical Science | 2014 | Excluded | No full-text available |
|  | Jones et al., | The relationship of range of motion and the WOMAC with total hip (THA) and knee arthroplasties (TKA) | ARTHRITIS AND RHEUMATISM | 1999 | Excluded | No full-text available |
|  | Cekmece et al., | Does handgrip strength affect kinesiophobia in patients with total hip arthroplasty | HIP International | 2016 | Excluded | No full-text available |
|  | Smith et al., | Duration of physical activity following total hip or knee replacement | International Journal of Therapy & Rehabilitation | 2016 | Excluded | No full-text available |
|  | Simank et al., | [Diagnosis of hip prosthesis loosening--sensitivity of clinical parameters] | Zeitschrift fur Orthopadie und Ihre Grenzgebiete | 1998 | Excluded | No full-text available |
|  | Green et al., | Postoperative ambulation in patients undergoing total hip arthroplasty, total knee arthroplasty and elective lumbar spine surgery to treat arthritic pathologies | Spine Journal | 2015 | Excluded | No full-text available |
|  | Cinnamon et al., | Hip abductor strength is associated with both performance test-based and self-reported function after total hip replacement | Journal of Orthopaedic Research | 2017 | Excluded | No full-text available |
|  | Barroso et al., | Risk factors for pain after total joint replacement in osteoarthritis: Different pain measures, distinct predictors | Arthritis and Rheumatology | 2019 | Excluded | No full-text available |
|  | Kobayashi et al., | Evaluation of the effect of total hip arthroplasty on spine-hip-knee alignment and pain using eos system | Journal of Orthopaedic Research | 2017 | Excluded | No full-text available |
|  | Wang et al., | [Effects of femoral offset on soft tissue balance in total hip arthroplasty] | Zhongguo gu shang = China journal of orthopaedics and traumatology | 2008 | Excluded | No full-text available |
|  | Pozzi et al., | Preoperative predictors of postoperative function in individuals undergoing total hip arthroplasty | Osteoarthritis and Cartilage | 2014 | Excluded | No full-text available |
|  | Kwapisz et al., | Evaluation of correlation between the grade of the heterotopic ossification, activity of bone morphogenetic protein-4 and the function of the hip joint in patients after cementless total hip arthroplasty | Bone | 2010 | Excluded | No full-text available |
|  | Tokura | Kinesiologic and kinematic analysis of walking pattern in osteoarthritis of the hip (Japanese) | The Journal of the Japanese Orthopaedic Association. | 1974 | Excluded | No full-text available |
|  | Pollet et al., | Does gait kinematic parameters change as functional outcome scales in total hip arthroplasty subjects after rehabilitation? | Gait and Posture | 2019 | Excluded | No full-text available |
|  | Heitzer et al., | Prediction of clinical orthopaedic scores based on instrumental gait analysis | Gait and Posture | 2022 | Excluded | No full-text available |
|  | Behery et al., | Age, gender, and body mass index do not explain individual variability in clinical and gait recovery after total hip arthroplasty | Osteoarthritis and Cartilage | 2013 | Excluded | Conference abstract of the included main article |
|  | Mundermann et al., | Changes in gait kinematics after hip arthroplasty assessed using wearable sensors are associated with PROMs (11315) | Swiss Medical Weekly | 2022 | Excluded | Conference abstract of the included main article |
|  | Foucher | Sex-specific patterns of gait and functional recovery from total hip arthroplasty | Journal of Orthopaedic Research | 2016 | Excluded | Conference abstract of the included main article |
|  | Ozcadirci et al., | Relationship between functional performance of artificial joint forgetting ability of individuals who underwent total hip arthroplasty surgery: Pilot study | Fizyoterapi Rehabilitasyon | 2018 | Excluded | Abstract of the included main article |
|  | Brunner et al., | Sex-specific associations between improvement in gait mechanics and improvement in pain, function, and abductor strength after total hip arthroplasty | Arthritis and Rheumatology | 2016 | Excluded | Abstract of the included main article |
|  | Nuesch et al., | TOWARDS WEARABLE SENSOR BASED GAIT ANALYSIS IN ROUTINE CLINICAL PRACTICE: ASSOCIATION BETWEEN GAIT KINEMATICS AND PATIENT REPORTED OUTCOMES IN HIP ARTHROPLASTY | Osteoarthritis and Cartilage | 2022 | Excluded | Conference abstract of the included main article |
|  | Biggs et al., | Preoperative gait biomechanics and its relationship to functional outcome following total hip arthroplasty | Osteoarthritis and Cartilage | 2019 | Excluded | Abstract of the included main article |
|  | Jonkers et al., | Relation between subject-specific hip joint loading, stress distribution in the proximal femur and bone mineral density changes after total hip replacement | Journal of biomechanics | 2008 | Excluded | Outcome |
|  | Bragdon et al., | Minimum 6-year followup of highly cross-linked polyethylene in THA | Clinical Orthopaedics and Related Research | 2007 | Excluded | Outcome |
|  | Elings et al., | Development of a Risk Stratification Model for Delayed Inpatient Recovery of Physical Activities in Patients Undergoing Total Hip Replacement | Journal of Orthopaedic & Sports Physical Therapy | 2016 | Excluded | Outcome |
|  | Hafkamp et al., | The Relationship Between Psychological Aspects and Trajectories of Symptoms in Total Knee Arthroplasty and Total Hip Arthroplasty | The Journal of Arthroplasty | 2021 | Excluded | Outcome |
|  | Judgee et al., | Clinical tool to identify patients who are most likely to achieve long-term improvement in physical function after total hip arthroplasty | Arthritis care & research | 2012 | Excluded | Outcome |
|  | Eguchi et al., | Spinopelvic Alignment and Low Back Pain after Total Hip Replacement Arthroplasty in Patients with Severe Hip Osteoarthritis | Asian Spine Journal | 2018 | Excluded | Outcome |
|  | Nankaku et al., | Gait analysis of patients in early stages after total hip arthroplasty: effect of lateral trunk displacement on walking efficiency | Journal of Orthopaedic Science | 2007 | Excluded | Outcome |
|  | Stecz et al., | Physical activity, stress, coping and life satisfaction in patients undergoing total hip replacement | Sport and Quality of Life | 2013 | Excluded | Outcome |
|  | Riddle et al., | Preoperative Risk Factors for Postoperative Falls in Persons Undergoing Hip or Knee Arthroplasty: A Longitudinal Study of Data From the Osteoarthritis Initiative | Archives of Physical Medicine & Rehabilitation | 2018 | Excluded | Outcome |
|  | Kiefer et al., | Functional performance and grip strength after total hip replacement | Occupational Therapy in Health Care | 2004 | Excluded | Outcome |
|  | Ninomiya et al., | Prevalence of frailty and associated factors among community-dwelling older adults after total hip arthroplasty | HIP International | 2023 | Excluded | Outcome |
|  | Wang et al., | Is physical activity a risk factor for primary knee or hip replacement due to osteoarthritis? A prospective cohort study | The Journal of rheumatology | 2011 | Excluded | Outcome |
|  | Gardes et al., | Long-term review of a homogeneous, consecutive series of 100 Charnley's total hip prostheses | Revue de Chirurgie Orthopedique et Reparatrice de L'appareil Moteur | 1996 | Excluded | Outcome |
|  | Zasadzka et al., | Does Hand Grip Strength (HGS) Predict Functional Independence Differently in Patients Post Hip Replacement Due to Osteoarthritis versus Patients Status Post Hip Replacement Due to a Fracture? | Clinical Interventions in Aging | 2023 | Excluded | Outcome |
|  | Ozden et al., | The test-retest reliability, concurrent validity and minimal detectable change of the 3-m backward walking test in patients with total hip arthroplasty | Journal of Arthroscopy and Joint Surgery | 2021 | Excluded | Outcome |
|  | Goyal et al., | Effect of Acetabular Component Positioning on Functional Outcomes in Primary Total Hip Arthroplasty | The Journal of Arthroplasty | 2017 | Excluded | Outcome |
|  | Rizk et al., | Radiographic parameters of pelvic and proximal femoral morphology do not predict outcomes for direct anterior total hip arthroplasty | Injury | 2023 | Excluded | Outcome |
|  | Nagai et al., | Fear of falling during activities of daily living after total hip arthroplasty in Japanese women: a cross-sectional study | Physiotherapy | 2014 | Excluded | Outcome |
|  | Nanri et al., | Preoperative malnutrition is a risk factor for delayed recovery of mobilization after total hip arthroplasty | PM&R | 2021 | Excluded | Outcome |
|  | Neuprez et al., | Total joint replacement improves pain, functional quality of life, and health utilities in patients with late-stage knee and hip osteoarthritis for up to 5 years | Clinical rheumatology | 2019 | Excluded | Outcome |
|  | Naylor et al., | Participation in Regular Physical Activity After Total Knee or Hip Arthroplasty for Osteoarthritis: Prevalence, Associated Factors, and Type | Arthritis care & research | 2019 | Excluded | Outcome |
|  | Giuseppe et al., | Ceramic-on-ceramic versus ceramic-on-polyethylene in total hip arthroplasty: a comparative study at a minimum of 13 years follow-up | BMC Musculoskeletal Disorders | 2021 | Excluded | Outcome |
|  | Chisato Matsumoto et al., | 女性変形性股関節症患者の術前後の 歩容の自己評価と心理社会的側面の検討 一人工股関節全置換術患者と低侵襲寛骨臼骨切り術患者の比較一 | Journal of Japan Academy of Nursing Science | 2018 | Excluded | Outcome |
|  | Kang, Mi Kyung et al., | 하지 인공관절 치환술 환자의 건강 관련 삶의 질 영향요인 | Journal of Korean Academy of Fundamentals of Nursing | 2017 | Excluded | Outcome |
|  | Ozden et al., | The test-retest reliability and concurrent validity of the five times sit to stand test and step test in older adults with total hip arthroplasty | Experimental gerontology | 2020 | Excluded | Outcome |
|  | Tilbury et al., | Patients' pre-operative general and specific outcome expectations predict postoperative pain and function after total knee and total hip arthroplasties | Scandinavian journal of pain | 2018 | Excluded | Outcome |
|  | Mortati et al., | Functional and radiographic evaluation and quality of life analysis after cementless total hip arthroplasty with ceramic bearings: minimum of 5 years follow-up | Revista Brasileira de Ortopedia | 2013 | Excluded | Outcome |
|  | Cech et al., | Pre-operative planning in THA. Part III: do implant size prediction and offset restoration influence functional outcomes after THA? | Archives of orthopaedic and trauma surgery | 2020 | Excluded | Outcome |
|  | Tateiwa et al., | Early Postoperative Change in Hip Rotation Angle and Factors Contributing to It for Patients Undergoing Total Hip Arthroplasty | Orthopedics | 2024 | Excluded | Outcome |
|  | Foucher et al., | Preoperative gait adaptations persist one year after surgery in clinically well-functioning total hip replacement patients | Journal of biomechanics | 2007 | Excluded | Outcome |
|  | Nawatthakul et al., | The ability and factors related with floor sitting after total hip arthroplasty with a posterolateral approach | Orthopedic Reviews | 2022 | Excluded | Outcome |
|  | Peter et al., | The association between comorbidities and pain, physical function and quality of life following hip and knee arthroplasty | Rheumatology international | 2015 | Excluded | Outcome |
|  | De Caro et al., | OPTIMAL IMPROVEMENT IN FUNCTION AFTER TOTAL HIP AND KNEE REPLACEMENT: HOW DEEP DO YOU KNOW YOUR PATIENT'S MIND? | Journal of Biological Regulators and Homeostatic Agents | 2015 | Excluded | Outcome |
|  | Wylde et al., | Post-operative radiographic factors and patient-reported outcome after total hip replacement | Hip International | 2012 | Excluded | Outcome |
|  | Singh et al., | Predictors of activity limitation and dependence on walking aids after primary total hip arthroplasty | Journal of the American Geriatrics Society | 2010 | Excluded | Outcome |
|  | Raab et al., | Do we still need to screen our patients?-Orthopaedic scoring based on motion tracking | International Orthopaedics | 2023 | Excluded | Outcome |
|  | Davis et al., | Understanding recovery: Changes in the relationships of the International Classification of Functioning (ICF) components over time | Social Science & Medicine | 2012 | Excluded | Outcome |
|  | Fischer et al., | Preoperative factors improving the prediction of the postoperative sagittal orientation of the pelvis in standing position after total hip arthroplasty | Scientific reports | 2020 | Excluded | Outcome |
|  | Oosting et al., | Preoperative prediction of inpatient recovery of function after total hip arthroplasty using performance-based tests: a prospective cohort study | Disability and Rehabilitation | 2016 | Excluded | Outcome |
|  | Iorio et al., | Preoperative demand matching is a valid indicator of patient activity after total hip arthroplasty | The Journal of arthroplasty | 2004 | Excluded | Outcome |
|  | Nankaku et al., | Preoperative Prediction of Ambulatory Status at 6 Months After Total Hip Arthroplasty | Physical Therapy | 2013 | Excluded | Outcome |
|  | Singh et al., | Ipsilateral lower extremity joint involvement increases the risk of poor pain and function outcomes after hip or knee arthroplasty | BMC Medicine | 2013 | Excluded | Outcome |
|  | Suparb Aree-Ue et al., | Factors Predicting Functional Ability among Older Adults undergoing Hip and Knee Arthroplasty | Pacific Rim International Journal of Nursing Research | 2019 | Excluded | Outcome |
|  | Meessen et al., | Patients who underwent total hip or knee arthroplasty are more physically active than the general Dutch population | Rheumatology international | 2017 | Excluded | Outcome |
|  | Shadyab et al., | Association of leisure-time physical activity with late-life mobility limitation among women with total joint replacement for hip or knee osteoarthritis | Arthritis and Rheumatology | 2017 | Excluded | Outcome |
|  | LeBrun et al., | The Risk Assessment and Prediction Tool (RAPT) Score Predicts Discharge Destination, Length of Stay, and Postoperative Mobility After Total Joint Arthroplasty | The Journal of Arthroplasty | 2023 | Excluded | Outcome |
|  | ÖZÇADIRCI et al., | TOTAL KALÇA ARTROPLASTİSİ CERRAHİSİ GEÇİRMİŞ BİREYLERİN HAREKET ETME KORKULARININ DUYGU DURUM DÜZEYLERİYLE İLİŞKİSİ (URL link: <https://avesis.hacettepe.edu.tr/yayin/9f937bdf-e25a-4023-a05f-1a3a657e1fd3/total-kalca-artroplasti-cerrahisi-gecirmis-bireylerin-hareket-etme-korkularinin-duygu-durum-duzeyleriyle-iliskisi>) | 10. Uluslararası Protez-Ortez Kongresi (10th International Prosthetics-Orthosis Congress) | 2018 | Excluded | Outcome |
|  | Okamoto et al., | Clinical significance of relative pelvic version measurement as a predictor of low back pain after total hip arthroplasty | European Spine Journal | 2023 | Excluded | Outcome |
|  | Foucher et al., | Preoperative factors associated with postoperative gait kinematics and kinetics after total hip arthroplasty | Osteoarthritis and Cartilage | 2015 | Excluded | Population |
|  | van den Akker-Scheek et al., | Preoperative or postoperative self-efficacy: which is a better predictor of outcome after total hip or knee arthroplasty? | Patient education and counseling | 2007 | Excluded | Population |
|  | Blikman et al., | Reliability and validity of the Dutch version of the International Physical Activity Questionnaire in patients after total hip arthroplasty or total knee arthroplasty | Journal of orthopaedic & sports physical therapy | 2013 | Excluded | Population |
|  | Kawai et al., | Patient-and surgery-related factors that affect patient-reported outcomes after total hip arthroplasty | Journal of clinical medicine | 2018 | Excluded | Population |
|  | Matsunaga-Myoji et al., | Three-Year Follow-Up Study of Physical Activity, Physical Function, and Health-Related Quality of Life After Total Hip Arthroplasty | The Journal of Arthroplasty | 2020 | Excluded | Population |
|  | Ozcadirci et al., | Are Kinesiophobia and Functional Performance Related to Ability to Forget the Artificial Joint in Patients with Total Hip Arthroplasty? | Clinical and Experimental Health Sciences | 2021 | Excluded | Population |
|  | Vissers et al., | Patient satisfaction after a total hip or knee arthroplasty | Osteoarthritis and Cartilage | 2009 | Excluded | Population |
|  | Sechriest et al., | Activity Level in Young Patients With Primary Total Hip Arthroplasty. A 5-Year Minimum Follow-up | Journal of Arthroplasty | 2007 | Excluded | Population |
|  | Brownlow et al., | Disability and mental health of patients waiting for total hip replacement | Annals of the Royal College of Surgeons of England | 2001 | Excluded | Population |
|  | Kawai et al., | Association Between the Amount of Limb Lengthening and Hip Range of Motion After Total Hip Arthroplasty | The Journal of the American Academy of Orthopaedic Surgeons | 2022 | Excluded | Population |
|  | Murao et al., | Reproducibility, criterion-related validity, and minimal clinically important difference of the stair negotiation test after total Hip arthroplasty | Physiotherapy Theory and Practice | 2022 | Excluded | Population |
|  | Jo et al., | Comparison of Balance, Proprioception and Skeletal Muscle Mass in Total Hip Replacement Patients With and Without Fracture: A Pilot Study | Annals of Rehabilitation Medicine | 2016 | Excluded | Population |
|  | Ikeda et al., | Effects of perioperative factors and hip geometry on hip abductor muscle strength during the first 6 months after anterolateral total hip arthroplasty | Journal of Physical Therapy Science | 2017 | Excluded | Population |
|  | Sant'Anna et al., | A wearable gait analysis system using inertial sensors Part II: Evaluation in a clinical setting | International Conference on Bio-inspired Systems and Signal Processing | 2012 | Excluded | Population |
|  | Matsunaga-Myoji et al., | Propensity score-matched comparison of physical activity and quality of life between revision total hip arthroplasty and primary total hip arthroplasty | Journal of Orthopaedics | 2023 | Excluded | Population |
|  | Hjorth et al., | Block-step asymmetry 5 years after large-head metal-on-metal total hip arthroplasty is related to lower muscle mass and leg power on the implant side | Clinical Biomechanics | 2014 | Excluded | Population |
|  | Höll et al., | Clinical outcome and physical activity measured with StepWatch 3 TM Activity Monitor after minimally invasive total hip arthroplasty | Journal of orthopaedic surgery and research | 2018 | Excluded | Population |
|  | Jogi et al., | Comparisons of clinically based outcome measures and laboratory-based outcome measure for balance in patients following total hip and knee arthroplasty | Orthopedic research and reviews | 2017 | Excluded | Population |
|  | Kuhn et al., | Total hip arthroplasty in patients 50 years or less. Do we improve activity profiles? | The Journal of arthroplasty | 2013 | Excluded | Population |
|  | Kawano et al., | Development of a Clinical Prediction Rule to Identify Physical Activity After Total Hip Arthroplasty | Archives of Physical Medicine and Rehabilitation | 2022 | Excluded | Population |
|  | Long et al., | Low Grip Strength Associated with Clinical Outcomes after Total Hip Arthroplasty - A Prospective Case-Control Study | Orthopaedic Surgery | 2021 | Excluded | Population |
|  | Wollmerstedt et al., | [A novel questionnaire to assess activity in patients after hip arthroplasties] | Der Orthopäde | 2006 | Excluded | Population |
|  | Kawai et al., | Discrepancy in the Responsiveness to Hip Range of Motion Between Harris and Oxford Hip Scores | Arthroplasty Today | 2022 | Excluded | Population |
|  | Lee et al., | Correlation between Harris hip score and gait analysis through artificial intelligence pose estimation in patients after total hip arthroplasty | Asian Journal of Surgery | 2023 | Excluded | Population |
|  | Alvarez et al., | Correlation between the level of physical activity as measured by accelerometer and the Harris Hip Score | Hip International | 2015 | Excluded | Population |
|  | Shiomoto et al., | Objective Activity Levels and Patient-Reported Outcomes After Total Hip Arthroplasty and Periacetabular Osteotomy: Retrospective Matched Cohort Study at Mean 12-Year Follow-Up | The Journal of Arthroplasty | 2023 | Excluded | Population |
|  | John et al., | Relationship Between Self-Reported Function, Functional Tests and Biomechanical Parameters in Patients 12 Months After Total Hip Arthroplasty: A Preliminary Cross-Sectional Study | Indian journal of orthopaedics | 2023 | Excluded | Population |
|  | Zahiri et al., | Assessing activity in joint replacement patients | The Journal of arthroplasty | 1998 | Excluded | Population |
|  | Trudelle-Jackson et al., | Outcomes of total hip arthroplasty: a study of patients one year postsurgery | Journal of Orthopaedic & Sports Physical Therapy | 2002 | Excluded | Population |
|  | van den Akker-Scheek et al., | Physical functioning before and after total hip arthroplasty: perception and performance | Physical therapy | 2008 | Excluded | Population |
|  | Wollmerstedt et al., | [Design and evaluation of the Extra Short Musculoskeletal Function Assessment Questionnaire XSMFA-D] | Zeitschrift fur Orthopadie und ihre Grenzgebiete | 2003 | Excluded | Population |
|  | Moellenbeck et al., | Does Total Hip or Knee Arthroplasty Have an Effect on the Patients' Functional or Behavioral Outcome and Health-Related Quality of Life of the Affected Partners? | The Journal of arthroplasty | 2021 | Excluded | Population |
|  | Ridge et al., | The relationship between multidisciplinary discharge outcomes and functional status after total hip replacement | Orthopaedic Nursing | 2000 | Excluded | Population |
|  | Stratford et al., | Performance measures were necessary to obtain a complete picture of osteoarthritic patients | Journal of clinical epidemiology | 2006 | Excluded | Population |
|  | Kennedy et al., | Comparison of gender and group differences in self-report and physical performance measures in total hip and knee arthroplasty candidates | The Journal of arthroplasty | 2002 | Excluded | Population |
|  | Murofushi et al., | The relationship between physical function and JHEQ at 6 months after total hip arthroplasty | Rigakuryoho Kagaku | 2016 | Excluded | Population |
|  | Marks et al., | Comorbid disease profiles of adults with end-stage hip osteoarthritis | Medical Science Monitor | 2002 | Excluded | Population |
|  | Vasarhelyi et al., | Assessing Preoperative Mobility in Total Hip Arthroplasty: A SAFE T database study | Journal of Orthopaedics | 2019 | Excluded | Population |
|  | Martinez-Ramirez et al., | Preoperative ambulatory measurement of asymmetric leg loading during sit-to-stand in hip arthroplasty patients | IEEE Transactions on Neural Systems and Rehabilitation Engineering | 2013 | Excluded | Population |
|  | McGrory et al., | Correlation of measured range of hip motion following total hip arthroplasty and responses to a questionnaire | The Journal of arthroplasty | 1996 | Excluded | Population |
|  | Okamoto et al., | The psoas muscle index as a useful predictor of total hip arthroplasty outcomes | Archives of Orthopaedic and Trauma Surgery | 2024 | Excluded | Population |
|  | Matsuyama et al., | Factors associated with the progression of sagittal spinal deformity after total hip arthroplasty: a propensity score-matched cohort study | International Orthopaedics | 2024 | Excluded | Population |
|  | Bernad-Pineda et al., | [Quality of life in patients with knee and hip osteoarthritis] | Revista Española de Cirugía Ortopédica y Traumatología | 2014 | Excluded | Population |
|  | Holm et al., | Thigh and Knee Circumference, Knee-Extension Strength, and Functional Performance After Fast-Track Total Hip Arthroplasty | PM&R | 2011 | Excluded | Population |
|  | Prudnikova et al., | Biomechanical aspects of spine sagittal balance in patients with coxarthrosis in total hip replacement (preliminary study) | Genij Ortopedii | 2019 | Excluded | Population |
|  | Luo et al., | Preoperative sleep quality affects postoperative pain and function after total joint arthroplasty: a prospective cohort study | Journal of orthopaedic surgery and research | 2019 | Excluded | Population |
|  | Buker et al., | Is quality of life related to risk of falling, fear of falling, and functional status in patients with hip arthroplasty? | Physiotherapy research international | 2019 | Excluded | Population |
|  | Ponds et al., | Wearable sensor-based measures of step-up transfers are supplementary to patient-reported outcome measures following total joint arthroplasty | Disability and Rehabilitation | 2023 | Excluded | Population |
|  | Unnanuntana et al., | Performance-based tests and self-reported questionnaires provide distinct information for the preoperative evaluation of total hip arthroplasty patients | The Journal of arthroplasty | 2012 | Excluded | Population |
|  | Tang et al., | Chinese patients' satisfaction with total hip arthroplasty: what is important and dissatisfactory? | The Journal of arthroplasty | 2014 | Excluded | Population |
|  | Poitras et al., | Predicting hospital length of stay and short-term function after hip or knee arthroplasty: are both performance and comorbidity measures useful? | International Orthopaedics | 2018 | Excluded | Population |
|  | Özçadırcı et al., | Range of motion and muscle strength deficits of patients with total hip arthroplasty after surgery | Baltic Journal of Health and Physical Activity | 2021 | Excluded | Population |
|  | Orsi et al., | Patient reported outcome measures correlate with step-count in total hip arthroplasty | Technology and Health Care | 2024 | Excluded | Population |
|  | Martinez-Ramirez et al., | Pre-operative ambulatory measurement of asymmetric lower limb loading during walking in total hip arthroplasty patients | Journal of neuroengineering and rehabilitation | 2013 | Excluded | Population |
|  | Morri et al., | Is kinesiophobia a predictor of early functional performance after total hip replacement? A prospective prognostic cohort study | BMC musculoskeletal disorders | 2020 | Excluded | Population |
|  | Bendich et al., | Changes in prospectively collected longitudinal patient-generated health data are associated with short-term patient-reported outcomes after total joint arthroplasty: a pilot study | Arthroplasty Today | 2019 | Excluded | Population |
|  | Kurihara et al., | Early postoperative relationship between patient-reported outcome measures and gait biomechanical factors after total hip arthroplasty | Gait & Posture | 2022 | Excluded | Population |
|  | Schmidt-Braekling et al., | Spinal pathology and outcome post-THA: does segment of arthrodesis matter? | Archives of Orthopaedic and Trauma Surgery | 2022 | Excluded | Population |
|  | Crizer et al., | Stepping Toward Objective Outcomes: A Prospective Analysis of Step Count After Total Joint Arthroplasty | The Journal of arthroplasty | 2017 | Excluded | Population |
|  | Vaz et al., | Isometric hip abductor strength following total hip replacement and its relationship to functional assessments | Journal of Orthopaedic and Sports Physical Therapy | 1993 | Excluded | Population |
|  | Kästner et al., | The virtue of optimistic realism - expectation fulfillment predicts patient-rated global effectiveness of total hip arthroplasty | BMC Musculoskeletal Disorders | 2021 | Excluded | Population |
|  | Okuzu et al., | Preoperative Factors Associated With Low Back Pain Improvement After Total Hip Arthroplasty in a Japanese Population | The Journal of arthroplasty | 2022 | Excluded | Population |
|  | Wollmerstedt et al., | The Daily Activity Questionnaire: A Novel Questionnaire to Assess Patient Activity After Total Hip Arthroplasty | The Journal of arthroplasty | 2010 | Excluded | Population |
|  | Elibol et al., | Relationship between self-reported and performance-based tests in assessment of patients with total hip arthroplasty | Hip International | 2018 | Excluded | Population |
|  | Bryant et al., | A statistical analysis of hip scores | The Journal of Bone & Joint Surgery | 1993 | Excluded | Population |
|  | Barroso et al., | Prognostics for pain in osteoarthritis: Do clinical measures predict pain after total joint replacement? | PLoS One | 2020 | Excluded | Population |
|  | Erlenwein et al., | The Influence of Chronic Pain on Postoperative Pain and Function After Hip Surgery: A Prospective Observational Cohort Study | The Journal of Pain | 2016 | Excluded | Analysis |
|  | Zeni et al., | The Effect of Surgical Approach on Strength and Function after Total Hip Arthroplasty | Delaware Medical Journal | 2016 | Excluded | Analysis |
|  | Hjorth et al., | Physical Activity Is Associated With the Level of Chromium but Not With Changes in Pseudotumor Size in Patients With Metal-on-Metal Hip Arthroplasty | The Journal of Arthroplasty | 2018 | Excluded | Analysis |
|  | Ieiri et al., | What predicts 36-item health survey version 2 after total hip arthroplasty | Archives of physical medicine and rehabilitation | 2013 | Excluded | Analysis |
|  | Horstmann et al., | Minimizing Preoperative and Postoperative Limping in Patients After Total Hip Arthroplasty Relevance of Hip Muscle Strength and Endurance | American journal of physical medicine & rehabilitation | 2013 | Excluded | Analysis |
|  | Kennedy et al., | Using outcome measure results to facilitate clinical decisions the first year after total hip arthroplasty | Journal of orthopaedic & sports physical therapy | 2011 | Excluded | Analysis |
|  | Bolink et al., | The association of leg length and offset reconstruction after total hip arthroplasty with clinical outcomes | Clinical Biomechanics | 2019 | Excluded | Analysis |
|  | Lazennec et al., | Patient perception of leg length after total hip arthroplasty does not correlate with sagittal lumbar spine stiffness, history of spinal pathology or fusion | International Orthopaedics | 2023 | Excluded | Analysis |
|  | Lavernia et al., | High Rates of Interest in Sex in Patients With Hip Arthritis | Clinical Orthopaedics and Related Research | 2016 | Excluded | Analysis |
|  | Eilander et al., | The short external rotators in the anterior approach hip arthroplasty: do the tendons heal or not? A prospective MRI study | Hip International | 2023 | Excluded | Analysis |
|  | Colgan et al., | Gait analysis and hip extensor function early post total hip replacement | Journal of Orthopaedics | 2016 | Excluded | Analysis |
|  | Świtoń et al., | Activity and Quality of Life after Total Hip Arthroplasty | Ortopedia Traumatologia Rehabilitacja | 2017 | Excluded | Analysis |
|  | Vigdorchik et al., | Does Low Back Pain Improve Following Total Hip Arthroplasty? | The Journal of Arthroplasty | 2022 | Excluded | Analysis |
|  | Röder et al., | Influence of preoperative functional status on outcome after total hip arthroplasty | The Journal of bone and joint surgery | 2007 | Excluded | Analysis |
|  | Mahon et al., | Health-related quality of life and mobility of patients awaiting elective total hip arthroplasty: a prospective study | Canadian Medical Association journal | 2002 | Excluded | Analysis |
|  | Saiki et al., | Effect of Spinal Alignment Changes on Lower Back Pain in Patients Treated with Total Hip Arthroplasty for Hip Osteoarthritis | Medicina | 2021 | Excluded | Analysis |
|  | Sasaki et al., | The relationship between ambulatory ability before surgery and the D-dimer value after total hip arthroplasty: The evaluation of ambulatory ability by the timed "Up & Go" test | Acta Medica Okayama | 2005 | Excluded | Analysis |
|  | Çakmak et al., | The effects of modified hardinge approach on hip muscle strength in patients with primary hip arthroplasty: a patient evaluation with isokinetic strength test and gait analyses | European Journal of Orthopaedic Surgery & Traumatology | 2024 | Excluded | Analysis |
|  | Sexton et al., | The role of patient factors and implant position in squeaking of ceramic-on-ceramic total hip replacements | The Journal of Bone & Joint Surgery | 2011 | Excluded | Analysis |
|  | Weng et al., | The effect of total hip arthroplasty on sagittal spinal-pelvic-leg alignment and low back pain in patients with severe hip osteoarthritis | European Spine Journal | 2016 | Excluded | Analysis |
|  | Koutalos et al., | Revision total hip arthroplasty for aseptic loosening compared with primary total hip arthroplasty for osteoarthritis: long-term clinical, functional and quality of life outcome data | Hip International | 2022 | Excluded | Analysis |
|  | Morlock et al., | Duration and frequency of every day activities in total hip patients | Journal of biomechanics | 2001 | Excluded | Analysis |
|  | Nankaku et al., | Factors associated with ambulatory status 6 months after total hip arthroplasty | Physiotherapy | 2014 | Excluded | Analysis |
|  | Fontalis et al., | Functional implant positioning in total hip arthroplasty and the role of robotic-arm assistance | International Orthopaedics | 2023 | Excluded | Analysis |
|  | Ikutomo et al., | Gait Abnormality Predicts Falls in Women After Total Hip Arthroplasty | The Journal of Arthroplasty | 2018 | Excluded | Analysis |
|  | Nozaki et al., | Association of affected and non-affected side ability with postoperative outcomes in patients undergoing total hip arthroplasty | Hip International | 2024 | Excluded | Analysis |
|  | Robbins et al., | Impact of combined lumbar spine fusion and total hip arthroplasty on spine, pelvis, and hip kinematics during a sit to stand task | [Journal of Electromyography and Kinesiology](https://www.sciencedirect.com/journal/journal-of-electromyography-and-kinesiology) | 2024 | Excluded | Analysis |
|  | Zhang et al., | Total Hip Arthroplasty: Leg Length Discrepancy Affects Functional Outcomes and Patient's Gait | Cell biochemistry and biophysics | 2015 | Excluded | Analysis |
|  | Maezawa et al., | Influence of hip joint dysfunction on motor disorders in Japanese patients with osteoarthritis of the hip: Assessment of the JHEQ and GLFS-25 scores and hip muscle strength | Archives of Gerontology and Geriatrics | 2019 | Excluded | Analysis |
|  | Guler et al., | Early improvement in physical activity and function after total hip arthroplasty: Predictors of outcomes | Turkish journal of physical medicine and rehabilitation | 2019 | Excluded | Analysis |
|  | Piazzolla et al., | Spinopelvic parameter changes and low back pain improvement due to femoral neck anteversion in patients with severe unilateral primary hip osteoarthritis undergoing total hip replacement | European Spine Journal | 2018 | Excluded | Analysis |
|  | Jeldi et al., | Total Hip Arthroplasty Improves Pain and Function but Not Physical Activity | The Journal of arthroplasty | 2017 | Excluded | Analysis |
|  | Hjorth et al., | The correlation between activity level, serum-ion concentrations and pseudotumours in patients with metal-on-metal hip articulations and metal-on-polyethylene total hip articulations | Journal of orthopaedic translation | 2019 | Excluded | Analysis |
|  | Schmalzried et al., | Wear is a function of use, not time | Clinical Orthopaedics and Related Research | 2000 | Excluded | Analysis |
|  | Balasubramaniam et al., | Functional and clinical outcomes following anterior hip replacement: a 5-year comparative study versus posterior approach | ANZ Journal of Surgery | 2016 | Excluded | Analysis |
|  | Holm et al., | Role of preoperative pain, muscle function, and activity level in discharge readiness after fast-track hip and knee arthroplasty | Acta orthopaedica | 2014 | Excluded | Analysis |
|  | Bahr et al., | A Retrospective Single-Center Study of 23 Patients to Compare Gait before and after Total Hip Arthroplasty Using the S-ROM Modular Hip System | Medical Science Monitor | 2021 | Excluded | Analysis |
|  | Shimizu et al., | Usefulness of structural equation modeling in evaluation of prognosis of total hip replacement for coxarthrosis | Journal of Physical Therapy Science | 2005 | Excluded | Analysis |
|  | Jungmann et al., | Relationship of unilateral total hip arthroplasty (THA) to contralateral and ipsilateral knee joint degeneration - a longitudinal 3T MRI study from the Osteoarthritis Initiative (OAI) | Osteoarthritis and Cartilage | 2015 | Excluded | Analysis |
|  | Esbjörnsson et al., | Geometrical restoration during total hip arthroplasty is related to change in gait pattern - a study based on computed tomography and three-dimensional gait analysis | BMC Musculoskeletal Disorders | 2021 | Excluded | Analysis |
|  | Al-Amiry et al., | The Influence of Radiological Severity and Symptom Duration of Osteoarthritis on Postoperative Outcome After Total Hip Arthroplasty: A Prospective Cohort Study | The Journal of Arthroplasty | 2018 | Excluded | Analysis |
|  | Silva | Síndrome anca-coluna e o efeito da artroplastia total da anca na dor lombar e no equilíbrio sagital da coluna vertebral: estudo observacional prospetivo (URL: <https://www.proquest.com/pqdtglobal/docview/2925385045/7F5637D80BF2412CPQ/1?accountid=15115&sourcetype=Dissertations%20&%20Theses> ) | ProQuest Dissertations & Theses | 2019 | Excluded | Analysis |
|  | Kennedy et al., | Modeling early recovery of physical function following hip and knee arthroplasty | BMC musculoskeletal disorders | 2006 | Excluded | Analysis |
|  | Parcells et al., | The Direct Anterior Approach for 1-Stage Bilateral Total Hip Arthroplasty: Early Outcome Analysis of a Single-Surgeon Case Series | The Journal of Arthroplasty | 2016 | Excluded | Population and outcome |
|  | Thummala et al., | Does surgery change pelvic tilt? AN INVESTIGATION IN PATIENTS WITH OSTEOARTHRITIS OF THE HIP, DYSPLASIA, AND FEMOROACETABULAR IMPINGEMENT | The bone & joint journal | 2022 | Excluded | Population and outcome |
|  | Slaven | Prediction of functional outcome at six months following total hip arthroplasty | Physical therapy | 2012 | Excluded | Population and outcome |
|  | Jo et al., | Driving Simulator Brake Reaction Parameters After Total Hip Arthroplasty According to Different Surgical Approaches | The Journal of Arthroplasty | 2022 | Excluded | Population and outcome |
|  | Zaballa et al., | Feasibility and sustainability of working in different types of jobs after total hip arthroplasty: analysis of longitudinal data from two cohorts | Occupational and Environmental Medicine | 2022 | Excluded | Population and outcome |
|  | Foucher | Identifying clinically meaningful benchmarks for gait improvement after total hip arthroplasty | Journal of Orthopaedic Research | 2016 | Excluded | Population and outcome |
|  | Dong et al., | Conversion of a Fused or Ankylosed Hip to Total Hip Arthroplasty: Is the Direct Anterior Approach in the Lateral Decubitus Position an Ideal Solution? | Frontiers in Surgery | 2022 | Excluded | Population and outcome |
|  | Fujita et al., | Oxford Hip Scores, Floor-Sitting Score Trajectories, and Postoperative Satisfaction Rates at 10 Years After Primary Total Hip Arthroplasty | The Journal of arthroplasty | 2023 | Excluded | Population and outcome |
|  | Eskildsen et al., | Acetabular Reconstruction With the Medial Protrusio Technique for Complex Primary and Revision Total Hip Arthroplasties | The Journal of Arthroplasty | 2017 | Excluded | Population and outcome |
|  | Inagaki et al., | Natural history of noise and squeaking in cementless ceramic-on-ceramic total hip arthroplasty | Journal of Orthopaedics | 2020 | Excluded | Population and outcome |
|  | Delaunay et al., | Cross-cultural adaptations of the Oxford-12 HIP score to the French speaking population | Orthopaedics & Traumatology: Surgery & Research | 2009 | Excluded | Population and outcome |
|  | Wade et al., | Functional and radiological outcome of uncemented total hip arthroplasty in young adults - 5 year follow-upollow-up | Journal of Orthopaedics | 2020 | Excluded | Population and outcome |
|  | Uesugi et al., | Validity and responsiveness of the Oxford hip score in a prospective study with Japanese total hip arthroplasty patients | Journal of Orthopaedic Science | 2009 | Excluded | Population and outcome |
|  | Gosens et al., | Cementless mallory-head HA-coated hip arthroplasty for osteoarthritis in hip dysplasia | The Journal of arthroplasty | 2003 | Excluded | Population and outcome |
|  | Boyer et al., | Presence and predictors of pain after orthopedic surgery and associated orthopedic outcomes in children with cerebral palsy | Paediatric and Neonatal Pain | 2022 | Excluded | Population and outcome |
|  | Yu et al., | Changes in Alignment of Ipsilateral Knee on Computed Tomography after Total Hip Arthroplasty for Developmental Dysplasia of the Hip | Orthopaedic Surgery | 2019 | Excluded | Population and outcome |
|  | Qoreishy et al., | Evaluation of changes in radiographic spinopelvic parameters after primary total hip arthroplasty and its relationship with short-term patient function | European Journal of Orthopaedic Surgery & Traumatology | 2023 | Excluded | Population and outcome |
|  | Lübbeke et al., | Influence of patient activity on femoral osteolysis at five and ten years following hybrid total hip replacement | The Journal of Bone & Joint Surgery | 2011 | Excluded | Population and outcome |
|  | Rat et al., | Total hip or knee replacement for osteoarthritis: mid- and long-term quality of life | Arthritis Care & Research | 2010 | Excluded | Population and outcome |
|  | Hara et al., | Sports Participation in Patients After Total Hip Arthroplasty vs Periacetabular Osteotomy: A Propensity Score-Matched Asian Cohort Study | The Journal of Arthroplasty | 2018 | Excluded | Population and outcome |
|  | Müller et al., | Relationship between cup position and obturator externus muscle in total hip arthroplasty | Journal of orthopaedic surgery and research | 2010 | Excluded | Population and outcome |
|  | Hamadouche et al., | Total hip arthroplasty for the treatment of ankylosed hips: a five to twenty-one-year follow-up study | The Journal of bone and joint surgery | 2001 | Excluded | Population and outcome |
|  | Sato et al., | Midterm Outcomes and Frequency of Osteolysis of Total Hip Arthroplasty Using Cementless Modular Stem for Asian Patients | The Journal of Arthroplasty | 2020 | Excluded | Population and outcome |
|  | Matsushita et al., | A Modified Modular Stem in Primary Total Hip Arthroplasty for Developmental Dysplasia of the Hip: Average 11-year Follow-Up in Cases With Previously Reported 3-year Clinical Results | The Journal of Arthroplasty | 2022 | Excluded | Population and outcome |
|  | Kirschner et al., | [Reliability, validity and responsiveness of the German short musculoskeletal function assessment questionnaire (SMFA-D) in patients with osteoarthritis of the hip undergoing total hip arthroplasty] | Zeitschrift fur Rheumatologie | 2003 | Excluded | Population and outcome |
|  | Wang et al., | Predicting short-term outcome of primary total hip arthroplasty:a prospective multivariate regression analysis of 12 independent factors | The Journal of arthroplasty | 2010 | Excluded | Population and outcome |
|  | Halket et al., | Using hierarchical linear modeling to explore predictors of pain after total hip and knee arthroplasty as a consequence of osteoarthritis | The Journal of arthroplasty | 2010 | Excluded | Population and outcome |
|  | den Hartog et al., | Which patient-specific and surgical characteristics influence postoperative pain after THA in a fast-track setting? | BMC Musculoskeletal Disorders | 2017 | Excluded | Population and outcome |
|  | Perronne et al., | How is quality of life after total hip replacement related to the reconstructed anatomy? A study with low-dose stereoradiography | Diagnostic and interventional imaging | 2021 | Excluded | Population and outcome |
|  | Brokelman et al., | Patient versus surgeon satisfaction after total hip arthroplasty | Journal of Bone & Joint Surgery | 2003 | Excluded | Population and outcome |
|  | Krayn-Deckel et al., | Cognitive status is associated with performance of manual wheelchair skills in hospitalized older adults | Disability and Rehabilitation: Assistive Technology | 2022 | Excluded | Population and outcome |
|  | Shemesh et al., | Hip arthroplasty with the articular surface replacement (ASR) system: survivorship analysis and functional outcomes | European Journal of Orthopaedic Surgery & Traumatology | 2013 | Excluded | Population and outcome |
|  | Smith et al., | Empirical support for radiographic review: a follow-up study of total hip arthroplasty | Hip International | 2013 | Excluded | Population and outcome |
|  | Tai et al., | Squeaking in large diameter ceramic-on-ceramic bearings in total hip arthroplasty | The Journal of arthroplasty | 2015 | Excluded | Population and outcome |
|  | Aarons et al., | Fatigue after major joint arthroplasty: Relationship to preoperative fatigue and postoperative emotional state | Journal of Psychosomatic Research | 1996 | Excluded | Population and outcome |
|  | Zywiel et al., | Are abductor muscle quality and previous revision surgery predictors of constrained liner failure in hip arthroplasty? | International Orthopaedics | 2011 | Excluded | Population and outcome |
|  | Harada et al., | Squatting After Total Hip Arthroplasty: Patient-Reported Outcomes and In Vivo Three-Dimensional Kinematic Study | The Journal of arthroplasty | 2022 | Excluded | Population and outcome |
|  | Loppini et al., | Femur first surgical technique: a smart noncomputer-based procedure to achieve the combined anteversion in primary total hip arthroplasty | BMC Musculoskeletal Disorders | 2017 | Excluded | Population and outcome |
|  | Ruth Ann Kiefer | The Effect of Social Support on Functional Recovery and Well-Being in Older Adults Following Joint Arthroplasty | Rehabilitation Nursing Journal | 2011 | Excluded | Population and outcome |
|  | Fessy et al., | Midterm Clinical and Radiographic Outcomes of a Contemporary Monoblock Dual-Mobility Cup in Uncemented Total Hip Arthroplasty | The Journal of Arthroplasty | 2019 | Excluded | Population and outcome |
|  | Wang et al., | The Direct Anterior Approach versus the Posterolateral Approach on the Outcome of Total Hip Arthroplasty: A Retrospective Clinical Study | Orthopaedic Surgery | 2022 | Excluded | Population and outcome |
|  | de Groot et al., | Small increase of actual physical activity 6 months after total hip or knee arthroplasty | Clinical orthopaedics and related research | 2008 | Excluded | Population and analysis |
|  | Motififard et al., | Outcomes of Unilateral Total Hip Arthroplasty in Patients Aged Under 35 Years in Iranian Population: A Preliminary Study | Advanced Biomedical Research | 2018 | Excluded | Population and analysis |
|  | Kiyama et al., | Hip Abductor Strengths After Total Hip Arthroplasty Via the Lateral and Posterolateral Approaches | The Journal of arthroplasty | 2010 | Excluded | Population and analysis |
|  | Bahl et al., | Lower functioning patients demonstrate atypical hip joint loading before and following total hip arthroplasty for osteoarthritis | Journal of Orthopaedic Research | 2020 | Excluded | Population and analysis |
|  | Kennedy et al., | Preoperative function and gender predict pattern of functional recovery after hip and knee arthroplasty | The Journal of arthroplasty | 2006 | Excluded | Population and analysis |
|  | Kalisch et al., | Everyday physical activity and sedentary behavior after total joint arthroplasty: Do patients and partners develop an active lifestyle? | Clinical Interventions in Aging | 2021 | Excluded | Population and analysis |
|  | Issa et al., | Patient satisfaction after total hip arthroplasty in an Egyptian population as an example of a developing country | Current Orthopaedic Practice | 2016 | Excluded | Population and analysis |
|  | Innmann et al., | How Can Patients With Mobile Hips and Stiff Lumbar Spines Be Identified Prior to Total Hip Arthroplasty? A Prospective, Diagnostic Cohort Study | The Journal of Arthroplasty | 2020 | Excluded | Population and analysis |
|  | Okuzu et al., | Investigating sagittal spinal alignment, low back pain, and clinical outcomes after total hip arthroplasty for lumbar hyperlordosis: a retrospective study | Archives of Orthopaedic and Trauma Surgery | 2022 | Excluded | Population and analysis |
|  | Łaziński et al., | An Analysis of the Preoperative Factors, Spinopelvic Mobility and Sagittal Spinal Alignment in Pre-THA Patients | Journal of Clinical Medicine | 2023 | Excluded | Population and analysis |
|  | Breuer et al., | Prospective short-term and return-to-sports results of a novel uncemented short-stem hip prosthesis with metaphyseal anchorage | Journal of Clinical Medicine | 2020 | Excluded | Population and analysis |
|  | McDonnell et al., | The incidence of noise generation arising from the large-diameter Delta Motion ceramic total hip bearing | The bone & joint journal | 2013 | Excluded | Population and analysis |
|  | von Bernstorff et al., | Evaluation of braking performances of patients with osteoarthritis of the knee or hip: Are there alternatives to a brake simulator? | Acta orthopaedica et traumatologica turcica | 2021 | Excluded | Population and analysis |
|  | Berton et al., | The Durom Large Diameter Head acetabular component: EARLY RESULTS WITH A LARGE-DIAMETER METAL-ON-METAL BEARING | Journal of Bone & Joint Surgery | 2010 | Excluded | Population and analysis |
|  | Ge et al., | Relationship between low back pain and spinal-pelvic sagittal parameter changes in patients with hip-spine syndrome after total hip arthroplasty | Chinese Journal of Tissue Engineering Research | 2024 | Excluded | Population and analysis |
|  | Innmann et al., | How Does Spinopelvic Mobility and Sagittal Functional Cup Orientation Affect Patient-Reported Outcome 1 Year after THA?-A Prospective Diagnostic Cohort Study | The Journal of Arthroplasty | 2021 | Excluded | Population and analysis |
|  | Wu et al., | Cementless large-head metal-on-metal total hip arthroplasty in patients younger than 60 years - A multicenter early result | The Kaohsiung Journal of Medical Sciences | 2012 | Excluded | Population and analysis |
|  | Noor et al., | Analysis on association between sagittal stem alignment and early functional and radiological outcome following primary cementless total hip replacement | European Journal of Orthopaedic Surgery & Traumatology | 2024 | Excluded | Population and analysis |
|  | Welters et al., | One-stage bilateral total hip replacement: A retrospective study of 70 patients | Acta Orthopaedica Belgica | 2002 | Excluded | Population and analysis |
|  | Zhang et al., | Clinical and radiologic outcomes in patients undergoing primary total hip arthroplasty with Collum Femoris Preserving stems: a comparison between the direct anterior approach and the posterior approach | BMC Musculoskeletal Disorders | 2022 | Excluded | Population and analysis |
|  | Takenaga et al., | Which functional assessments predict long-term wear after total hip arthroplasty? | Clinical Orthopaedics and Related Research | 2013 | Excluded | Population and analysis |
|  | Okamoto et al., | Association of the Psoas Muscle Index and Sagittal Spinal Alignment With Patient-Reported Outcomes After Total Hip Arthroplasty: A Minimum 5-Year Follow-Up | The Journal of Arthroplasty | 2022 | Excluded | Population and analysis |
|  | Valdivia-Zúñiga et al., | [Frequency of spinopelvic alterations in postoperative total hip arthroplasty patients and their association with functional outcomes] | Acta Ortopédica Mexicana | 2024 | Excluded | Population and analysis |
|  | Okuzu et al., | How Do Spinal Parameters Change in Patients Who Have Improvement of Low Back Pain After Total Hip Arthroplasty? A Propensity Score-Matched Cohort Study | The Journal of Arthroplasty | 2024 | Excluded | Population and analysis |
|  | Okamoto et al., | Association of global sagittal spinal deformity with functional disability two years after total hip arthroplasty | BMC Musculoskeletal Disorders | 2021 | Excluded | Population and analysis |
|  | Ilchmann et al., | Poor outcome of a spherical pressfit cup with a modern ceramic liner: a prospective cohort study of 181 cups | Hip International | 2014 | Excluded | Population and analysis |
|  | Thewlis et al., | Objectively measured 24-hour activity profiles before and after total hip arthroplasty | The Bone & Joint Journal | 2019 | Excluded | Population and analysis |
|  | Lau et al., | 3rd generation ceramic-on-ceramic cementless total hip arthroplasty: a minimum 10-year follow-up study | HIP International | 2018 | Excluded | Population, outcome, and analysis |
|  | Dessyn et al., | A 20-year follow-up evaluation of total hip arthroplasty in patients younger than 50 using a custom cementless stem | HIP International | 2019 | Excluded | Population, outcome, and analysis |
|  | Gruber et al., | Functional and radiological outcomes after treatment with custom-made acetabular components in patients with Paprosky type 3 acetabular defects: short-term results | BMC Musculoskeletal Disorders | 2020 | Excluded | Population, outcome, and analysis |
|  | Chevillotte et al., | Hip Squeaking. A 10-Year Follow-Up Study | The Journal of Arthroplasty | 2012 | Excluded | Population, outcome, and analysis |
|  | Wyles et al., | Total Hip Arthroplasty Reduces Pain and Improves Function in Patients With Spondyloepiphyseal Dysplasia: A Long-Term Outcome Study of 50 Cases | The Journal of arthroplasty | 2019 | Excluded | Population, outcome, and analysis |
|  | Koper et al., | A 5-year survival analysis of 160 Biomet Magnum M2 metal-on-metal total hip prostheses | Hip International | 2016 | Excluded | Population, outcome, and analysis |
|  | Mervinrosario et al., | CT Based Study of Version of the Acetabular Cup after Total Hip Arthroplasty and its Correlation with the Functional Outcome | Journal of Research in Medical and Dental Science | 2021 | Excluded | Population, outcome, and analysis |
|  | Grammatopoulos et al., | 2018 Frank Stinchfield Award: Spinopelvic Hypermobility Is Associated With an Inferior Outcome After THA: Examining the Effect of Spinal Arthrodesis | Clinical Orthopaedics and Related Research | 2019 | Excluded | Population, outcome, and analysis |
|  | Kushwaha et al., | Functional outcome of total hip arthroplasty in tubercular hip arthritis: A prospective study | Journal of Clinical Orthopaedics and Trauma | 2024 | Excluded | Population, outcome, and analysis |
|  | Kuo et al., | Ceramic-on-ceramic total hip arthroplasty: incidence and risk factors of bearing surface-related noises in 125 patients | Orthopedics | 2012 | Excluded | Population, outcome, and analysis |
|  | Wiesman et al., | Total hip replacement with and without osteotomy of the greater trochanter. Clinical and biomechanical comparisons in the same patients | The Journal of Bone and Joint Surgery | 1978 | Excluded | Population, outcome, and analysis |
|  | Bernasek et al., | Is metal-on-metal squeaking related to acetabular angle of inclination? | Clinical Orthopaedics and Related Research | 2011 | Excluded | Population, outcome, and analysis |
|  | Trisolino et al., | Cementless Ceramic-on-Ceramic Total Hip Replacement in Children and Adolescents | Children | 2021 | Excluded | Population, outcome, and analysis |
|  | Klit et al., | Alternative outcome measures in young total hip arthroplasty patients: a prospective cohort study | Hip International | 2015 | Excluded | Outcome and analysis |
|  | Yildiz et al., | [Evaluation of unilateral total hip arthroplasty by a quantitative analysis of gait] | Acta Orthopaedica et Traumatologica Turcica | 2002 | Excluded | Outcome and analysis |
|  | Leonard et al., | Direct superior approach for total hip arthroplasty | The Bone & Joint Journal | 2021 | Excluded | Outcome and analysis |
|  | Zandi et al., | Spinopelvic imbalances are associated with worse postoperative functional outcomes in patients undergoing total hip arthroplasty | European Journal of Orthopaedic Surgery & Traumatology | 2023 | Excluded | Outcome and analysis |
|  | Kijima et al., | Relationship between clinical results of total hip replacement and postoperative activities including weeding and snow shovelling | Hip International | 2018 | Excluded | Outcome and analysis |
|  | Ricketts et al., | A single femoral component for all total hip replacements performed by a trust? Does this affect early clinical and radiological outcomes? | Archives of Bone and Joint Surgery | 2018 | Excluded | Outcome and analysis |
|  | Kechagias et al., | Hip-Spine and Knee-Spine Syndrome: Is Low Back Pain Improved After Total Hip and Knee Arthroplasty? | Cureus | 2024 | Excluded | Outcome and analysis |
|  | Johansson et al., | Impact of preoperative function on early postoperative outcome after total hip arthroplasty | Journal of orthopaedic surgery | 2010 | Excluded | Outcome and analysis |
|  | Stevens et al., | Physical Activity Participation Among Patients After Total Hip and Knee Arthroplasty | Clinics in geriatric medicine | 2012 | Excluded | Design |
|  | Banke et al., | [Gluteal insufficiency] | Der Orthopäde | 2020 | Excluded | Design |
|  | Behery et al., | Are Harris Hip Scores and Gait Mechanics Related Before and After THA? | Clinical Orthopaedics and Related Research | 2014 | Excluded | Design |
|  | Brunner et al., | Sex specific associations between biomechanical recovery and clinical recovery after total hip arthroplasty | Clinical Biomechanics | 2018 | Excluded | Design |
|  | Stratford et al., | Quantifying Self-Report Measures’ Overestimation of Mobility Scores Postarthroplasty | Physical therapy | 2010 | Excluded | Design |
|  | Mattsson et al., | Walking efficiency after cemented and noncemented total hip arthroplasty | Clinical Orthopaedics and Related Research | 1990 | Excluded | Design |
|  | Foucher | Preoperative gait mechanics predict clinical response to total hip arthroplasty | Journal of Orthopaedic Research | 2017 | Excluded | Design and analysis |
|  | Müller et al., | MRI findings of gluteus minimus muscle damage in primary total hip arthroplasty and the influence on clinical outcome | Archives of orthopaedic and trauma surgery | 2010 | Excluded | Design and analysis |
|  | Jensen et al., | Quantifying Gait Quality in Patients with Large-Head and Conventional Total Hip Arthroplasty--A Prospective Cohort Study | The Journal of arthroplasty | 2015 | Excluded | Design and population |
|  | Gandhi et al., | Relationship between self-reported and performance-based tests in a hip and knee joint replacement population | Clinical rheumatology | 2009 | Excluded | Design and population |
|  | Leigheb et al., | Sarcopenia diagnosis: Reliability of the ultrasound assessment of the tibialis anterior muscle as an alternative evaluation tool | Diagnostics | 2021 | Excluded | Population, intervention, and analysis |
|  | Lenguerrand et al., | Pain and Function Recovery Trajectories following Revision Hip Arthroplasty: Short-Term Changes and Comparison with Primary Hip Arthroplasty in the ADAPT Cohort Study | PLoS One | 2016 | Excluded | Population, intervention, and analysis |
|  | Poitras et al., | Assessing functional recovery shortly after knee or hip arthroplasty: a comparison of the clinimetric properties of four tools | BMC musculoskeletal disorders | 2016 | Excluded | Intervention |
|  | Stavrakis et al., | Dual Mobility Total Hip Arthroplasty Is Not Associated with a Greater Incidence of Groin Pain in Comparison with Conventional Total Hip Arthroplasty and Hip Resurfacing:A Retrospective Comparative Study | HSS Journal | 2020 | Excluded | Intervention and outcome |
|  | Lavigne et al., | Range of motion of large head total hip arthroplasty is greater than 28 mm total hip arthroplasty or hip resurfacing | Clinical biomechanics | 2011 | Excluded | Intervention and population |
|  | Brown et al., | Evaluation of Differences Between Non-Hispanic White and African American Patients With Sports Medicine–Related Hip Disabilities | Orthopaedic Journal of Sports Medicine | 2022 | Excluded | Population, intervention, and outcome |
|  | Sharma et al., | Functional Outcome before and after Total Hip Arthroplasty | Indian Journal of Physiotherapy & Occupational Therapy | 2014 | Excluded | Design, outcome, and analysis |
