# Supplementary material for: Association between physical measures of spinopelvic alignment and physical functioning with patient reported outcome measures after total hip arthroplasty: Systematic review and narrative synthesis
Source: PLoS One. 2025 Dec 29;20(12):e0339615. doi: 10.1371/journal.pone.0339615 (PMC12747333; doi:10.1371/journal.pone.0339615)
Supplement: S5 Appendix — (DOCX) [file pone.0339615.s005.docx]

**Study characteristics and results**

| **Number** | **Eligible to be included in the review** | **Data extractors and**  **date of extraction** | **Study characteristics** | **Study objectives** | **Participant Characteristics** | **Physical outcome measures (Equipment)** | **PROMs** | **Timepoints** | **Statistical analysis and results** |
| --- | --- | --- | --- | --- | --- | --- | --- | --- | --- |
|  | Yes | SV and LF  February/March 2024  SV and LF  February/March 2024 | 1. Luna et al., (2017) [1] Denmark (English) Study design: Prospective cohort longitudinal Funding: A grant from the Lundbeck Foundation, Hellerup Denmark | 1. To assess early physical function after THA and the correlation between patient-reported outcome measures, physical performance and actual physical activity (measured by actigraphy) | 1. Age: 67 (NR: SD) N= 40 F (25), M (15)  THA approach: NR Type: Unilateral LBP: NR Health conditions: NR | 1. Performance-based: 40 m FPWT CST SCT  1. Activity level in natural environment: Actual physical activity (Actiwatch) | 1. Physical function: HOOS ADL | 1. PROMs Preop: 14 to 3 days Postop: 13 days 1. Performance-based: Preop: 14 to 3 days Postop: 14 days 1. Actual physical activity | 1. Longitudinal analysis from preoperatively to postoperatively (SCC): Change in 40 m FPWT, CST, and SCT were not associated with change in physical function (HOOS ADL, r²= 0.16, p= 0.31, NR: rₛ, p value, and CI for each associations). Change in actual physical activity was not associated with change in physical function (HOOS ADL, r²= 0.10, p= 0.54, NR: rₛ and CI). |
|  |  |  | 2. Luna et al., (2019) [2] Denmark (English) Study design: Prospective cohort longitudinal Funding: The Lundbeck Foundation, Hellerup, Denmark, supported the study with a Grant (No: R25-A2702) | 2. To describe early post-THA physical activity measured by actigraphy and potential underlying pathophysiological mechanisms related to recovery in a well-defined cohort of THA patients | 2. Age: 67 (NR: SD) N= 40 F (25), M (15)  THA approach: NR  Type: Unilateral LBP: NR  Health conditions: NR | 2. Activity level in natural environment: Actual physical activity (Actiwatch) | 2. Pain: The scale or questionnaire was not reported | Preop: 2 days Postop: 12 and 13 days 2. PROMs Preop: 2 days Postop: 14 days 2. Actual physical activity: Preop: 2 days Postop: 13 days | 2. Longitudinal analysis from preoperatively to postoperatively (Backwards multiple regression): Total activity trajectory was not associated with worse pain and average pain (NR: The scale or questionnaire, β, p value, and CI for all associations). High intense physical activity was not associated with worse pain and average pain (NR: The scale or questionnaire, β, p value, and CI for all associations). |
|  | Yes | SV and RR  February/March 2024 | 1. Heiberg et al., (2013) [3]  Norway (English) Study design: Prospective cohort longitudinal Funding: The South−Eastern Norway Regional Health Authority  2. Heiberg (2013) [4]  Norway (English) Study design: Prospective cohort longitudinal Funding: NR | To examine which preoperative measures of physical functioning could predict walking distance outcomes after THA | Age (Population completed assessment at 3 months) = 66 (NR: SD) N (Population completed assessment at 3 months) = 88 F (51), M (37)  THA approach: Posterolateral  Type: Unilateral LBP: NR  Health conditions: Heart attack, angina, diabetes, cancer, osteoporosis, musculoskeletal disorders, stomach/intestinal problems, lung disease, psychological disorder | Performance-based: 6MWT | Pain and function: HHS pain and function HOOS sport/recreation  HOOS ADL  HOOS pain | Preop Postop: 3 months  (Data extraction was limited to the 3-month follow-up because the dissertation[4] indicated that the study design was observational over this period.) | Longitudinal analyses from preoperative to postoperative (PCC: Bivariate analysis): Postoperative 6MWT could be predicted by preoperative pain and function (HHS pain and function (r= 0.24, p < 0.05, NR: CI), HOOS sport/recreation (r= 0.33, p < 0.01, NR: CI), and HOOS ADL (r= 0.29, p < 0.01, NR: CI)), but it could not be predicted by preoperative pain (HOOS pain, r= 0.15, p > 0.05, NR: CI).  Longitudinal analyses rom preoperative to postoperative (Multiple regression analyses): Postoperative 6MWT could not be predicted by preoperative pain and function (HHS pain and function (Crude ß= -1.0, CIs= -2.2 to 0.3, p= 0.11) and HOOS sport/recreation (Crude ß= 0.6, CIs= -0.2 to 1.4, p= 0.15)). |
|  | Yes | SV and LF  February/March 2024 | Abujaber (2014) [5] USA (English) Study design: Cross-sectional and prospective cohort longitudinal  Funding: The University of Jordan, the National Institutes of Health (K12 HD055931), Comprehensive Opportunities in Rehabilitation Research Training (CORRT), and the University of Delaware Research Foundation | To identify how physical impairments are related to the patient’s perceptions after THA | Age: 65.3 ± 8.4 N= 48 F (20), M (28)  THA approach: Anterolateral or posterior  Type: Unilateral LBP: NR Health conditions: NR | Performance-based: TUG SCT 6MW  Impairment-based: Hip ROM (Goniometer) Hip abductor strength  knee extensor strength  (Dynamometer) | Hip pain: A continuous scale from 0 to 10  Hip function: Hip Outcome Score-Activities of Daily Living Subscale (HOS) | Preop: 2-4 weeks Postop: 3 months | Cross-sectional analyses postoperatively (PCC): TUG (r= -0.08, p > 0.05), SCT (r= -0.007, p > 0.05), and 6MWT (r= 0.05, p > 0.05) were not associated with hip pain (A continuous scale from 0 to 10, NR: CI for all associations).  Greater hip ROM was associated with better function (HOS, r= 0.34, p= 0.01), but hip abductor strength (r= 0.18, p > 0.05) and knee extensor strength (r= 0.16, p > 0.05) were not associated with function (HOS, NR: CI for all associations).  Cross-sectional analyses postoperatively (Hierarchical linear regressions): Improvement in TUG (R² Change= 0.00, p-value change= 0.96), SCT (R² Change= 0.007, p-value change= 0.54), and 6MW (R² Change= 0.00, p-value change= 0.97) could not be predicted by change in hip pain (A continuous scale from 0 to 10) after accounting the effect of age and BMI.  Longitudinal analyses from preoperatively to postoperatively (PCC): Greater change in TUG (r= 0.28, p= 0.05), SCT (r= 0.29, p= 0.05), and 6MWT (r= -0.40, p= 0.01) were associated with greater change in hip pain (A continuous scale from 0 to 10, NR: CI for all associations).  Greater change in hip ROM (r= 0.34, p= 0.01) and knee extensor strength (r= 0.37, p= 0.01) were associated with greater change in function (HOS), but change in hip abductor strength was not associated with change in function (HOS, r= 0.08, p > 0.05) (NR: CI for all associations).   Longitudinal analyses from preoperatively to postoperatively (Hierarchical linear regressions): Improvement in TUG (R² Change= 0.09, p-value change= 0.04), SCT (R² Change= 0.10, p-value change= 0.03), and 6MW (R² Change= 0.15, p-value change= 0.009) could be predicted by change in hip pain (A continuous scale from 0 to 10) after accounting the effect of age and BMI. |
|  | Yes | SV and RR  February/March 2024 | Biggs et al., (2022) [6] Sweden (English) Study design: Prospective cohort longitudinal Funding: Stiftelsen Promobilia, Grant/Award Number: 17111; Stiftelsen Skobranschens utvecklingsfond; Swedish Rheumatic Association, Grant/Award Number: R‐754381 | Primary:(1) To quantify and summarize overall gait function using the Cardiff Classifier, (2) evaluate whether this comprehensive gait  measure can predict improvements in postoperative patient‐reported function and overall gait function 1 year after THA. Secondary, to explore potential relationships between change (pre vs. post THA) in overall gait function versus changes in patient‐reported and performance‐based function | Age: 66.6 ± 9.2 N= 32 F (23), M (9)  THA approach: Direct lateral  Type: Unilateral LBP: No (Excluded severe back pain) Health conditions: NR | Impairment-based: Gait function (Vicon Motion Systems) | Pain: HOOS pain   Function: HOOS ADL  HOOS symptoms  HOOS sport and recreation  HOOS hip‐related quality of life | Preop: 1 month Postop: 12 months | Longitudinal analyses from preoperative to postoperative (Multivariable linear regressions): Preoperative gait function can predict change in function (HOOS ADL, unstandardized β= 52.45, CIs= 8.5 to 96.4, standardized β= 0.37, p= 0.02).  Longitudinal analyses from preoperative to postoperative (PCC): Greater change in gait function was associated with lower change in pain (HOOS pain, r= -0.64, p= 0.00) and lower change in function (HOOS ADL (r= -0.60, p= 0.00), HOOS symptoms (r= -0.69, p= 0.00), HOOS sport and recreation (r= -0.49, p= 0.005), HOOS hip‐related quality of life (r= -0.52, p= 0.002)) (NR: CI for all associations). |
|  | Yes | SV and RR  February/March 2024 | Boardman et al., (2000) [7] USA (English) Study design: Cross-sectional and cohort longitudinal Funding: None | To define the association between data derived preoperatively from the SF-36 and WOMAC and understand better how these surveys measure the functional outcome after THA | Age: 65 (NR: SD) N= 30 F (15), M (15)  THA approach: NR Type: Unilateral LBP: NR Health conditions: NR | Performance-based: 6MWT  Impairment-based: Gait velocity Gait symmetry (Stride Analyzer) | Physical function:  SF-36 physical component summary  WOMAC function WOMAC stiffness | Preop Postop: 12 months | Cross-sectional analysis postoperatively (PCC):  Better performance in 6MWT was associated with better physical function (SF-36 physical component summary (r= 0.69) and WOMAC function (r= 0.64)) (NR: p value and CI). Greater gait velocity was associated with better physical function (SF-36 physical component summary (r= 0.81) and WOMAC function (r= 0.67)) (NR: p value and CI). Greater gait symmetry was associated with better physical function (SF-36 physical component summary (r=0.57) and WOMAC function (r=0.50)) (NR: p value and CI).  Longitudinal analyses rom preoperative to postoperative (PCC):  Improvement in 6MWT was associated with improvement in physical function (SF-36 physical component summary (r= 0.58), WOMAC function (r= 0.54), and WOMAC stiffness (r= 0.57)) (NR: p value and CI for all associations). Improvement in gait velocity was associated with improvement in physical function (SF-36 physical component summary (r= 0.63), WOMAC function (r= 0.65), and WOMAC stiffness (r= 0.58)) (NR: p value and CI for all associations). Improvement in gait symmetry was not associated with improvement in physical function (SF-36 physical component summary, WOMAC function, and WOMAC stiffness (NR: r, p value, and CI for all associations)). |
|  | Yes | SV and LF  February/March 2024 | Bolink et al., (2016) [8] United Kingdom (English) Study design: Cross-sectional Funding: The National Institute for Health Research (NIHR) under its Programme Grants for Applied Research programme (RP-PG-0407-10070) | To compare the trajectories of post-operative recovery between the WOMAC function score and gait parameters | Age: 63.9 ± 9.8 N= 36 F (18), M (18)  THA approach: NR  Type: NR LBP: NR Health conditions: NR Diagnosis was reported as hip OA by the study author (February/March 2024). | Impairment-based: Walking speed Cadence Step time Step length Pelvic ROM Step time irregularity Step time asymmetry (3D inertial sensor, gyroscopes, and accelerometers) | Function: WOMAC function | Preop: 24 days Postop: 3 and 12 months | Cross-sectional analyses 3 months postoperatively (SCC): Walking speed (rₛ= 0.31, p= 0.07), cadence (rₛ= 0.24, p= 0.15), step time (rₛ= -0.24, p= 0.15), step length (rₛ= 0.25, p= 0.13), pelvic ROM (rₛ= 0.14, p= 0.42), step time irregularity (rₛ= -0.26, p= 0.11), and step time asymmetry (rₛ= 0.06, p= 0.74) were not associated with function (WOMAC function, NR: CI for all associations).  Cross-sectional analyses 12 months postoperatively (SCC): Faster walking speed (rₛ= 0.45, p= 0.005), greater cadence (rₛ= 0.37, p= 0.02), lower step time (rₛ= -0.37, p= 0.02), greater step length (rₛ= 0.32, p= 0.05), greater pelvic ROM (rₛ= 0.51, p= 0.002), lower step time irregularity (rₛ= -0.39, p= 0.01), and lower step time asymmetry (rₛ= -0.33, p= 0.04) were associated with better function (WOMAC function, NR: CI for all associations). |
|  | Yes | SV and RR  February/March 2024 | Cao et al., (2022) [9] China (English) Study design: Cohort longitudinal Funding: None | To analyze the anatomical changes in the lumbosacral spine and their correlation with facet joint−derived LBP in patients with hip OA after THA | Age: 68 ± 2.45 N= 74 F (35), M (39)  THA approach: Lateral  Type: Unilateral LBP: Yes (Facet-derived LBP) Health conditions: NR | Spinopelvic Alignment: Lumbar lordosis Slip angle Spino−sacral angle (X−ray) | LBP: VAS  Hip pain:  VAS | Preop Postop:  6.24 months | Longitudinal analyses (PCC): Greater lumbar lordosis was associated with lower LBP severity (VAS, p= 0.009) and lower hip pain severity (VAS, p= 0.03) (NR: r and CI for all associations)  Slip angle and spino−sacral angle was not associated with the severity of LBP or hip pain (VAS, p > 0.05) (NR: r and CI for all associations). |
|  | Yes | SV and LF  February/March 2024 | Casartelli et al., (2015) [10] Switzerland (English) Study design: Retrospective cross-sectional Funding: The Schulthess Clinic Research Fund (internal funding) | To evaluate the construct validity of the Physical Activity Scale for the Elderly (PASE) questionnaire relative to accelerometry-based physical activity in patients at 2 different time points in the first year following THA (early (2–7 months) and late (7–12 months) postoperative) | Age (Whole population): 68.3 ± 5.9 N (Whole population)= 50 F (25), M (25)  Age (THA early group): 67.8 ± 6 N (THA early group)= 25 F (13), M (12)  Age (THA late group): 68.9 ± 5.8 N (THA late group)= 25 F (12), M (13)  THA approach: NR Type: Unilateral  LBP: NR  Health conditions: NR Diagnosis was reported as hip OA by the study author (February/March 2024). | Activity level in natural environment: Light intensity activity Moderate- to high-intensity activity Total physical activity (ActiGraph accelerometer) | Physical activity: PASE Questionnaire | Postop in whole population: 7.4 months Postop in THA early group: 4.5 months Postop in THA late group: 10.3 months | Cross-sectional analysis postoperatively (PCC):  In whole population, light intensity activity (r= 0.38, CIs= 0.12 to 0.60, NR: p value), moderate- to high-intensity activity (r= 0.26, CIs= -0.02 to 0.50, NR: p value), and total physical activity (r= 0.27, CIs= 0.00 to 0.51, p= 0.03) were not associated with physical activity (PASE, a correlation coefficient ≥ 0.50 was considered as adequate for physical activity comparisons between questionnaires and accelerometry according to recommendations for physical activity questionnaire). In THA early group, light intensity activity (r= 0.16, CIs= -0.25 to 0.52, p= 0.03), moderate- to high-intensity activity (r= 0.24, CIs= -0.17 to 0.58, NR: p value), and total physical activity (r= 0.14, CIs= -0.27 to 0.51, p= 0.02) were not associated with physical activity (PASE). In THA late group, light intensity activity (r= 0.55, CIs= 0.20 to 0.78, NR: p value), moderate- to high-intensity activity (r= 0.28, CIs= -0.13 to 0.61, NR: p value) and total physical activity (r= 0.36, CIs= -0.04 to 0.66, NR: p value) were not associated with physical activity (PASE). |
|  | Yes | SV and RR  February/March 2024 | Cinnamon et al., (2019) [11] USA (English) Study design: Cross−sectional Funding: A grant from the University of Illinois at Chicago Campus Research Board Pilot Research program (KCF) | To evaluate the relationships between abductor function and physical performance and self−reported function in community dwelling adults 1–5 years post THA | Age: 57 ± 8 N= 18 F (12), M (6) THA approach: NR Type: NR LBP: NR Health conditions: NR Diagnosis was reported as hip OA by the study author (February/March 2024). | Impairment-based: Peak isometric hip abductor strength of the operated side (Dynamometer) Peak hip external adduction moment  (Force plate and motion analysis) | Function: HOOS Function  PROMIS Physical Function | Preop Postop: 25 months | Cross−sectional analyses postoperatively (Linear regression analyses): Greater peak isometric hip abductor strength of the operated side was associated with better function (HOOS function, R= 0.71, p= 0.002, NR: CI), but peak hip external adduction moment was not associated with function (HOOS function, R= 0.49, p= 0.05, NR: CI).  Greater peak isometric hip abductor strength of the operated side was associated with better function (PROMIS physical function, R= 0.61, p= 0.01, NR: CI), but peak hip external adduction moment was not associated with function (PROMIS physical function, R= 0.46, p= 0.07, NR: CI)  Greater peak isometric hip abductor strength of the operated side was associated with better function (HOOS function, B= 40.70, CIs= 11.55 to 69.84, standardized β= 0.62, p= 0.01), but peak hip external adduction moment was not associated with function (HOOS function, B= 4.51, CIs= -2.51 to 11.53, standardized β= 0.28, p= 0.18). Greater peak isometric hip abductor strength of the operated side was associated with better function (PROMIS physical function, B= 12.49, CIs= -0.15 to 25.12, standardized β= 0.51, p= 0.05). However, peak hip external adduction moment was not associated with function (PROMIS physical function, B= 1.74, CIs= -1.31 to 4.78, standardized β= 0.29, p= 0.23). |
|  | Yes | SV and LF  February/March 2024 | Davis et al., (2007) [12]  USA(English)  Study design: Retrospective cross-sectional  Funding: NR | To investigate whether there was a correlation between hip  motion and patient function after THA | Age: 69.0 ± 6.1  N= 1383  F (801), M (582)  THA approach: Posterior (1246) and anterolateral (271)  Type: Unilateral (1249) and bilateral (268)  LBP: NR  Health conditions: NR | Impairment-based:  Hip flexion ROM  Hip abduction ROM  Hip adduction ROM  Hip external rotation ROM  Hip internal rotation ROM  Hip flexion contracture  Hip motion: motion  range combination  (Goniometer) | Function:  HHS (pain and function)  HHS stairs  HHS socks and shoes  HHS sit  HHS limb  HHS walk  HHS pain  High hip function (HHS: highest scores for all of the subscales)  Poor hip function (HHS: lowest scores in any one subscale or more) | Postop:  2,3,5, and 7 years | Cross-sectional analyses postoperatively (SCC):  Greater hip flexion ROM was associated with better function ((HHS, rₛ= 0.27, p < 0.02), (HHS stairs, rₛ= 0.32, p < 0.02), (HHS socks and shoes, rₛ= 0.33, p < 0.02), (HHS limp, rₛ= 0.08, p < 0.02), (HHS walk, rₛ= 0.26, p < 0.02), and (HHS pain, rₛ= 0.08, p < 0.02)), but it was not associated with function (HHS sit, rₛ= -0.03, p > 0.02) (NR: CI for all associations).  Greater hip abduction ROM was associated with worse function ((HHS stairs, rₛ= -0.10, p < 0.02) and (HHS socks and shoes, rₛ= -0.07, p < 0.02)), but it was not associated with function ((HHS, rₛ= -0.02, p > 0.02), (HHS sit, rₛ= 0.02, p > 0.02), (HHS limp, rₛ= -0.02, p > 0.02), (HHS walk, rₛ= -0.01, p > 0.02), (HHS pain, rₛ= -0.05, p > 0.02)) (NR: CI for all associations).  Greater hip adduction ROM was associated with worse function ((HHS stairs, rₛ= -0.11, p < 0.02) and (HHS walk, rₛ= -0.07, p < 0.02)), but it was not associated with function ((HHS, rₛ= 0, p > 0.02), (HHS socks and shoes, rₛ= 0.04, p > 0.02), (HHS sit, rₛ= -0.03, p > 0.02), (HHS limp, rₛ= 0.03, p > 0.02), and (HHS pain, rₛ= -0.03, p > 0.02)) (NR: CI for all associations).  Greater hip external rotation ROM was associated with better function ((HHS socks and shoes, rₛ= 0.14, p < 0.02), (HHS limp, rₛ= 0.13, p < 0.02), (HHS pain, rₛ= 0.06, p < 0.02)), but it was not associated with function ((HHS, rₛ= 0.05, p > 0.02), (HHS stairs, rₛ= 0, p > 0.02), (HHS sit, rₛ= -0.05, p > 0.02), and (HHS walk, rₛ= -0.04, p > 0.02)) (NR: CI for all associations).  Greater hip internal rotation ROM was associated with worse function ((HHS stairs, rₛ= -0.14, p < 0.02) and (HHS walk, rₛ= -0.09, p < 0.02)), but it was not associated with function ((HHS, rₛ= -0.05, p > 0.02), (HHS socks and shoes, rₛ=0.03, p > 0.02), (HHS sit, rₛ= 0, p > 0.02), (HHS limp, rₛ= 0.05, p > 0.02), and (HHS pain, rₛ= -0.06, p > 0.02)) (NR: CI for all associations).  Greater hip flexion contracture was associated with better function (HHS pain, rₛ= 0.06, p < 0.02), but it was not associated with function ((HHS, rₛ= -0.05, p > 0.02), (HHS stairs, rₛ= -0.05, p > 0.02), (HHS socks and shoes, rₛ= -0.05, p > 0.02), (HHS sit, rₛ= -0.02, p > 0.02), (HHS limp, rₛ= -0.03, p > 0.02), and (HHS walk, rₛ= -0.02, p > 0.02)) (NR: CI for all associations).  Cross-sectional analyses postoperatively (Forward selection regression):  Hip flexion ROM (p < 0.0001), hip abduction ROM (p= 0.02), hip adduction ROM (p= 0.01), and hip external rotation ROM (p < 0.0001), was associated with high hip function (HHS: highest scores for all of the subscales), but hip internal rotation ROM (p= 0.64) and hip flexion contracture (p= 0.20) and was not associated with high hip function (HHS: highest scores for all of the subscales) (NR: β and CI for all associations).  Hip flexion ROM (p= 0.005), hip abduction ROM (p= 0.01), and hip external rotation ROM (p < 0.0001) was associated with poor hip function (HHS: lowest scores in any one subscale or more), but hip adduction ROM (p= 0.11) and hip flexion contracture (p= 0.12) was not associated with poor hip function (HHS: lowest scores in any one subscale or more) (NR: β and CI for all associations).  Cross-sectional analyses postoperatively (Linear regression):  Hip motion was correlated with function for high hip function group (HHS: highest scores for all of the subscales, R= 0.46, p < 0.0001), and for poor hip function group (HHS: lowest scores in any one subscale or more, R= 0.28, p < 0.0001) (NR: CI for all associations). |
|  | Yes | SV and LF  February/March 2024 | Dayton et al., (2016) [13] USA (English) Study design: Prospective cohort longitudinal Funding: NIH K23AG029978 and UL1TR000154; American College of Rheumatology REF/Abbot Health Professionals Graduate Student Research Preceptorship Award | To evaluate the correlations between changes in HOOS self-report function (ADL and pain subscales) and changes in functional performance (TUG, SCT, 6MW test) from before surgery to 1 month after THA and from 1 month to 6 months after THA | Age: 61.4 ± 8.3 N= 23 F (16), M (7)  THA approach: Posterior  Type: Unilateral LBP: NR Health conditions: NR | Performance-based: 6MWT TUG SCT | Function: HOOS ADL HOOS Pain | Preop: 2 weeks Postop: 1 and 6 months | Longitudinal analyses from preoperatively to 1 month postoperatively (Pearson product-moment correlations): Greater change in 6MWT was associated with greater change in function (HOOS ADL (r= 0.46, p= 0.03) and HOOS pain (r= 0.49, p= 0.02)) (NR: CI for all associations).  Change in TUG was not associated with change in function (HOOS ADL (r= 0.08, p= 0.68) and HOOS pain (r= -0.04, p= 0.87)) (NR: CI for all associations).  Change in SCT was not associated with change in function (HOOS ADL (r= 0.08, p= 0.72) and HOOS pain (r= 0.04, p= 0.85)) (NR: CI for all associations).  Longitudinal analyses from 1 month to 6 months postoperatively (Pearson product-moment correlations): Change in 6MWT was not associated with change in function (HOOS ADL (r= 0.34, p= 0.13) and HOOS pain (r= 0.07, p= 0.77)) (NR: CI for all associations). Change in TUG was not associated with change in function (HOOS ADL (r= 0.32, p= 0.16) and HOOS pain (r= 0.11, p= 0.64)) (NR: CI for all associations). Change in SCT was not associated with change in function (HOOS ADL (r= 0.27, p= 0.24) and HOOS pain (r= 0.01, p= 0.96)) (NR: CI for all associations). |
|  | Yes | SV and LF  February/March 2024 | Eyvazov et al., (2016) [14] Turkey (English) Study design: Cohort longitudinal Funding: None | To verify whether any significant correlation exists between improvement in LBP symptoms and changes in sagittal balance and trunk postural balance after surgery | Age (Male): 61.7 ± 6.4  Age (Female): 60.3 ± 7.9 N= 28 F (17), M (11)  THA approach: Anterolateral  Type: Unilateral LBP: Yes Health conditions: NR | Spinopelvic alignment: Sagittal vertical axis (X-ray)  Impairment-based: Postural stability test (PST) Limits of stability test (LOS) Clinical test of sensory integration of balance (CTSIB) (Biodex) | LBP:  VAS ODI | Preop Postop: 6 months | longitudinal analyses postoperatively (SCC):  Change in sagittal vertical axis (rₛ= 0.37, CIs= 0.01 to 0.74, p= 0.05), PST (rₛ= 0.15, CIs= -0.23 to 0.53, p= 0.44), LOS (rₛ= 0.18, CIs= -0.20 to 0.56, p= 0.36), and CTSIB (rₛ= -0.11, CIs= -0.50 to 0.27, p= 0.56) were not associated with change in LBP (VAS). Change in sagittal vertical axis (rₛ= 0.09, CIs= -0.28 to 0.44, p= 0.64), PST (rₛ= 0.32, CIs= -0.04 to 0.62, p= 0.05), LOS (rₛ= -0.26, CIs= -0.57 to 0.11, p= 0.18), and CTSIB (rₛ= 0.08, CIs= -0.29 to 0.43, p= 0.67) were not associated with change in LBP (ODI). |
|  | Yes | SV and LF  February/March 2024 | Fallahzadeh et al., (2022) [15] USA (English) Study design: Cohort longitudinal Funding: NIH grants R35GM138353, R35GM137936, AG058417, HL13984401, NS114926, DA050960, AG065744, Fondation des Gueules Cassées, Philippe Foundation | Objectively quantify the temporal pattern of physical recovery in individual patients | Age: 63 (NR: SD) N= 49 F (26), M (23)  THA approach: NR  Type: NR LBP: NR Health conditions: NR | Activity level in natural environment: Physical activity (ActiGraph smartwatch) | Fatigue: Surgical Recovery Scale (SRS)  Pain and function: WOMAC pain WOMAC function | PROMS: Preop Postop: 1 to 3 days Bi-weekly after discharge weeks 1 to 4 Weekly for postoperative weeks 5 to 6  Physical activity: Preop: 5 days Postop: 40 days | Longitudinal analyses from preoperative to postoperative (SCC): Physical recovery trajectory was associated with fatigue trajectory (SRS, p < 0.003, NR: rₛ and CI).  Physical recovery trajectory was not associated with pain and function trajectories (WOMAC pain and WOMAC function, NR: rₛ, p value, and CI). |
|  | Yes | SV and RR  February/March 2024 | Foucher et al., (2010) [16] USA (English) Study design: Cross-sectional Funding: Promotion of Doctoral Studies Award from the Foundation for Physical Therapy; Zimmer Germany and Martin−Luther−Universität Halle Wittenberg | To determine whether the relationship between gait laboratory and habitual speeds was related to clinical indices | Age: 60 ± 9 N= 26 F (11), M (15)  THA approach: NR Type: Unilateral LBP: NR Health conditions: NR Diagnosis was reported as hip OA by the study author (February/March 2024). | Impairment-based: Gait laboratory speed (Optoelectronic cameras and force plate)  Activity level in natural environment: Habitual speed (Activity monitor) | Clinical indices: WOMAC pain WOMAC stiffness WOMAC function WOMAC total | Postop: 3 weeks 12 months | Cross−sectional analyses 3 weeks postoperatively (SCC):  Gait laboratory speed was not associated with pain (WOMAC pain, rₛ= -0.25, p= 0.21), stiffness (WOMAC stiffness, rₛ= -0.28, p= 0.16), function (WOMAC function, rₛ= -0.38, p= 0.05), and clinical indices (WOMAC total, rₛ= -0.34, p= 0.09) (NR: CI for all associations). Habitual speed was not associated with pain (WOMAC pain, rₛ= -0.32, p= 0.11), but greater habitual speed was associated with lower stiffness (WOMAC stiffness, rₛ= -0.41, p= 0.03), better function (WOMAC function, rₛ= -0.43, p= 0.03), and better clinical indices (WOMAC total, rₛ= -0.43, p= 0.03) (NR: CI for all associations).  Cross−sectional analyses 12 months postoperatively (SCC):  Gait laboratory speed was not associated with pain (WOMAC pain, rₛ= -0.19, p= 0.39), stiffness (WOMAC stiffness, rₛ= -0.15, p= 0.50), function (WOMAC function, rₛ= -0.21, p= 0.35), and clinical indices (WOMAC total, rₛ= -0.21, p= 0.34) (NR: CI for all associations).  Habitual speed was not associated with pain (WOMAC pain, rₛ= 0.04, p= 0.86), stiffness (WOMAC stiffness, rₛ= -0.13, p= 0.57), function (WOMAC function, rₛ= -0.06, p= 0.77), and clinical indices (WOMAC total, rₛ= 0.22, p= 0.34) (NR: CI for all associations). |
|  | Yes | SV and RR  February/March 2024 | Foucher et al., (2018) [17] USA (English) Study design: Cross-sectional  Funding: National Institute on Aging; Grant number: R21AG052111, University of Illinois at Chicago, and campus Research Board Pilot Research Program | To explore the association of fatigue and abductor strength on self-reported physical activity in people with THR | Age: 56.8 ± 8.3 N= 16 F (11), M (5)  THA approach: No restriction Type: Cannot determined LBP: NR Health conditions: NR Diagnosis was reported as hip OA by the study author (February/March 2024). | Impairment-based: Hip abductor strength (Dynamometer) | Fatigue: PROMIS Fatigue Short Form  Activity Level UCLA activity | Postop: 23.8 months | Cross-sectional analyses postoperatively (Linear regression):  Greater hip abductor strength was associated with lower fatigue (PROMIS Fatigue Short Form, r²= 0.27) and better activity level (UCLA activity, r²= 0.48) (NR: p value and CI for all associations).  Cross-sectional analyses postoperatively (Linear regression):  Greater hip abductor strength was associated with lower fatigue (PROMIS Fatigue Short Form, Regression Coefficient= -10.5, CI= -20.2 to -0.7, p= 0.03) and better activity level (UCLA activity, Regression Coefficient= 4.7, CI= 2 to 7.5, p= 0.003).  Having hip abductor strength as dependent variable, greater hip abductor strength was associated with lower fatigue (PROMIS Fatigue Short Form, Regression Coefficient= -0.03, CI= -0.05 to -0.002, p= 0.03) and lower activity level (UCLA activity, Regression Coefficient= -5.8, CI= -11.7 to -1.3, p= 0.003). |
|  | Yes | SV and RR  February/March 2024 | Fujita et al., (2013) [18]  Japan (English) Study design: Cross-sectional Funding: NR | To assess  the association  between physical activity and QoL after THA | Age: 60.9 ± 9.1 N= 38 F (38), M (0)  THA approach: NR Type: Unilateral (n=33) or bilateral (n=5) LBP: NR  Health conditions: Hypertension Dyslipidemia Diabetes mellitus | Activity level in natural  environment: Daily steps Light intensity activity Moderate intensity activity Vigorous intensity activity  (Pedometers) | QoL: SF-8 Mental component  SF-8 Physical component  OHS | Postop: 6 and 12 months | Cross-sectional analysis 6 months postoperatively (SCC):  Higher daily steps were not associated with better quality of life (SF-8 mental component, rₛ= 0.09, p > 0.05, NR: CI), but it was associated with better quality of life (SF-8 physical component (rₛ= 0.34, p < 0.05) and OHS (rₛ= -0.32, p < 0.05)) (NR: CI for all associations). Light intensity activity was not associated with quality of life (SF-8 mental component (rₛ= 0.009, p > 0.05), SF-8 physical component (rₛ= 0.04, p > 0.05), and OHS (rₛ= -0.02, p > 0.05)) (NR: CI for all associations). Longer duration of moderate intensity activity was associated with better quality of life (SF-8 mental component (rₛ= 0.34, p < 0.01), SF-8 physical component (rₛ= 0.52, p < 0.01), and OHS (rₛ= -0.64, p < 0.01)) (NR: CI for all associations). Longer duration of vigorous intensity activity was not associated with better quality of life (SF-8 mental component score, rₛ= 0.17, p > 0.05, NR: CI), but it was associated with better quality of life (SF-8 physical component (rₛ= 0.43, p < 0.05) and OHS (rₛ= -0.47, p < 0.05)) (NR: CI for all associations).  Cross-sectional analyses 12 months postoperatively (SCC): Daily steps were not associated with quality of life (SF-8 mental component (rₛ= 0.10, p > 0.05), SF-8 physical component (rₛ= 0.18, p > 0.05), and OHS (rₛ= -0.21, p > 0.05)) (NR: CI for all associations). Light intensity activity was not associated with quality of life (SF-8 mental component (rₛ= 0.04, p > 0.05), SF-8 physical component (rₛ= 0.03, p > 0.05), and OHS (rₛ= 0.007, p > 0.05)) (NR: CI for all associations).  Longer duration of moderate intensity activity was not associated with better quality of life (SF-8 mental component score, rₛ= 0.21, p > 0.05), but it was associated with better quality of life (SF-8 physical component (rₛ= 0.32, p < 0.05) and OHS (rₛ= -0.48, p < 0.01)) (NR: CI for all associations). Longer duration of vigorous intensity activity was not associated with better quality of life (SF-8 mental component (rₛ= 0.13, p > 0.05) and SF-8 physical component (rₛ= 0.26, p > 0.05)), but it was associated with better quality of life (OHS score, rₛ= -0.38, p < 0.05) (NR: CI for all associations). |
|  | Yes | SV and LF  July 2024 | Fujita et al., (2022) [19] Japan (English) Study design: Cross-sectional Funding: None | To investigate the level of patient satisfaction after THA and to identify significant factors influencing satisfaction in a Japanese cohort | Age (Whole population): 69.1 ± 9.9 Age (Participants selected for muscle strength measurement): 68.3 ± 9.7 Age (Participants selected for activity monitoring measurement): 64.3 ± 6.6 N (Whole population)= 285 F (252), M (33)  N (Participants selected for muscle strength measurement)= 89 F (69), M (20)  N (Participants selected for activity monitoring measurement)= 26 F (23), M (3)  THA approach: NR Type: NR LBP: NR Health conditions: NR | Impairment-based: Hip flexion ROM Hip abduction ROM Hip external rotation ROM (NR: device and the measurement method) Maximal isometric hip flexor strength Maximal isometric hip abductor strength Maximal isometric knee extensor strength (Hand-held dynamometer)  Activity level in natural environment: Daily steps Number of standings per day (Tri-axial accelerometer monitor) | Physical activity: OHS activities of daily living (OHS ADL) UCLA activity score | Postop in whole population: 43 months Postop in muscle strength measurement group:  45 months Postop in activity monitoring measurement group: 11 months Postop in ROM group: NR | Cross-sectional analysis postoperatively (Stepwise multiple regression): Hip flexion ROM (β= 0.09, CIs= -0.04 to 0.09, p= 0.56), hip abduction ROM (β= -0.11, CIs= -0.15 to 0.05, p= 0.71), hip external rotation ROM (β= 0.09, CIs= -0.04 to 0.10, p= 0.34), maximal isometric hip flexor strength (β= 0.02, CIs= -0.17 to 0.22, p= 0.59), and maximal isometric knee extensor strength (β= -0.22, CIs= -0.27 to 0.04, p= 0.23) were not associated with physical activity (OHS ADL). However, greater maximal isometric hip abductor strength was associated with higher physical activity (OHS ADL, β= 0.32, CIs= 0.02 to 0.49, p= 0.04). Hip flexion ROM (β= 0.19, CIs= -0.01 to 0.08, p= 0.71), hip abduction ROM (β= -0.05, CIs= -0.08 to 0.05, p= 0.39), hip external rotation ROM (β= -0.08, CIs= -0.06 to 0.02, p= 0.89), maximal isometric hip flexor strength (β= -0.01, CIs= -0.13 to 0.12, p= 0.83), and maximal isometric knee extensor strength (β= -0.01, CIs= -0.10 to 0.09, p= 0.37) were not associated with physical activity (UCLA activity). However, greater maximal isometric hip abductor strength was associated with higher physical activity (UCLA activity score, β= 0.10, CIs= -0.09 to 0.21, p= 0.01).  Cross-sectional analysis postoperatively (SCC): Greater daily steps were associated with better physical activity (OHS ADL, rₛ= 0.48, p= 0.02), but number of standings per day was not associated with physical activity (OHS ADL, rₛ= -0.11, p= 0.62) (NR: CI for all associations). |
|  | Yes | SV and LF  February/March 2024 | Goeb et al., (2021) [20] USA (English) Study design: Cohort longitudinal Funding: None | To investigate whether wrist-based activity trackers will accurately provide valuable data to assess the postoperative functional activity | Age: 60.4 ± 5.7 N= 72 F (30), M (42)  THA approach: Posterolateral  Type: Unilateral/ Dual mobility in 6 patients LBP: NR Health conditions: NR  Diagnosis was reported as hip OA by the study author (February/March 2024). | Activity level in natural environment: The number of steps (Wrist-based activity tracker device) | Clinical outcome: HOOS JR  Function: LEFS  Pain: The questionnaire was not reported | Preop: 1week Postop: 6 weeks | Longitudinal analyses from preoperatively to postoperatively (Repeated measures linear mixed modeling): The average number of steps was associated with improvement in clinical outcome (HOOS JR, ß= 0.73, CIs= 0.16 to 1.31, p= 0.01), improvement in function (LEFS, ß= 1.39, CIs= 0.49 to 2.30, p= 0.002), improvement in pain at worse (NR: the questionnaire, ß= -1.20, CIs= -1.98 to -0.43, p= 0.002), and improvement in pain at best over time (NR: the questionnaire, ß= -0.45, CIs= -0.89 to -0.02, p= 0.04). |
|  | Yes | SV and LF  July 2024 | Harada et al., (2024) [21] Japan (English) Study design: Prospective cohort longitudinal Funding: Grant from the Ogata Memorial Foundation, Inc. No. 135) and Medical Care Education Research Foundation | To compare objective and subjective measurements of activity levels in patients undergoing THA preoperatively, three months and one year following surgery, and investigated the factors that predicts the objective activity level after THA | Age: 68.6 ± 8.6 N= 42 F (33), M (9)  THA approach: Posterolateral  Type: Unilateral LBP: NR  Health conditions: NR | Activity level in natural environment: Daily steps Number of sit-to-stand transitions Upright time (Tri-axial accelerometer monitor) | Physical activity: UCLA activity | Preop: Within 2 weeks Postop: 3 months and 1 year | Longitudinal analysis from preoperatively to postoperatively (Multivariate linear regression): Daily steps at 3 month postoperatively (p= 0.46) and at 1 year postoperatively (p= 0.99) were not associated with preoperative physical activity (UCLA activity, NR: β and CI for all associations). Number of sit-to-stand transitions at 3 month postoperatively (p= 0.95) and at 1 year postoperatively (p= 0.29) were not associated with preoperative physical activity (UCLA activity, NR: β and CI for all associations). Upright time at 3 month postoperatively (p= 0.31) and at 1 year postoperatively (p= 0.26) were not associated with preoperative physical activity (UCLA activity, NR: β and CI for all associations). |
|  | Yes | SV and RR  February/March 2024 | Holm et al., (2013) [22] Denmark (English) Study design: Prospective cohort longitudinal  Funding: A grant from The Lundbeck Foundation, Hellerup, Denmark | To explore relationships between changes in hip muscle strength and changes in hip pain | Age: 65.9 ± 7.2 N= 30 F (21), M (9)  THA approach: Posterior  Type: Unilateral LBP: NR  Health conditions: NR | Impairment-based: Hip adduction strength Hip abduction strength Hip flexion strength (Dynamometer) Leg-press power (Nottingham Power Rig) | Hip pain: VAS | Preop: 1 week Postop: 1 and 7 days | Longitudinal analyses from preoperative to postoperative (Pearson's or Spearman's correlation coefficient): Change in hip adduction strength, hip abduction strength, hip flexion strength, and leg press power were not associated with change in hip pain (VAS, NR: r, p value, and CI for all associations). |
|  | Yes | SV and RR  February/March 2024 | Holstege et al., (2011) [23]  Netherlands (English) Study design: Prospective cohort longitudinal Funding: NR | To determine the preoperative strength of the muscle group of the lower extremity that is most important in predicting functional recovery after primary unilateral THR | Age: 72.1 ± 6.4 N= 37 F (28), M (9)  THA approach: Lateral (n=10) and posterior (n=27) Type: Unilateral LBP: NR Health conditions: 22 participants had comorbid conditions. | Impairment-based: Hip flexors strength of the operated side Hip extensors strength of the operated side Hip adductors strength of the operated side Hip abductors strength of the operated side Knee flexors strength of the operated side Knee extensors strength of the operated side (Dynamometer) | Functional recovery: WOMAC PF  Mental health: SF−36 mental health   Hip pain: VAS | Preop: 2 weeks Postop: 12 weeks | Longitudinal analyses from preoperative to postoperative (Multivariable linear regression): Preoperative hip flexors strength (ß= -0.08, p= 0.11), preoperative hip extensors strength (ß= -0.02, p= 0.49), preoperative hip adductors strength (ß= -0.05, p= 0.38), preoperative hip abductors strength (ß= -0.07, p= 0.06), and preoperative knee flexors strength (ß= -0.09, p= 0.07) could not predict postoperative functional recovery (WOMAC PF). However, preoperative greater knee extensor strength could predict better postoperative functional recovery (WOMAC PF, ß= -0.10, p= 0.004) (NR: CI for all associations). Preoperative hip flexors strength, preoperative hip extensors strength, preoperative hip adductors strength, preoperative hip abductors strength, preoperative knee flexors strength, and preoperative knee extensors strength could not predict postoperative mental health and hip pain (SF−36 mental health and VAS, NR: ß, p value, and CI for all associations) |
|  | Yes | SV and RR  February/March 2024 | Huang et al., (2019) [24] USA (English) Study design: Cross-sectional Funding: University of Illinois at the Chicago Campus Research Board Pilot Research program; Grant number: NIH R21AG052111 | To evaluate step length asymmetry and its links to physical function after THR | Age: 57.0 ± 8.0 N= 18 F (12), M (6)  THA approach: No restriction Type: No restriction LBP: NR  Health conditions: NR Diagnosis was reported as hip OA by the study author (February/March 2024). | Performance-based: 6MWT  Impairment-based: Gait parameters:  Mechanical energy exchange Step length asymmetry (Optoelectronic cameras motion analysis system) | Fatigue: PROMIS Fatigue | Postop: 25 months | Cross-sectional analyses postoperatively (PCC):  Better performance in 6MWT was associated with lower fatigue (PROMIS Fatigue, r= -0.61, p= 0.01, NR: CI). Higher mechanical energy exchange was associated with higher fatigue (PROMIS Fatigue, r= 0.50, p= 0.04, NR: CI).  Step length asymmetry was not associated with fatigue (PROMIS Fatigue, r= 0.12, p= 0.66, NR: CI). |
|  | Yes | SV and RR  February 2025 | Jelsma et al., (2021) [25]  Netherlands (English)  Study design: Cross-sectional  Funding: None | To investigate how patient-reported outcome measures of various dimensions, general health, disease specific outcome and in particular physical function, joint awareness and  self-perceived activity levels are correlated with objectively measured physical activity parameters derived from  wearable activity-monitors in subjects with a hip arthroplasty | Age: 60 (NR: SD)  N= 16  F (4), M (12)  THA approach: Posterolateral (7) and Straight lateral (9)  Type: Unilateral  LBP: NR  Health conditions:  NR | Activity level in natural environment:  Time sitting  Time standing  Time walking  Time cycling  Total time active  Sit-Stand Transfers  Steps  Cadence  Intensity Peaks <2.0g  Intensity Peaks >2.0g  (3-axis accelerometer, gyroscope,  and magnetometer) | Joint awareness:  FJS-12  Pain and function:  HOOS-PS  Physical activity:  SQUASH  General health: physical functioning subscale:  SF36- PF | Postop:  10 years | Cross-sectional analyses postoperative (PCC) (Data for THA population provided by the study author (February 2025)):  Time sitting (r= -0.05, p= 0.82), time standing (r= 0.34, p= 0.19), time cycling (r= 0.5, p= 0.82), number of sit-stand transfers (r= 0.15, p= 0.57), and cadence (r= 0.46, p= 0.07) were not associated with joint awareness (FJS-12, NR: CI for all associations). Higher time walking (r= 0.66, p= 0.005), higher total time active (r= 0.51, p= 0.04), higher numbers of steps (r= 0.57, p= 0.02), higher number of intensity peaks <2.0g (r= 0.54, p= 0.02), and higher number of intensity peaks >2.0g (r= 0.52, p= 0.03) were associated with better joint awareness (FJS-12, NR: CI for all associations).  Time sitting (r= -0.39, p= 0.19), time standing (r= 0.55, p= 0.06), time cycling (r= 0.01, p= 0.95), number of sit-stand transfers (r= 0.06, p= 0.84), cadence (r= 0.49, p= 0.10), number of intensity peaks <2.0g (r= 0.53, p= 0.07), and number of intensity peaks >2.0g (r= 0.45, p= 0.13) were not associated with physical function (HOOS-PS, NR: CI for all associations). Higher time walking (r= 0.73, p= 0.006), higher total time active (r= 0.61, p= 0.03), and higher numbers of steps (r= 0.67, p= 0.01) were associated with better physical function (HOOS-PS, NR: CI for all associations).  Time sitting (r= -0.31, p= 0.30), time standing (r= 0.07, p= 0.80), time walking (r= 0.26, p= 0.37), time cycling (r= -0.11, p= 0.71), total time active (r= 0.22, p= 0.47), number of sit-stand transfers (r= 0.11, p= 0.70), numbers of steps (r= 0.17, p= 0.56), cadence (r= 0.09, p= 0.76), number of intensity peaks <2.0g (r= 0.20, p= 0.50), and number of intensity peaks >2.0g (r= 0.30, p= 0.31) were not associated with physical activity (SQUASH, NR: CI for all associations).  Time cycling (r= 0.12, p= 0.65), number of sit-stand transfers (r= 0.08, p= 0.76), cadence (r= 0.41, p= 0.11), and number of intensity peaks >2.0g (r= 0.46, p= 0.06) were not associated with general health: physical functioning subscale (SF36- PF, NR: CI for all associations). Higher time sitting (r= -0.59, p= 0.01) was associated with worse general health: physical functioning subscale (SF36- PF, NR: CI), but higher time standing (r= 0.53, p= 0.03), higher time walking (r= 0.76, p= 0.00), higher total time active (r= 0.72, p= 0.002), higher numbers of steps (r= 0.71, p= 0.002), and higher number of intensity peaks <2.0g (r= 0.54, p= 0.02) were associated with better general health: physical functioning subscale (SF36- PF, NR: CI for all associations). |
|  | Yes | SV and RR  February/March 2024 | Kamimura et al., (2014) [26] Japan (English) Study design: Prospective cohort longitudinal Funding: NR | To identify whether preoperative pain can predict improvement in ambulation ability at each postoperative time point | Age: 67.6 ± 10.2 N= 48 F (48), M (0)  THA approach: Posterolateral  Type: Unilateral LBP: NR  Health conditions: NR | Performance-based: TUG | Hip pain: VAS | Preop Postop: 3 weeks 4 and 7 months | Longitudinal analyses from preoperative to postoperative (Pearson’s rank correlation coefficients):  Postoperative TUG test at 3 weeks postoperatively (r= -0.002), at 4 months postoperatively (r= 0.006), and at 7 months postoperatively (r= 0.28) could not be predicted by preoperative hip pain (VAS, NR: p value and CI for all associations). |
|  | Yes | SV and RR  February/March 2024 | Kaufmann et al., (2022) [27]  Switzerland (English) Study design: Cohort longitudinal Funding: Deutsche Arthrose‐Hilfe e.V.; Schweizerische Gesellschaft für Orthopädie und Traumotologie; Merian Iselin Foundation, Basel, Switzerland; Department of Orthopaedics and Traumatology and the Department of Surgery of the University of Basel, Grant/Awar Number: Research Award | To assess the association between PROMs and hip kinematics during walking | Age: 62.7 ± 10.7 N= 24 F (10), M (14)  THA approach: Anterior (n= 19), anterolateral (n=4), and lateral (n=1) Type: Unilateral LBP: NR  Health conditions: NR | Impairment-based: Dynamic hip ROM (Accelerometers, gyroscopes, and magnetometer) | Symptoms and function: HOOS pain  HOOS symptoms  HOOS ADL  HOOS sport/recreation  HOOS quality of life | Preop Postop: 1 year | Longitudinal analyses from preoperative to postoperative (SCC): Greater dynamic hip ROM during the stance phase of walking was associated with lower symptoms (HOOS pain (rₛ= 0.56, p <0.001) and HOOS symptoms (rₛ= 0.58, p <0.001)) and better function (HOOS ADL (rₛ= 0.55, p <0.001), HOOS sport/recreation (rₛ= 0.49, p <0.001), and HOOS quality of life (rₛ= 0.54, p <0.001)) (NR: CI for all associations).   Longitudinal analyses from preoperative to postoperative (Stepwise linear regression): Greater change in dynamic hip ROM during the stance phase of walking was a predictor of greater change in symptoms (HOOS symptoms, r²= 0.74, p < 0.001, NR: CI). |
|  | Yes | SV and RR  July 2024 | Kirschner et al., (2023) [28] Germany (English) Study design: Cross-sectional and prospective cohort longitudinal Funding: None | To investigate the relationships between symmetry-based, performance-based, and functional outcome measures in THA patients | Age: 63.5 ± 8.3 N= 24 F (6), M (18)  THA approach: Anterolateral  Type: Cannot determined LBP: NR  Health conditions: NR | Performance-Based: SCT 10 MWT TUG  Impairment-based: Maximal isometric torque of quadriceps muscle strength (Hand-held dynamometer)  Weight-Bearing Chair-Rising Test (CRT)  (Force plates) | Function: HHS Activities of daily living (HHS ADL) HHS Walk  Hip pain:  HHS pain | Preop: T0: 1 day Postop: T1: 3.96 days T2: 8.62 days T3: 9.29 weeks | Cross-sectional analysis postoperatively (Pearson's or Spearman's correlation coefficient): Better performance in SCT was associated with better function at T3 (HHS Walk, r= -0.60, NR: p value and CI). There were no high or very high correlations (r < 0.70) between SCT with function and hip pain at T3 (HHS ADL and HHS pain, NR: r, p value and CI for all associations)  There were no high or very high correlations (r < 0.70) between 10 MWT, TUG, maximal isometric torque of quadriceps muscle strength, and CRT with function and hip pain at T3 (HHS ADL, HHS Walk, and HHS pain, NR: r, p value, and CI for all associations).  Cross-sectional analysis postoperatively (Coefficient of determination): Better performance in SCT at T3 was associated with better function at T3 (HHS Walk, r² = 0.36).   Longitudinal analysis from preoperatively to postoperatively (Pearson's or Spearman's correlation coefficient): Better performance in 10 MWT at T3 was associated with better function at T0 (HHS Walk, r= -0.60, NR: p value and CI).  Better performance in SCT at T0 was associated with better function at T3 (HHS ADL (r= -0.65, NR: p value and CI) and HHS Walk (r= -0.61, NR: p value and CI)). Lower difference in maximal isometric torque of quadriceps muscle strength in the involved and uninvolved limbs at T0 was associated with better function at T3 (HHS Walk, NR: r, p value, and CI).  The rest of the correlations were lower than moderate (NR: outcome measures, timepoints, r, p value, and CI)  Longitudinal analysis from preoperatively to postoperatively (Coefficient of determination): Better performance in 10 MWT at T3 could be predicted by better function at T0 (HHS Walk, r² = 0.36). Better performance in SCT at T0 could predict better function at T3 (HHS ADL, r² = 0.42). Better performance in SCT at T0 could predict better function at T3 (HHS Walk, r² = 0.37). Lower difference in maximal isometric torque of quadriceps muscle strength in the involved and uninvolved limbs at T0 could predict better function at T3 (HHS Walk, from r² =0.36 to 0.42) |
|  | Yes | SV and RR  February/March 2024 | Kobayashi et al., (2023) [29] Japan (English) Study design: Cohort longitudinal Funding: None | To investigate the effect of changes in whole-body alignment on ipsilateral knee pain in patients after THA | Age: 63 (NR: SD) N (Whole population)= 94 F (73), M (21)  N (Population with knee pain)= 61  F and M: NR THA approach: Lateral (68) and anterolateral (8)  Type: Unilateral LBP: NR Health conditions: NR | Spinopelvic alignment: Sagittal vertical axis  (X-Ray) | Knee pain: VAS | Preop Postop: 3 months | Longitudinal analyses from preoperative to postoperative (PCC): Greater preoperative sagittal vertical axis was associated with higher postoperative knee pain (VAS, r= 0.28, p= 0.01, NR: CI).  Longitudinal analyses from preoperative to postoperative (Multivariate regression analysis): Preoperative sagittal vertical axis can predict postoperative knee pain (VAS, B= 0.12, standardized ß= 0.27, p= 0.03, NR: CI). |
|  | Yes | SV and LF  February/March 2024 | Lin et al., (2022) China (English) [30] Study design: Cross-sectional Funding: LXB was supported by the Research and Development Fund of Quanzhou City (2017Z010), the Natural Science Foundation of Fujian Province (2017J01277), the Scientific Research Project for Young and Middle-aged Teachers of the Fujian Province Education Department (JAT160217), and the Training Project for Young and Middle-aged Talented Personnel of the Fujian Provincial Commission of Health and Family Planning (2016-ZQN-52). WWH was supported by the National Natural Science Foundation of China (81272161), the Programme for Medical Innovation of Fujian Province (2011-CXB-21, 2019-CX-30), the Research and Development Fund of Quanzhou City (2017Z007), the Fund of Professorship for Academic Development of Fujian Medical University (JS11001), the Scientific Research Project of Department of Education, Fujian Province (JB09117, JB11050), the Special Fund for Training Excellent Talents of Quanzhou City (08A17, 11A02), and the Fund for Leading Talents of Quanzhou City (QuanKe2014[No.61]). SMB was funded by a VIDI grant (016.Vidi.178.014) from the Dutch Organization for Scientific Research (NWO) | To identify factors that would predict falls in the year after THA | Age: 63.7 ± 4.6 N= 12 F (4), M (8)  THA approach: Lateral  Type: NR LBP: NR Health conditions: NR | Impairment-based: Affected side maximum hip abduction strength Unaffected side maximum hip abduction strength Averaged (over the sides) maximum hip abduction strength (Dynamometer) Maximum walking speed (Treadmill) Stride time Step width Affected stance time Unaffected stance time Range of frontal plane Centre of Mass (CoM) movements Peak speed of frontal plane CoM movements towards the affected side Peak speed of frontal plane CoM movements towards the unaffected side Affected side frontal plane margin of stability Unaffected side frontal plane margin of stability Affected side extrapolated frontal plane margin of stability Unaffected side extrapolated frontal plane margin of stability Lyapunov exponent (Motion analysis: OptoTrak) | Pain: VAS  Fear of falling: Falls Efficacy Scale International (‘FES-I’) | Postop: 12.4 months | Cross-sectional analysis postoperatively (Pearson correlation matrix): Greater affected side maximum hip abduction strength was associated with higher pain before measurement (VAS, r= 0.80, p ≤0.01) and higher pain after measurement (VAS, r= 0.81, p ≤0.01) (NR: CI for all associations). Greater unaffected side maximum hip abduction strength was associated with higher pain before measurement (VAS, r= 0.73, p ≤0.01) and higher pain after measurement (VAS, r= 0.75, p ≤0.01) (NR: CI for all associations). Greater averaged maximum hip abduction strength was associated with higher pain before measurement (VAS, r= 0.78, p ≤0.01) and higher pain after measurement (VAS, r= 0.79, p ≤0.01) (NR: CI for all associations). Maximum walking speed, stride time, step width, affected stance time, unaffected stance time, range of frontal plane CoM movements, peak speed of frontal plane CoM movements towards the affected side, peak speed of frontal plane CoM movements towards the unaffected side, affected side frontal plane margin of stability, unaffected side frontal plane margin of stability, affected side extrapolated frontal plane margin of stability, unaffected side extrapolated frontal plane margin of stability, lyapunov exponent were not associated with pain before and after measurement (VAS, p < 0.05, NR: r and CI for all associations). Affected side maximum hip abduction strength, unaffected side maximum hip abduction strength, averaged (over the sides) maximum hip abduction strength, maximum walking speed, stride time, step width, affected stance time, unaffected stance time, range of frontal plane CoM movements, peak speed of frontal plane CoM movements towards the affected side, peak speed of frontal plane CoM movements towards the unaffected side, affected side frontal plane margin of stability, unaffected side frontal plane margin of stability, affected side extrapolated frontal plane margin of stability, unaffected side extrapolated frontal plane margin of stability, lyapunov exponent were not associated with fear of falling (FES-I, p < 0.05, NR: r and CI for all associations). |
|  | Yes | SV and RR  February/March 2024 | Lindemann et al., (2006) [31] Germany (English) Study design: Cross-sectional and prospective cohort longitudinal Funding: The Bethesda Geriatrische Klinik Ulm, and the Rehabilitationskrankenhaus Ulm | To investigate the correlation between objective and subjective measurements in the evaluation of patients with THR | Age: 67.2 ± 5.25 N= 17 F (8), M (9)  THA approach: Transgluteal  Type: Unilateral LBP: NR Health conditions: NR | Impairment-based: Gait parameters: Gait speed Stride length Thigh flexion-extension Stance phase ratio (Gyroscopes) | Health status: WOMAC total WOMAC stiffness WOMAC function WOMAC pain | Preop: 1 day Postop: 3 months | Cross-sectional analysis postoperatively (SCC):  Higher gait speed was weakly associated with both better health status (WOMAC (rₛ= -0.16), WOMAC stiffness (rₛ= -0.06), WOMAC function (rₛ= -0.12)) and worse health status (WOMAC pain (rₛ= 0.05)) (NR: p value and CI for all associations). Higher gait speed coefficient of variation was weakly associated with better health status (WOMAC (rₛ= -0.10), WOMAC pain (rₛ= -0.15), WOMAC function (rₛ= -0.06), WOMAC stiffness (rₛ= -0.23)) (NR: p value and CI for all associations). Greater stride length was weakly associated with worse health status (WOMAC (rₛ= 0.14), WOMAC pain (rₛ= 0.04), WOMAC stiffness, (rₛ= 0.12), and WOMAC function (rₛ= 0.20)) (NR: p value and CI for all associations). Greater stride length coefficient of variation was weakly associated with both worse health status (WOMAC, rₛ= 0.02) and better health status (WOMAC pain (rₛ= -0.06), WOMAC function (rₛ= -0.05), and WOMAC stiffness (rₛ= -0.21)) (NR: p value and CI for all associations). Greater thigh flexion-extension was weakly associated with both worse health status (WOMAC, rₛ= 0.05) and better health status (WOMAC pain (rₛ= -0.07), WOMAC stiffness (rₛ= -0.10), and WOMAC function (rₛ= -0.03)) (NR: p value and CI for all associations). Greater stance phase ratio was weakly associated with both better health status (WOMAC (rₛ= -0.17), WOMAC stiffness (rₛ= -0.27), and WOMAC function (rₛ= -0.21)) and worse health status (WOMAC pain, rₛ= 0.03) (NR: p value and CI for all associations).  Longitudinal analyses from preoperative to postoperative (SCC): Greater change in gait speed was associated with lower change in health status (WOMAC (rₛ= -0.6), WOMAC stiffness (rₛ= -0.59), WOMAC function (rₛ= -0.62)), and it was weakly associated with pain (WOMAC pain (rₛ= -0.39)) (NR: p value and CI for all associations). Greater change in gait speed coefficient of variation was weakly associated with greater change in health status (WOMAC (rₛ= 0.18), WOMAC stiffness (rₛ= 0.28), WOMAC function (rₛ= 0.13), and WOMAC pain (rₛ= 0.36)) (NR: p value and CI for all associations). Greater change in stride length was associated with lower change in health status (WOMAC (rₛ= -0.6), WOMAC stiffness (rₛ= -0.5), WOMAC function (rₛ= -0.62), and WOMAC pain (rₛ= -0.42)) (NR: p value and CI for all associations). Greater change in stride length coefficient of variation was weakly associated with greater change in health status (WOMAC (rₛ= 0.32), WOMAC function (rₛ= 0.38), WOMAC stiffness (rₛ= 0.09), and WOMAC pain (rₛ= 0.01)) (NR: p value and CI for all associations). Greater change in thigh flexion-extension was associated with lower change in health status (WOMAC (rₛ= -0.7), WOMAC stiffness (rₛ= -0.64), WOMAC function (rₛ= -0.72), and WOMAC pain (rₛ= -0.55)) (NR: p value and CI for all associations). Greater change in stance phase ratio was associated with lower change in health status (WOMAC (rₛ= -0.66), WOMAC function (rₛ= -0.66), WOMAC pain (rₛ= -0.49), WOMAC stiffness (rₛ= -0.22)) (NR: p value and CI for all associations). |
|  | Yes | SV and LF  February/March 2024 | Lyman et al., (2020) [32]  USA (English) Study design: Cohort longitudinal Funding: Weill Cornell Medicine, Grant No. 5UL1TR000457 | To investigate how well step counts and PROMs scores were correlated | Age (Whole population): 59.0 ± 10.2 N (Whole population) = 130 F (66), M (64)  N (Population completed assessment at 6 months) = 83 F and M: NR  THA approach: NR Type: Unilateral LBP: NR  Health conditions: NR | Activity level in natural environment: Daily steps (A phone’s accelerometer: The Moves app) | Pain: NRS  QoL: HOOS quality of life  Hip health HOOS JR | Preop  Postop: 6 months | Longitudinal analyses postoperatively (Bivariate linear mixed modeling): Greater daily steps were associated with lower pain (NRS, correlation coefficient= -0.12, CIs= -0.22 to 0.01), better quality of life (HOOS quality of life, correlation coefficient= 0.10, CIs= -0.02 to 0.19), and better hip health (HOOS JR, correlation coefficient= 0.17, CIs= 0.07 to 0.26) (NR: p value for all associations). |
|  | Yes | SV and RR  February/March 2024 | Mahmood et al., (2016) [33] Sweden (English) Study design: Prospective cohort longitudinal Funding: NR | To investigate the association between femoral offset and patient reported hip function and abductor muscle strength | Age (whole population): NR N= 222 F (107), M (115) Age (Decreased femoral offset population): 71 (NR: SD) N (Decreased femoral offset population)= 71 F (33), M (38)  Age (Restored femoral offset population): 68 (NR: SD) N (Restored femoral offset population)= 73 F (35), M (38)  Age (Increased femoral offset population): 65 (NR: SD) N (Increased femoral offset population)= 78  F (39), M (39) THA approach: Posterolateral Type: Unilateral LBP: NR  Health conditions: NR | Impairment-based: Hip abductor strength (Dynamometer) | Function: WOMAC | Preop Postop: 12 to 15 months | Longitudinal analyses from preoperative to postoperative (Regression): Postoperative hip abductor strength was not associated with preoperative function (WOMAC, coefficient= -0.06, CIs= -0.33 to 0.20, p= 0.6). |
|  | Yes | SV and LF  February/March 2024 | Mark‐Christensen et al., (2019) [34] Denmark (English) Study design: Prospective cohort longitudinal Funding: A grant from the Lundbeck Foundation, Hellerup Denmark | To investigate prospectively the patient‐reported and objectively assessed functional recovery following THA | Age: 72 ± 8.5 N= 59  F (37), M (22)  THA approach: NR  Type: Unilateral LBP: NR Health conditions: NR Diagnosis was reported as hip OA by the study author (February/March 2024). | Performance-based: CST | Symptoms and function:  HOOS physical function HOOS sport and recreation HOOS Symptoms HOOS Pain HOOS Quality of life  Satisfaction: The Hospital for Special Surgery Total Hip Replacement Expectations Survey  Joint awareness: Forgotten Joint Scale  Expectations: Patient‐derived expectation questionnaire  Pain catastrophizing: Pain catastrophizing scale | Preop: 21 days Postop: 122 days | Longitudinal analyses from preoperative to postoperative (SCC): Change in CST was not associated with preoperative symptoms and function (HOOS physical function (rₛ= 0.21, p= 0.10, NR: CI), HOOS sport and recreation (NR: rₛ, p value, and CI), HOOS symptoms (rₛ= 0.02, p= 0.86, NR: CI), HOOS pain (NR: rₛ, p value, and CI), and HOOS quality of life (NR: rₛ, p value, and CI)). Change in CST was not associated with preoperative expectations (Patient‐derived expectation questionnaire (NR: rₛ, p value, and CI)) and preoperative pain catastrophizing (Pain catastrophizing scale (NR: rₛ, p value, and CI)). Greater change in CST was associated with higher change in symptoms and function (HOOS physical function, rₛ= 0.35, p= 0.007, NR: CI), but it was not associated with change in symptoms and function (HOOS sport and recreation (NR: rₛ, p value and CI), HOOS symptoms (NR: rₛ, p value and CI), HOOS pain (r²= 0.07, NR: rₛ, p value and CI), HOOS quality of life (r²= 0.05, NR: rₛ, p value and CI)) and change in pain catastrophizing (Pain catastrophizing scale (NR: rₛ, p value, and CI)). Change in CST was not associated with postoperative satisfaction (The Hospital for Special Surgery Total Hip Replacement Expectations Survey, r²= 0.09, NR: rₛ, p value, and CI). |
|  | Yes | SV and LF  February/March 2024 | McMeeken et al., (2007) [35] Australia (English) Study design: Cross-sectional Funding: The Centre for Molecular Biology and Medicine at the Epworth Medical Centre, Richmond, Australia | To investigate any potential relationship between muscle strength, function and pain | Age: 68.4 ± 2.1 N= 17 F (5), M (12)  THA approach: NR Type: NR  LBP: NR Health conditions: NR | Impairment-based: Quadriceps concentric peak performance Quadriceps eccentric peak performance  Performance-based: TUG | Function: Stair climbing scale | Postop: 8 to 12 weeks | Cross-sectional analyses postoperatively (PCC): Greater quadriceps concentric peak performance was associated with better function (Stair climbing scale, r= -0.46, p < 0.001, NR: CI). Greater quadriceps eccentric peak performance was associated with better function (Stair climbing scale, r= -0.46, p= 0.0003, NR: CI). Shorter time in TUG was associated with better function (Stair climbing scale, r= 0.68, NR: p value and CI). |
|  | Yes | SV and LF  February/March 2024 | Meessen et al., (2020) [36] Netherlands (English) Study design: Prospective cohort longitudinal Funding: None | To assess the association of pre-operative handgrip strength with the level of improvements of hip function and quality of life 1 year after THA | Age: 66.4 ± 9.5 N= 226 F (127), M (99)  THA approach: NR Type: Unilateral LBP: NR  Health conditions: NR | Impairment-based: Handgrip strength (Hydraulic hand dynamometer) | QoL: SF-36 physical health  SF-36 mental health   Function: HOOS pain HOOS quality of life  HOOS sport and recreation  HOOS symptoms  HOOS ADL | Preop Postop: 12 months | Longitudinal analysis from preoperatively to postoperatively (Multiple regression): Preoperative handgrip strength was not associated with change in quality of life (SF-36 physical health (ß= 0.13, CIs= -0.001 to 0.27, p= 0.05) and SF-36 mental health (ß= 0.07, CIs= -0.05 to 0.20, p= 0.25)). Greater preoperative handgrip strength was associated with change in function (HOOS pain (ß= 0.27, CIs= 0.01 to 0.52, p= 0.03), HOOS quality of life (ß= 0.31, CIs= 0.005 to 0.63, p= 0.04), HOOS sport and recreation (ß= 0.68, CIs= 0.20 to 1.15, p= 0.005), and HOOS symptoms (ß= 0.56, CIs= 0.22 to 0.90, p= 0.001)), but it was not associated with change in function (HOOS ADL, ß= 0.25, CIs= -0.03 to 0.54, p= 0.08). |
|  | Yes | SV and RR  February/March 2024 | Melchiorri et al., (2015) [37]  Italy (English) Study design: Cross-sectional Funding: NR | To investigate possible relationships between patient autonomy and abductor muscle strength | Age: 70.7 ± 6.3 N= 78 F (78), M (0)  THA approach: Lateral  Type: Unilateral LBP: NR  Health conditions: NR | Impairment-based: Hip abductor muscle strength: -5deg abduction Hip abductor muscle strength: 0deg abduction Hip abductor muscle strength: 10deg abduction Hip abductor muscle strength: 15deg abduction Hip abductor muscle strength: 20deg abduction (Dynamometer)  Performance-based: TUG | Function: Modified HHS WOMAC  QoL: SF-36: Total SF-36: Physical functioning SF-36: Role-physical SF-36: bodily pain SF-36: general health SF-36:Vitality SF-36: Social functioning  SF-36: Role, emotional SF-36: Mental health | Postop: 35 months | Cross-sectional analyses postoperatively (PCC):  Hip abductor muscle strength of the operated side at -5 degree (r= 0.09, p= 0.66), 0 degree (r= 0.22, p= 0.27), 10 degree (r= 0.18, p= 0.36), 15 degree (r= 0.22, p= 0.26), and 20 degree (r= 0.25, p= 0.22) were not associated with function (Modified HHS, NR: CI for all associations).  Hip abductor muscle strength of the contralateral side at -5 degree (r= -0.20, p= 0.31), 0 degree (r= -0.12, p= 0.53), 10 degree (r= -0.08, p= 0.67), 15 degree (r= -0.20, p= 0.32), and 20 degree (r= -0.27, p= 0.18) were not associated with function (Modified HHS, NR: CI for all associations).  Hip abductor muscle strength of the operated side at -5 degree (r= 0.10, p= 0.61), 0 degree (r= 0.19, p= 0.35), 10 degree (r= 0.14, p= 0.47), 15 degree (r= 0.20, p= 0.32), and 20 degree (r= 0.23, p= 0.25) were not associated with function (WOMAC, NR: CI for all associations). Hip abductor muscle strength of the contralateral side at -5 degree (r= -0.17, p= 0.40), 0 degree (r= -0.15, p= 0.46), 10 degree (r= -0.06, p= 0.76), 15 degree (r= -0.23, p= 0.24), and 20 degree (r= -0.22, p= 0.26) were not associated with function (WOMAC, NR: CI for all associations).  Hip abductor muscle strength of the operated side at -5 degree (r= 0.23, p= 0.29), 0 degree (r= 0.31, p= 0.15), 10 degree (r= 0.36, p= 0.09), 15 degree (r= 0.19, p= 0.38), and 20 degree (r= 0.31, p= 0.16) were not associated with quality of life (SF-36 score, NR: CI for all associations). Hip abductor muscle strength of the contralateral side at -5 degree (r= 0.06, p= 0.76), 0 degree (r= -0.02, p= 0.93), 10 degree (r= 0.18, p= 0.42), 15 degree (r= -0.04, p= 0.84), and 20 degree (r= -0.04, p= 0.84) were not associated with quality of life (SF-36, NR: CI for all associations).  Hip abductor muscle strength of the operated side at -5 degree (r= 0.15, p= 0.42), 0 degree (r= 0.26, p= 0.17), 10 degree (r= 0.34, p= 0.07), 15 degree (r= 0.22, p= 0.24), and 20 degree (r= 0.24, p= 0.22) were not associated with quality of life (SF-36 physical functioning, NR: CI for all associations). Hip abductor muscle strength of the contralateral side at -5 degree (r= 0.04, p= 0.81), 0 degree (r= -0.06, p= 0.76), 10 degree (r= 0.12, p= 0.54), 15 degree (r= -0.04, p= 0.80), and 20 degree (r= -0.02, p= 0.90) were not associated with quality of life (SF-36 physical functioning, NR: CI for all associations). Hip abductor muscle strength of the operated side at -5 degree (r= 0.27, p= 0.16), 0 degree (r= 0.34, p= 0.07), 10 degree (r= 0.35, p= 0.06), 15 degree (r= 0.30, p= 0.11), and 20 degree (r= 0.26, p= 0.16) were not associated with quality of life (SF-36 role physical, NR: CI for all associations).  Hip abductor muscle strength of the contralateral side at -5 degree (r= 0.02, p= 0.91), 0 degree (r= -0.04, p= 0.80), 10 degree (r= 0.06, p= 0.75), 15 degree (r= 0.09, p= 0.64), and 20 degree (r= 0.07, p= 0.71) were not associated with quality of life (SF-36 role physical, NR: CI for all associations). Hip abductor muscle strength of the operated side at -5 degree (r= 0.34, p= 0.07), 0 degree (r= 0.30, p= 0.05), 10 degree (r= 0.34, p= 0.07), 15 degree (r= 0.25, p= 0.19), and 20 degree (r= 0.32, p= 0.07) were not associated with quality of life (SF-36 bodily pain, NR: CI for all associations). Hip abductor muscle strength of the contralateral side at -5 degree (r= 0.14, p= 0.46), 0 degree (r= 0.09, p= 0.64), 10 degree (r= 0.26, p= 0.18), 15 degree (r= 0.15, p= 0.43), and 20 degree (r= 0.10, p= 0.58) were not associated with quality of life (SF-36 bodily pain, NR: CI for all associations). Hip abductor muscle strength of the operated side at -5 degree (r= 0.003, p= 0.98), 0 degree (r= 0.01, p= 0.94), 10 degree (r= -0.04, p= 0.83), 15 degree (r= -0.003, p= 0.98), and 20 degree (r= 0.04, p= 0.84) were not associated with quality of life (SF-36 general health, NR: CI for all associations). Hip abductor muscle strength of the contralateral side at -5 degree (r= -0.10, p= 0.61), 0 degree (r= -0.14, p= 0.46), 10 degree (r= 0.02, p= 0.91), 15 degree (r= -0.14, p= 0.44), and 20 degree (r= -0.16, p= 0.39) were not associated with quality of life (SF-36 general health, NR: CI for all associations). Hip abductor muscle strength of the operated side at -5 degree (r= 0.25, p= 0.18), 0 degree (r= 0.25, p= 0.19), 10 degree (r= 0.19, p= 0.32), 15 degree (r= 0.21, p= 0.27), and 20 degree (r= 0.28, p= 0.14) were not associated with quality of life (SF-36 vitality, NR: CI for all associations). Hip abductor muscle strength of the contralateral side at -5 degree (r= 0.05, p= 0.76), 0 degree (r= 0.08, p= 0.68), 10 degree (r= 0.10, p= 0.61), 15 degree (r= 0.04, p= 0.82), and 20 degree (r= 0.02, p= 0.90) were not associated with quality of life (SF-36 vitality, NR: CI for all associations). Greater hip abductor muscle strength of the operated side at -5 degree (r= 0.61, p= 0.01), 0 degree (r= 0.58, p= 0.03), 10 degree (r= 0.65, p= 0.01), 15 degree (r= 0.68, p= 0.002), and 20 degree (r= 0.63, p= 0.02) were associated with better quality of life (SF-36 social functioning, NR: CI for all associations). Hip abductor muscle strength of the contralateral side at -5 degree (r= 0.26, p= 0.16), 0 degree (r= 0.23, p= 0.23), and 10 degree (r= 0.25, p= 0.18) were not associated with quality of life (SF-36 social functioning), but greater hip abductor muscle strength of the contralateral side at 15 degree (r= 0.38, p= 0.04) and 20 degree (r= 0.38, p= 0.04) were associated with better quality of life (SF-36 social functioning) (NR: CI for all associations). Hip abductor muscle strength of the operated side at -5 degree (r= 0.23, p= 0.22), 0 degree (r= 0.25, p= 0.19), 10 degree (r= 0.17, p= 0.38), 15 degree (r= 0.21, p= 0.27), and 20 degree (r= 0.35, p= 0.06) were not associated with quality of life (SF-36 role emotional, NR: CI for all associations). Hip abductor muscle strength of the contralateral side at -5 degree (r= -0.001, p= 0.99), 0 degree (r= -0.07, p= 0.70), 10 degree (r= 0.03, p= 0.84), 15 degree (r= 0.06, p= 0.74), and 20 degree (r= 0.05, p= 0.77) were not associated with quality of life (SF-36 role emotional, NR: CI for all associations). Hip abductor muscle strength of the operated side at -5 degree (r= 0.14, p= 0.45), 0 degree (r= 0.20, p= 0.28), 10 degree (r= 0.18, p= 0.35), 15 degree (r= 0.20, p= 0.28), and 20 degree (r= 0.31, p= 0.10) were not associated with quality of life (SF-36 mental health, NR: CI for all associations). Hip abductor muscle strength of the contralateral side at -5 degree (r= 0.006, p= 0.97), 0 degree (r= -0.03, p= 0.87), 10 degree (r= 0.04, p= 0.80), 15 degree (r= 0.05, p= 0.77), and 20 degree (r= 0.05, p= 0.77) were not associated with quality of life (SF-36 mental health, NR: CI for all associations). Shorter time in TUG was associated with better function (Modified HHS (r= -0.57, p= 0.003) and WOMAC (r= -0.48, p= 0.01), NR: CI for all associations). Shorter time in TUG was associated with better quality of life (SF-36 (r= -0.64, p= 0.002), SF-36 physical functioning (r= -0.61, p= 0.001), SF-36 role physical (r= -0.45, p= 0.01), SF-36 bodily pain (r= -0.68, p= 0.00), SF-36 vitality (r= -0.53, p= 0.005), SF-36 social functioning (r= -0.46, p= 0.01), SF-36 role emotional (r= -0.45, p= 0.02), and SF-36 mental health (r= -0.67, p= 0.00)), but it was not associated with quality of life (SF-36 general health (r= 0.15, p= 0.45)) (NR: CI for all associations). |
|  | Yes | SV and LF  February/March 2024 | Moellenbeck et al., (2020) [38] Germany (English) Study design: Prospective cohort longitudinal Funding: The Open Access Publication Fund of the University of Muenster | To compare the habitual sedentary behavior (SB) and physical activity (PA) of older hip osteoarthritis patients before and after elective arthroplasty | Age: 68.88 ± 6.75 N= 16 F (7), M (9)  THA approach: NR  Type: NR  LBP: NR  Health conditions: Cardiovascular, pulmonary, metabolic, gastrointestinal, liver, kidney, blood, cancer, depression, musculoskeletal diseases | Activity level in natural environment: Physical activity:  Sedentary time Sedentary breaks (ActiGraph wGTX3-BT) | Disability: Lequesne Index | Preop Postop: 8.9 months | Longitudinal analyses from preoperatively to postoperatively (SCC): For the 10-min, 20-min and 30-min bouts, decrease in sedentary time was associated with severe preoperative disability, but increase in sedentary time was associated with mild preoperative disability (Lequesne Index, 10-min bouts (rₛ= -0.49, p= 0.02), 20-min bouts (rₛ= -0.59, p= 0.007), 30-min bouts (rₛ= -0.58, p= 0.009) (NR: CI for all associations)). For 60-min bouts, sedentary time was not associated with preoperative disability (Lequesne Index, rₛ= -0.33, p= 0.10, NR: CI).  Increase in time spent in 10-min and 20-min sedentary break were associated with severe preoperative disability while decrease in 10-min and 20-min sedentary break were associated with mild preoperative disability (Lequesne Index, 10-min breaks (rₛ= 0.68, p= 0.002), 20-min breaks (rₛ= 0.47, p= 0.03) (NR: CI for all associations)). For 30-min and 60-min breaks, sedentary break were not associated with preoperative disability (Lequesne Index, rₛ ≤ 0.37, p ≥ 0.07, NR: rₛ, p value, and CI for each association). |
|  | Yes | SV and LF  February/March 2024 | Negrini et al., (2020) [39] Italy (English) Study design: Prospective cohort longitudinal Funding: The Italian Ministry of Health (Ricerca Corrente; project no. L3031; PI: L.Z.) | To investigate the possible predictive role of specific cognitive variables, alone or taken together with other psychological or clinical variables, in the domain of orthopedic postsurgical gait functional recovery | Age: 62.8 ± 7 N= 40 F (22), M (18)  THA approach: Posterolateral  Type: Unilateral LBP: NR Health conditions: NR  Diagnosis was reported as hip OA by the study author (February/March 2024). | Performance-based: TUG | Hip pain: VAS | Preop: 1 day Postop: 12 days | Longitudinal analyses (Multiple regression analyses): TUG at discharge could be predicted by preoperative hip pain (VAS, B= 0.89, CIs= 0.04 to 1.76, standardized ß= 0.29, p= 0.04). |
|  | Yes | SV and RR  February/March 2024 | Ochi et al., (2017) Japan (English) [40]  Study design: Retrospective cohort longitudinal Funding: None | To investigate  the relationship between  preoperative sagittal spinopelvic alignment and postoperative clinical outcomes | Age: 67.5 ± 10.1 N= 92 F (77), M (15)  THA approach: Direct anterior  Type: Unilateral LBP: NR  Health conditions: Spondylolisthesis Compression fracture Degenerative disc disease Osteoarthritis on non-operative side | Spinopelvic Alignment: Sagittal vertical axis  Lumbar lordosis  (X-ray) | Function: Modified HHS function:  HHS gait  HHS functional activities | Preop Postop: 8.5 months | Longitudinal analyses from preoperative to postoperative (Multivariate linear regression): Greater preoperative sagittal vertical axis was associated with worse postoperative function (HHS gait (unadjusted ß-coefficient= -0.36, CI= -0.09 to -0.02, unadjusted p= 0.001, adjusted ß-coefficient= -0.28, CI= -0.08 to -0.007, adjusted p= 0.02) and HHS functional activities (unadjusted ß-coefficient= -0.41, CI= -0.02 to -0.009, unadjusted p= 0.0002, adjusted ß-coefficient= -0.38, CI= -0.02 to -0.005, adjusted p= 0.005)). Greater preoperative lumbar lordosis was associated with better postoperative function (HHS gait, unadjusted ß-coefficient= 0.38, CI= -0.07 to 0.26, unadjusted p= 0.0006, adjusted ß-coefficient= 0.29, CI= 0.03 to 0.22, adjusted p= 0.008), but it was not associated with postoperative function (HHS functional activities, unadjusted ß-coefficient= 0.34, CI= 0.01 to 0.06, unadjusted p= 0.002, adjusted ß-coefficient= 0.23, CI= -0.001 to 0.05, adjusted p= 0.06). |
|  | Yes | SV and LF  July 2024 | Okamoto et al., (2024) [41] Japan (English) Study design: Retrospective cohort longitudinal Funding: The Grants-in-Aid for Scientific Research of Japan Society KAKENHI for the Promotion of Science, Grant Number 21K09239 | To assess  the association between disease processes in hip osteoarthritis and THA outcomes | Age (Rapidly progressive hip OA): 74 ± 6.4 Age (Hip OA): 73 ± 6.4  N (Rapidly progressive hip OA): 25  F (21), M (4)  N (hip OA): 50 F (41), M (9)  THA approach: Direct lateral  Type: Unilateral LBP: Yes Health conditions: NR | Spinopelvic alignment: T1 pelvic angle (T1PA) (X-ray) | LBP: VAS  Hip pain: VAS  QoL: European Quality of Life 5-Dimension 3-Level scale (EQ-5D)  Hip-related health, pain, and function in activities of daily living: HOOS-JR  Satisfaction A validated 4-item questionnaire | Preop Postop timepoint in rapidly progressive hip OA: 42.4 months Postop timepoint in hip OA: 42 months | Longitudinal analyses from preoperative to postoperative (PCC): In rapidly progressive hip OA group, greater preoperative T1PA was associated with higher postoperative LBP (VAS, r= 0.62, CI= 0.30 to 0.82, p < 0.001). However, in hip OA group, preoperative T1PA was not associated with postoperative LBP (VAS, r= 0.04, CI= -0.23 to 0.31, p= 0.78). There were no correlations between T1PA and hip pain, quality of life, hip-related health, pain, and function in activities of daily living, and patient satisfaction in each group (VAS, EQ-5D, HOOS-JR, a validated 4-item questionnaire (NR: r, p value, and CI for all associations)).  Longitudinal analyses from preoperative to postoperative (Linear regression): In rapidly progressive hip OA group, greater preoperative T1PA was associated with higher postoperative LBP (VAS, r²= 0.39, p < 0.05, NR: CI). However, in hip OA group, preoperative T1PA was not associated with postoperative LBP (VAS, r² < 0.01, p > 0.05, NR: CI). |
|  | Yes | SV and RR  July 2024 | Prüfer et al., (2024) [42] Austria (English) Study design: Prospective cohort longitudinal Funding: The research project AMB-REMOB “Ambulante Remobilisation nach Knie und Hüfttotalendoprothesen”, with registration number 305011AXY3, within the cross-border cooperation program Interreg V-A Slovakia–Austria 2014–2020, European Regional Development Fund (ERDF) (partners: Ludwig Boltzmann Institute for Rehabilitation Research, Ludwig Boltzmann Gesellschaft, Vienna; Faculty for Physical Education and Sports, Comenius University in Bratislava). N.Š.’s research activities on this project were partially financially supported by the Slovenian Research Agency through the research program KINSPO—Kinesiology for the Effectiveness and Prevention of Musculoskeletal Injuries in Sports (P5-0443) | To determine the responsiveness of isokinetic dynamometry (ID) in measuring physical function (PF) in THA rehabilitation | Age: 66.6 ± 7.9 N= 8 F (6), M (2)  THA approach: NR Type: NR  LBP: NR Health conditions: NR | Performance-based: SCT 10 MWT at normal speed 10 MWT at fast speed TUG  Impairment-based: Knee extension strength (60deg, 180deg, operated and uninvolved leg) Knee flexion strength (60deg, 180deg, operated and uninvolved leg) (Biodex System 4 Quick-Set™ dynamometer) | Physical function: WOMAC pain WOMAC stiffness WOMAC function WOMAC total Health Assessment Questionnaire Disability Index (HAQ-DI) | Preop: 2 weeks Postop: 10 weeks | Longitudinal analysis from preoperatively to postoperatively (PCC): (Data for THA population provided by the study author (July 2024)) Change in SCT (r= 0.49, p= 0.21), change in 10 MWT at normal speed (r= -0.06, p= 0.88), change in 10 MWT at fast speed (r= -0.36, p= 0.38), change in TUG (r= -0.07, p= 0.87), change in peak torque 60° extension of the operated leg (r= 0.08, p= 0.84), change in peak torque 60° flexion of the operated leg (r= -0.38, p= 0.35), change in peak torque 180° extension of the operated leg (r= 0.09, p= 0.82), change in peak torque 180° flexion of the operated leg (r= -0.03, p= 0.93), change in peak torque 60° extension of the uninvolved leg (r= -0.38, p= 0.34), change in peak torque 60° flexion of the uninvolved leg (r= -0.43, p= 0.28), change in peak torque 180° extension of the uninvolved leg (r= -0.30, p= 0.47), change in peak torque 180° flexion uninvolved leg (r= -0.50, p= 0.20) were not associated with change in pain (WOMAC pain, NR: CI for all associations). Change in SCT (r= 0.51, p= 0.19), change in 10 MWT at normal speed (r= 0.15, p= 0.72), change in 10 MWT at fast speed (r= 0.10, p= 0.81), change in TUG (r= -0.31, p= 0.45), change in peak torque 60° extension of the operated leg (r= -0.12, p= 0.77), change in peak torque 60° flexion of the operated leg (r= -0.56, p= 0.14), change in peak torque 180° extension of the operated leg (r= 0.05, p= 0.90), change in peak torque 180° flexion of the operated leg (r= -0.04, p= 0.92), change in peak torque 60° extension of the uninvolved leg (r= -0.31, p= 0.46), change in peak torque 60° flexion of the uninvolved leg (r= -0.05, p= 0.91), change in peak torque 180° extension of the uninvolved leg (r= -0.27, p= 0.51), change in peak torque 180° flexion uninvolved leg (r= 0.11, p= 0.80) were not associated with change in stiffness (WOMAC stiffness, NR: CI for all associations). Change in SCT (r= 0.61, p= 0.10), change in 10 MWT at normal speed (r= 0.00, p= 0.99), change in 10 MWT at fast speed (r= -0.15, p= 0.72), change in TUG (r= -0.27, p= 0.51), change in peak torque 60° extension of the operated leg (r= -0.05, p= 0.90), change in peak torque 60° flexion of the operated leg (r= -0.57, p= 0.14), change in peak torque 180° extension of the operated leg (r= 0.03, p= 0.95), change in peak torque 180° flexion of the operated leg (r= -0.06, p= 0.88), change in peak torque 60° extension of the uninvolved leg (r= -0.41, p= 0.31), change in peak torque 60° flexion of the uninvolved leg (r= -0.23, p= 0.59), change in peak torque 180° extension of the uninvolved leg (r= -0.36, p= 0.38), change in peak torque 180° flexion uninvolved leg (r= -0.16, p= 0.71) were not associated with change in function (WOMAC function, NR: CI for all associations). Change in SCT (r= 0.61, p= 0.10), change in 10 MWT at normal speed (r= 0.04, p= 0.93), change in 10 MWT at fast speed (r= -0.12, p= 0.77), change in TUG (r= -0.28, p= 0.50), change in peak torque 60° extension of the operated leg (r= -0.05, p= 0.89), change in peak torque 60° flexion of the operated leg (r= -0.59, p= 0.12), change in peak torque 180° extension of the operated leg (r= 0.05, p= 0.91), change in peak torque 180° flexion of the operated leg (r= -0.06, p= 0.88), change in peak torque 60° extension of the uninvolved leg (r= -0.40, p= 0.32), change in peak torque 60° flexion of the uninvolved leg (r= -0.23, p= 0.58), change in peak torque 180° extension of the uninvolved leg (r= -0.35, p= 0.40), change in peak torque 180° flexion uninvolved leg (r= -0.15, p= 0.72) were not associated with change in physical function (WOMAC total, NR: CI for all associations). Change in SCT (r= -0.08, p= 0.86), change in 10 MWT at normal speed (r= -0.18, p= 0.69), change in 10 MWT at fast speed (r= -0.39, p= 0.38), change in TUG (r= 0.31, p= 0.49), change in peak torque 60° extension of the operated leg (r= -0.02, p= 0.97), change in peak torque 60° flexion of the operated leg (r= 0.52, p= 0.23), change in peak torque 180° extension of the operated leg (r= -0.30, p= 0.51), change in peak torque 180° flexion of the operated leg (r= 0.44, p= 0.32), change in peak torque 60° extension of the uninvolved leg (r= -0.24, p= 0.60), change in peak torque 60° flexion of the uninvolved leg (r= 0.19, p= 0.68), change in peak torque 180° extension of the uninvolved leg (r= -0.31, p= 0.49), change in peak torque 180° flexion uninvolved leg (r= -0.28, p= 0.54) were not associated with change in physical function (HAQ-DI, NR: CI for all associations). |
|  | Yes | SV and LF  February/March 2024 | Qiu et al., (2014) [43]  China (Chinese) Study design: Cross-sectional and cohort longitudinal Funding: The Innovation and Entrepreneurship Training Projects in College Students of Guangdong Province, No. 1056012088 | To explore the predictive effects of preoperative subjective functional assessments on patients' activity function six months after hip arthroplasty | Age: 68.42 ± 6.31 N= 36 F (16), M (20)  THA approach: Lateral  Type: Unilateral LBP: NR  Health conditions: NR | Performance-based: 6MWT TUG | Function: The Amsterdam Longitudinal Aging Study Physical Activity Questionnaire (LAPAQ) SF-36 | Preop: 2 weeks Postop: 6 months | Cross-sectional analyses postoperatively (PCC):  Better performance in 6MWT was associated with better function (LAPAQ, r²= 0.76, NR: r, p value, and CI). Shorter time in TUG was associated with better function (LAPAQ, r²= 0.60, NR: r, p value, and CI).  Longitudinal analysis from preoperatively to postoperatively (PCC): Better performance in 6MWT at 6 months postoperatively was associated with better preoperative function (LAPAQ, r²= 0.84, NR: r, p value, and CI). Shorter time in TUG at 6 months postoperatively was associated with better preoperative function (LAPAQ, r²= 0.66, NR: r, p value, and CI). Better performance in 6MWT and shorter time in TUG postoperatively were associated with better preoperative function (SF-36, NR: r, p value, and CI for all associations). |
|  | Yes | SV and LF  February/March 2024 | Segev−Jacubovski (2023) [44] Israel (English) Study design: Cross-sectional Funding: None | To determine which physical factors at admission would predict functional ability at discharge | Age: 76.20 ± 6.09 N= 30 F (21), M (9)  THA approach: NR  Type: Unilateral LBP: NR  Health  conditions: NR | Impairment-based: Hand−grip strength (Hand dynamometer) | Positive feeling: Positive affect questionnaire  Depression: Geriatric depression scale (GDS)  Hip pain: NRS | Postop: 5.23 days | Cross-sectional analyses postoperatively (PCC):  Greater hand−grip strength was associated with both greater positive feeling (Positive affect questionnaire, r= 0.43, p < 0.05) and lower depression (GDS, r= -0.34, p < 0.05), but it was not associated with hip pain (NRS, r= -0.21 p > 0.05) (NR: CI for all associations). |
|  | Yes | SV and RR  February/March 2024 | Sliwinski et al., (2006) [45] USA (English) Study design: Cross-sectional  Funding: NR | To evaluate the relationships  between temporal spatial gait parameters and SF-36 QoL sub scales after THA | Age: 70.9 ± 4.2  N= 16 F (9), M (7)  THA approach: NR Type: Unilateral LBP: NR  Health conditions: NR | Impairment-based: Gait parameters: Velocity Double Support Cadence Stride Length Single Support (Vicon Motion Analysis System) | QoL: SF-36: Physical Functioning SF-36: Role Physical  SF-36: Bodily Pain SF-36: General Health SF-36: Vitality SF-36: Social Functioning SF-36: Role Emotional  SF-36: Mental Health | Postop: From 2 months to 2 years | Cross-sectional analyses postoperatively (Pearson Product-Moment Correlations): Velocity was not associated with quality of life (Physical functioning (r= 0.45), role physical (r= 0.46), bodily pain (r= 0.50), general health (r= 0.59), vitality (r= 57), social functioning (r= 0.31), role emotional (r= 0.09), and mental health (r= 0.50)) (p > 0.01 and NR: CI for all associations). Double support was not associated with quality of life (Physical functioning (r= -0.40), role physical (r= -0.50), bodily pain (r= 0.55), general health (r= -0.50), vitality (r= 0.61), social functioning (r= 0.42), role emotional (r= -0.09), and mental health (r= -0.43)) (p > 0.01 and NR: CI for all associations). Cadence was not associated with quality of life (Physical functioning (r= 0.44), role physical (r= 0.42), bodily pain (r= 0.41), general health (r= 0.61), vitality (r= 0.30), social functioning (r= 0.30), role emotional (r= -0.33), and mental health (r= 0.27)) (p > 0.01 and NR: CI for all associations). Stride length was not associated with quality of life (Physical functioning (r= 0.31), role physical (r= 0.30), bodily pain (r= 0.37), general health (r= 0.52), vitality (r= 0.31), social functioning (r= 0.16), role emotional (r= 0.44), and mental health (r= 0.47)) (p > 0.01 and NR: CI for all associations). Single support was not associated with quality of life (Physical functioning (r= -0.36), role physical (r= -0.20), bodily pain (r= -0.10), general health (r= -0.25), vitality (r= -0.50), social functioning (r= -0.21), role emotional (r= 0.46), and mental health (r= -0.05)) (p > 0.01 and NR: CI for all associations). |
|  | Yes | SV and RR  February/March 2024 | Tang et al., (2021)  USA (English) [46]  Study design: Cross-sectional and prospective cohort longitudinal  Funding: NR | To evaluate and  compare the level of objective physical activity and sleep  measures using accelerometry data to subjective reported  clinical outcomes in patients before and after THA | Age: 61.6 ± 10.2  N (Whole population) = 41  F (24), M (17)  N (Population completed assessment at 1-2 weeks) = 30  N (Population completed assessment at 3 months) = 20  THA approach:  Anterior (18), posterior (18), and superior (5)  Type: NR  LBP: NR  Health conditions: NR | Activity level in natural environment:  Daily steps  (FitBit Flex) | Clinical outcome:  HOOS JR | Preop  Postop:  1-2 weeks  3 months | Cross-sectional analysis at 2 weeks postop (PCC):  Average daily steps were not associated with clinical outcome (HOOS JR, r= -0.24, p= 0.33, NR: CI).  Cross-sectional analysis at 3 months postop (PCC):  Average daily steps were not associated with clinical outcome (HOOS JR, r= -0.40, p= 0.27, NR: CI).  Longitudinal analysis from preoperative to postoperative (PCC):  Change in average daily steps were not associated with change in clinical outcome (HOOS JR, r= 0.12, p= 0.61, NR: CI). |
|  | Yes | SV and RR  February/March 2024 | Tolk et al., (2019) [47] Netherlands (English) Study design: Prospective cohort longitudinal Funding: None | To evaluate responsiveness after THA of the OARSI recommended performance-based measures, for measurement of physical function in patients with severe hip OA | Age (Whole population): 69 ± 9.5 N (Whole population) = 90 F (61), M (29)  N (Population for responsiveness) = 77  F and M: NR THA approach: NR Type: Unilateral LBP: NR Health conditions: NR | Performance-based: 30-s CST 40 m FPWT 10-step SCT | Physical function: HOOS physical function (HOOS—PS) OHS 7-point Likert  scale for change in activities of daily living   Pain: NRS pain | Preop Postop: 12 months | Longitudinal analyses from preoperative to postoperative (SCC): Change in 30-s CST was not associated with change in physical function (HOOS—PS (rₛ= 0.30), OHS (rₛ= 0.23), and 7-point Likert scale for change in activities of daily living (rₛ= 0.37)) and change in pain (NRS pain, rₛ= -0.04) (NR: p value and CI for all associations). Change in 40 m FPWT was not associated with change in physical function (HOOS—PS (rₛ= 0.21), OHS (rₛ= 0.27), and 7-point Likert scale for change in activities of daily living (rₛ= 0.28)) and change in pain (NRS pain, rₛ= -0.13) (NR: p value and CI for all associations). Change in 10-step SCT was not associated with change in physical function (HOOS—PS (rₛ= -0.35), OHS (rₛ= -0.26) and 7-point Likert scale for change in activities of daily living (rₛ= -0.18)) and change in pain (NRS pain, r= 0.14) (NR: p value and CI for all associations). |
|  | Yes | SV and LF  February/March 2024 | Tsukagoshi et al., (2015) [48] Japan (English) Study design: Cross-sectional Funding: A research fellowship award from the Japan Society for the Promotion of Science for Young Scientists (23 6521) | To investigate the relationship between functional impairments and peak hip extension angle during gait in THA patients and to identify the factors influencing PHEA during gait | Age: 61.9 ± 6.7 N= 67 F (67), M (0)  THA approach: Anterolateral  Type: Unilateral (n=45) or bilateral (n=22) LBP: NR  Health conditions: NR | Impairment-based: Peak hip extension angle (PHEA) (A six-camera Vicon motion system) | Hip pain: VAS | Postop: 50.3 months | Cross-sectional analyses postoperatively (Pearson's or Spearman's correlation coefficient) Greater PHEA was associated with lower pain (VAS, r= -0.26, p= 0.03, NR: CI).  Cross-sectional analyses postoperatively (Stepwise multiple regression): In regression model with pain and hip abductor torque as independent variables and PHEA as dependent variable, greater PHEA was associated with lower pain (VAS, unstandardized B= -0.42, standardized β= -0.37, p= 0.00, NR: CI). In regression model with pain, hip abductor torque, and hip extension ROM as independent variables and PHEA as dependent variable, greater PHEA was associated with lower pain (VAS, unstandardized B= -0.40, standardized β= -0.35, p= 0.001, NR: CI). |
|  | Yes | SV and RR  February/March 2024 | Tugay et al., (2004) [49]  Turkey (English) Study design: Cross-sectional and cohort longitudinal Funding: NR | To investigate  the relationship between independence level in functional activities and pain in the early postoperative period in patients with THA | Age: 52.46 ± 13.31 N= 26 F (23), M (3)  THA approach: Posterolateral Type: Unilateral LBP: NR  Health conditions: NR | Impairment-based:  Ambulation speed  (Iowa Ambulation Speed Scale (IASS)) | Pain: VAS | Postop: 2 and 6 days | Cross-sectional analyses on sixth day (PCC): Ambulation speed was not associated with pain (VAS, r= 0.15, p= 0.45, NR: CI).  Longitudinal analyses postoperatively (PCC): Ambulation speed on sixth day was not associated with pain on second day (VAS, r= 0.07, p= 0.73, NR: CI). |
|  | Yes | SV and RR  July 2024 | Vergari et al., (2024) [50] Japan (English) Study design: Prospective cohort longitudinal  Funding: The Japan Society for the Promotion of Science (JSPS) through an invitational fellowship grant (S21026) | To investigate the relationship between hip, pelvis and lumbar spine mobility and alignment before and after THA with quality of life | Age: 66 (NR: SD) N (Preoperative group) = 70 F (13), M (57)  N (6 months postoperative group) = 62 F (12), M (50)  THA approach: NR Type: NR LBP: NR  Health conditions: NR | Spinopelvic alignment: T1 pelvic angle (T1PA) Lumbar lordosis (X−ray) | QoL: Japanese Orthopaedic Association Hip Disease Assessment Questionnaire (JHEQ) JHEQ mental  JHEQ activity  JHEQ pain | Preop Postop: 6 months | Longitudinal analysis from preoperatively to postoperatively (Multivariate regression analysis): In standing position, lower preoperative T1PA was associated with improvement in quality of life (JHEQ (R= -0.37, p < 0.01, NR: CI), JHEQ mental (R= -0.38, p < 0.01, NR: CI), and JHEQ activity (R= -0.44, p < 0.05, NR: CI), but it was not associated with improvement in quality of life (JHEQ pain, NR: R, p value, and CI). In standing position, preoperative lumbar lordosis was not associated with improvement in quality of life (JHEQ, JHEQ mental, JHEQ activity, and JHEQ pain (NR: R, p value, and CI for all associations)). In extension position, preoperative T1PA and preoperative lumbar lordosis were not associated with improvement in quality of life (JHEQ, JHEQ mental, JHEQ activity, and JHEQ pain (NR: R, p value, and CI for all associations)). |
|  | Yes | SV and RR  February/March 2024 | Wada et al., (2019) [51] Japan (English) Study design: Prospective cohort longitudinal  Funding: NR | To examine the relationships  between changes in gait variability and changes in pain level before and after THA | Age: 61.0 ± 7.1 N= 23 F (23), M (0)  THA approach: Anterolateral or anterior  Type: Unilateral LBP: NR Health conditions: NR | Impairment-based: Gait variability:  Stride time (coefficient of variation: CV) Trunk movement variability:  Harmonic ratio (HR) in anteroposterior direction (AP) HR in vertical direction (VT) HR in mediolateral direction (ML) (Accelerometer) | Hip pain: A scale from 0 to 10 | Preop: 1 month Postop: 12 months | Longitudinal analyses from preoperative to postoperative (SCC): Greater change in CV was associated with greater change in hip pain (A scale from 0 to 10, rₛ= 0.54, p= 0.008, NR: CI). Change in HR in the AP direction (rₛ= -0.05), in the VT direction (rₛ= -0.05), and in the ML direction (rₛ= -0.11) were not associated with change in hip pain (A scale from 0 to 10, NR: p value and CI for all associations). |
|  | Yes | SV and LF  July 2024 | Wagenmakers et al., (2008) [52] Netherlands (English) Study design: Cross-sectional Funding: NR | To assess the reliability and validity of the scores on the Short Questionnaire to Assess Health-enhancing physical activity (SQUASH) as a measure of the physical activity behavior in the general Dutch adult population after THA | Age: 70 ± 8 N= 39 F (24), M (15)  THA approach: NR Type: NR  LBP: NR Health conditions: NR Diagnosis was reported as hip OA by the study author (July 2024). | Activity level in natural environment: Physical activity (Mean counts per minute) Total physical activity Light intensity activity Moderate intensity activity Vigorous intensity activity (Acti-Graph™ GT1M activity monitor) | Physical activity: SQUASH | PROMs: Postop: 1 year 1 year and 3.7 weeks  Activity monitor: Postop: During the two weeks following the completion of the questionnaire at second timepoint | Cross-sectional analysis postoperatively (SCC): Greater physical activity (mean counts per minute, Activity monitor) was associated with greater total physical activity (SQUASH, rₛ= 0.67, p= 0.01, NR: CI).  Greater total physical activity (Activity monitor) was associated with greater total physical activity (SQUASH, rₛ= 0.56, p= 0.01, NR: CI). Time spent in light intensity activities and time spent in moderate intensity activities (Activity monitor) were not associated with time spent in light intensity activities (SQUASH, rₛ= 0.20, p= 0.22) and time spent in moderate intensity activities (SQUASH, rₛ= 0.40, p= 0.40) (NR: CI for all associations). Greater time spent in vigorous intensity activities (Activity monitor) was associated with greater time spent in vigorous intensity activities (SQUASH, rₛ= 0.35, p= 0.03) (NR: CI for all associations). |
|  | Yes | SV and LF  February/March 2024 | Yamaguchi et al., (2019) [53] Japan (Japanese) Study design: Cohort longitudinal Funding: NR | To examine the relationship between the change in the number of steps from discharge to one-month post-discharge and changes in physical function, pain, and self-efficacy | Age: 64.7 ± 7.5 N= 20 F (20), M (0)  THA approach: Anterolateral  Type: Unilateral LBP: NR  Health conditions: Hypertension Hyperlipidemia | Activity level in natural environment: Daily steps  (Accelerometer) | Fear of falling: Modified Falls Efficacy Scale (MFES)  Hip pain: VAS | Postop: At discharge 1-month post-discharge | Longitudinal analyses from discharge to one month post-discharge (SCC): Greater change in daily steps was associated with greater change in fear of falling (MFES, rₛ= 0.62, p < 0.01, NR: CI), but it was not associated with change in pain (VAS, rₛ= -0.10, NR: p value and CI). |

PROMs: Patient−reported outcome measure, OA: Osteoarthritis, THA: Total hip arthroplasty, THR: Total hip replacement, PCC: Pearson’s correlation coefficients, SCC: Spearman’s correlation coefficients, NR: Not reported, SD: Standard deviation, Preop: Preoperative, Postop: Postoperative, VAS: Visual analog scale, LBP: Low back pain, ROM: Range of motion, 6MWT: Six-Minute Walk Test, TUG: Timed-Up-and-Go, 30-s CST: 30 second Chair Stand Test, 40 m FPWT: 40 meter fast-paced walk test, SCT: Stair Climb Test, 10 MWT: Ten-Meter Walk Test, PA: Physical activity, HHS: Harris Hip Score, HOOS: Hip Disability and Osteoarthritis Outcome Score, HOOS JR: Hip Disability and Osteoarthritis Outcome Score Joint Replacement, ADL: activities of daily living, PROMIS: Patient Reported Outcomes Measurement Information System, WOMAC: The Western Ontario and McMaster Universities Osteoarthritis Index, WOMAC-PF: WOMAC-Physical function, SF-36: The 36-Item Short Form Survey, SF-8: Short Form-8, OHS: Oxford Hip Score, NRS: Numeric Rating Scale, UCLA: University of California Los Angeles, LEFS: Lower Extremity Functional Scale, SQUASH: Short Questionnaire to Assess Health-enhancing physical activity, ODI: Oswestry Disability Index, QoL: Quality of Life, PROMs: Patient reported outcome measures, FJS-12: 12-item Forgotten Joint Score, HOOS-PS: The disability and Osteoarthritis Outcome Score - Physical Function Short Form, SF36- PF: The 36-tem Short-Form- Physical Functioning

**References**

1. Luna I, Kehlet H, Peterson B, Wede HR, Hoevsgaard S, Aasvang EK. Early patient-reported outcomes versus objective function after total hip and knee arthroplasty: a prospective cohort study. The bone & joint journal. 2017;99(9):1167-75.

2. Luna IE, Kehlet H, Wede HR, Hoevsgaard SJ, Aasvang EK. Objectively measured early physical activity after total hip or knee arthroplasty. Journal of clinical monitoring and computing. 2019;33(3):509-22. doi: <https://dx.doi.org/10.1007/s10877-018-0185-5>.

3. Heiberg KE, Ekeland A, Bruun-Olsen V, Mengshoel AM. Recovery and prediction of physical functioning outcomes during the first year after total hip arthroplasty. Archives of physical medicine and rehabilitation. 2013;94(7):1352-9. doi: <https://dx.doi.org/10.1016/j.apmr.2013.01.017>.

4. Heiberg KE. RECOVERY OF PHYSICAL FUNCTIONING AFTER TOTAL HIP ARTHROPLASTY University of Oslo; 2013.

5. Abujaber SB. the relationships between physical impairments, functional limitations and movement asymmetries before and after total hip arthroplasty: A Longitudinal Study: University of Delaware; 2014.

6. Biggs P, Holsgaard-Larsen A, Holt CA, Naili JE. Gait function improvements, using Cardiff Classifier, are related to patient-reported function and pain following hip arthroplasty. Journal of orthopaedic research : official publication of the Orthopaedic Research Society. 2022;40(5):1182-93. doi: <https://dx.doi.org/10.1002/jor.25149>.

7. Boardman D, Dorey F, Thomas B, Lieberman J. The accuracy of assessing total hip arthroplasty outcomes: a prospective correlation study of walking ability and 2 validated measurement devices. The Journal of arthroplasty. 2000;15(2):200-4.

8. Bolink S, Lenguerrand E, Brunton L, Wylde V, Gooberman-Hill R, Heyligers I, et al. Assessment of physical function following total hip arthroplasty: inertial sensor based gait analysis is supplementary to patient-reported outcome measures. Clinical Biomechanics. 2016;32:171-9.

9. Cao B, Li X, Lu Z, Liang J, He L. Anatomical changes in lumbosacral vertebrae and their correlation with facet joint-derived low back pain in patients with hip osteoarthritis after total hip arthroplasty: a cohort study. Annals of Translational Medicine. 2022;10(7).

10. Casartelli NC, Bolszak S, Impellizzeri FM, Maffiuletti NA. Reproducibility and validity of the physical activity scale for the elderly (PASE) questionnaire in patients after total hip arthroplasty. Physical therapy. 2015;95(1):86-94. doi: <https://dx.doi.org/10.2522/ptj.20130557>.

11. Cinnamon CC, Longworth JA, Brunner JH, Chau VK, Ryan CA, Dapiton KR, et al. Static and dynamic abductor function are both associated with physical function 1 to 5 years after total hip arthroplasty. Clin Biomech. 2019;67:127-33. doi: 10.1016/j.clinbiomech.2019.05.009.

12. Davis KE, Ritter MA, Berend ME, Meding JB. The importance of range of motion after total hip arthroplasty. Clinical Orthopaedics and Related Research®. 2007;465:180-4.

13. Dayton MR, Judd DL, Hogan CA, Stevens-Lapsley JE. Performance-Based Versus Self-Reported Outcomes Using the Hip Disability and Osteoarthritis Outcome Score After Total Hip Arthroplasty. American journal of physical medicine & rehabilitation. 2016;95(2):132-8. doi: <https://dx.doi.org/10.1097/PHM.0000000000000357>.

14. Eyvazov K, Eyvazov B, Basar S, Nasto LA, Kanatli U. Effects of total hip arthroplasty on spinal sagittal alignment and static balance: a prospective study on 28 patients. European spine journal : official publication of the European Spine Society, the European Spinal Deformity Society, and the European Section of the Cervical Spine Research Society. 2016;25(11):3615-21.

15. Fallahzadeh R, Verdonk F, Ganio E, Culos A, Stanley N, Maric I, et al. Objective Activity Parameters Track Patient-specific Physical Recovery Trajectories After Surgery and Link With Individual Preoperative Immune States. Ann Surg. 2023;277(3):E503-E12. doi: 10.1097/SLA.0000000000005250.

16. Foucher KC, Thorp LE, Orozco D, Hildebrand M, Wimmer MA. Differences in preferred walking speeds in a gait laboratory compared with the real world after total hip replacement. Archives of physical medicine and rehabilitation. 2010;91(9):1390-5.

17. Foucher KC, Cinnamon CC, Ryan CA, Chmell SJ, Dapiton K. Hip abductor strength and fatigue are associated with activity levels more than 1 year after total hip replacement. Journal of Orthopaedic Research®. 2018;36(5):1519-25.

18. Fujita K, Makimoto K, Tanaka R, Mawatari M, Hotokebuchi T. Prospective study of physical activity and quality of life in Japanese women undergoing total hip arthroplasty. Journal of orthopaedic science : official journal of the Japanese Orthopaedic Association. 2013;18(1):45-53. doi: <https://dx.doi.org/10.1007/s00776-012-0318-5>.

19. Fujita T, Hamai S, Shiomoto K, Okazawa K, Nasu Y-k, Hara D, et al. Analysis of factors influencing patient satisfaction after total hip arthroplasty in a Japanese cohort: the significant effect of postoperative physical activity. Journal of Physical Therapy Science. 2022;34(2):76-84.

20. Goeb YL, Krell EC, Nguyen JT, Carroll KM, Jerabek SA, Mayman DJ, et al. Early recovery outcomes in patients undergoing total hip arthroplasty through a posterior approach with modified postoperative precautions. The Journal of Arthroplasty. 2021;36(8):2817-22.

21. Harada S, Hamai S, Shiomoto K, Kawahara S, Hara D, Harada T, Nakashima Y. Predictors of physical activity recovery after total hip arthroplasty: a prospective observational study. International Orthopaedics. 2024;48(3):753-60.

22. Holm B, Thorborg K, Husted H, Kehlet H, Bandholm T. Surgery-induced changes and early recovery of hip-muscle strength, leg-press power, and functional performance after fast-track total hip arthroplasty: a prospective cohort study. PloS one. 2013;8(4):e62109.

23. Holstege MS, Lindeboom R, Lucas C. Preoperative quadriceps strength as a predictor for short-term functional outcome after total hip replacement. Archives of physical medicine and rehabilitation. 2011;92(2):236-41. doi: <https://dx.doi.org/10.1016/j.apmr.2010.10.015>.

24. Huang C-H, Foucher KC. Step Length Asymmetry and Its Associations With Mechanical Energy Exchange, Function, and Fatigue After Total Hip Replacement. Journal of orthopaedic research : official publication of the Orthopaedic Research Society. 2019;37(7):1563-70. doi: <https://dx.doi.org/10.1002/jor.24296>.

25. Jelsma J, Van Kuijk S, Buil I, Heyligers I, Grimm B, Schotanus M. Only limited correlations between patient-reported outcomes and objectively monitored physical activity 10-years after THA. Acta Orthopædica Belgica. 2021;87:593-9.

26. Kamimura A, Sakakima H, Tsutsumi F, Sunahara N. Preoperative predictors of ambulation ability at different time points after total hip arthroplasty in patients with osteoarthritis. Rehabilitation research and practice. 2014.

27. Kaufmann M, Nüesch C, Clauss M, Pagenstert G, Eckardt A, Ilchmann T, et al. Functional assessment of total hip arthroplasty using inertial measurement units: Improvement in gait kinematics and association with patient‐reported outcome measures. Journal of Orthopaedic Research®. 2023;41(4):759-70.

28. Kirschner J, Michel S, Becker R, Stiebitz O, Hommel H, Schulz R, et al. Determination of Relationships between Symmetry-Based, Performance-Based, and Functional Outcome Measures in Patients Undergoing Total Hip Arthroplasty. Journal of Personalized Medicine. 2023;13(7):1046.

29. Kobayashi D, Choe H, Kobayashi N, Watanabe S, Inaba Y. Effects of changes in whole-body alignment on ipsilateral knee pain after total hip arthroplasty. Journal of Orthopaedic Science. 2023;28(2):398-402.

30. Lin X, Wu W, Weijer RHA, Prins MR, van Dieen JH, Bruijn SM, Meijer OG. Strong relationship of muscle force and fall efficacy, but not of gait kinematics, with number of falls in the year after Total Hip Arthroplasty for osteoarthritis: An exploratory study. Clinical biomechanics (Bristol, Avon). 2022;92(8611877, dgl):105551. doi: <https://dx.doi.org/10.1016/j.clinbiomech.2021.105551>.

31. Lindemann U, Becker C, Unnewehr I, Muche R, Aminin K, Dejnabadi H, et al. Gait analysis and WOMAC are complementary in assessing functional outcome in total hip replacement. Clinical rehabilitation. 2006;20(5):413-20.

32. Lyman S, Hidaka C, Fields K, Islam W, Mayman D. Monitoring patient recovery after THA or TKA using mobile technology. HSS Journal®. 2020;16(2_suppl):358-65.

33. Mahmood SS, Mukka SS, Crnalic S, Wretenberg P, Sayed-Noor AS. Association between changes in global femoral offset after total hip arthroplasty and function, quality of life, and abductor muscle strength. Acta Orthop. 2016;87(1):36-41. doi: 10.3109/17453674.2015.1091955.

34. Mark‐Christensen T, Kehlet H. Assessment of functional recovery after total hip and knee arthroplasty: An observational study of 95 patients. Musculoskeletal Care. 2019;17(4):300-12.

35. McMeeken JM, Galea MP. Impairment of muscle performance before and following total hip replacement. International Journal of Therapy and Rehabilitation. 2007;14(2):55-62.

36. Meessen JMTA, Fiocco M, Tordoir RL, Sjer A, Verdegaal SHM, Slagboom PE, et al. Association of handgrip strength with patient-reported outcome measures after total hip and knee arthroplasty. Rheumatology international. 2020;40(4):565-71. doi: <https://dx.doi.org/10.1007/s00296-020-04532-5>.

37. Melchiorri G, Viero V, Triossi T, Sorge R, Marchetti C, Arena NE, Tancredi V. Late isometric assessment of hip abductor muscle and its relationship with functional tests in elderly women undergoing replacement of unilateral hip joint. American Journal of Physical Medicine & Rehabilitation. 2015;94(10):758-67.

38. Moellenbeck B, Horst F, Gosheger G, Theil C, Seeber L, Kalisch T, editors. Sedentary behavior in older patients before and after total hip arthroplasty: a prospective cohort study. Healthcare; 2020: MDPI.

39. Negrini F, Preti M, Zirone E, Mazziotti D, Biffi M, Pelosi C, et al. The Importance of Cognitive Executive Functions in Gait Recovery After Total Hip Arthroplasty. Arch Phys Med Rehabil. 2020;101(4):579-86. doi: 10.1016/j.apmr.2019.12.004.

40. Ochi H, Homma Y, Baba T, Nojiri H, Matsumoto M, Kaneko K. Sagittal spinopelvic alignment predicts hip function after total hip arthroplasty. Gait Posture. 2017;52:293-300. doi: 10.1016/j.gaitpost.2016.12.010.

41. Okamoto Y, Wakama H, Nakamura K, Ishitani T, Otsuki S, Neo M. Worse Patient-Reported Outcomes and Spino-Pelvic Parameters After Total Hip Arthroplasty for Rapidly Progressive Osteoarthritis of the Hip Compared to Osteoarthritis: A Propensity-Matched Cohort Study. The Journal of Arthroplasty. 2024.

42. Prüfer F, Pavlović M, Matko Š, Löfler S, Fischer MJ, Šarabon N, Grote V, editors. Responsiveness of Isokinetic Dynamometry in Patients with Osteoarthritis after Knee and Hip Arthroplasty: A Prospective Repeated-Measures Cohort Study. Healthcare; 2024: MDPI.

43. Qiu SM, Chen XP, Zheng DZ, Lin YB, Lin J, Ma HL, Zeng RM. Preoperative prediction of early physical function in elder patients undergoing hip arthroplasty using a subjective physical activity questionnaire. Chin J Tissue Eng Res. 2014;18(4):517-22. doi: 10.3969/j.issn.2095-4344.2014.04.005.

44. Segev-Jacubovski O. Functional ability, psychological factors, and rehabilitation outcomes after elective total hip replacement. CANADIAN JOURNAL OF OCCUPATIONAL THERAPY-REVUE CANADIENNE D ERGOTHERAPIE. 2023. doi: 10.1177/00084174231168018. PubMed PMID: WOS:000972450900001.

45. Sliwinski M, Sisto S. Gait, quality of life, and their association following total hip arthroplasty. Journal of geriatric physical therapy (2001). 2006;29(1):10-7.

46. Tang A, Behery OA, Singh V, Yeroushalmi D, Davidovitch R, Schwarzkopf R. Do physical activity and sleep correlate with patient-reported outcomes in total hip arthroplasty? The Journal of Hip Surgery. 2021;5(02):047-54.

47. Tolk JJ, Janssen RP, Prinsen CA, van der Steen MC, Bierma Zeinstra SM, Reijman M. Measurement properties of the OARSI core set of performance-based measures for hip osteoarthritis: a prospective cohort study on reliability, construct validity and responsiveness in 90 hip osteoarthritis patients. Acta orthopaedica. 2019;90(1):15-20.

48. Tsukagoshi R, Tateuchi H, Fukumoto Y, Akiyama H, So K, Kuroda Y, et al. Factors associated with restricted hip extension during gait in women after total hip arthroplasty. Hip International. 2015;25(6):543-8.

49. Tugay N, Akarcali I, Kaya D, Tugay BU, Atilla B, Tokgozoglu AM. High independence level in functional activities reduces hospital stay after total hip arthroplasty regardless of pain intensity. Saudi medical journal. 2004;25(10):1382-7.

50. Vergari C, Kim Y, Takemoto M, Tokuyasu H, Shimizu Y, Tanaka C, et al. The relationship between spino-pelvic-hip mobility and quality of life before and after total hip arthroplasty. Archives of Orthopaedic and Trauma Surgery. 2024;144(3):1379-87.

51. Wada O, Asai T, Hiyama Y, Nitta S, Mizuno K. Gait Variability in Women With Hip Osteoarthritis Before and After Total Hip Replacement A Prospective Cohort Study. AMERICAN JOURNAL OF PHYSICAL MEDICINE & REHABILITATION. 2019;98(10):866-71. doi: 10.1097/PHM.0000000000001206. PubMed PMID: WOS:000505947300011.

52. Wagenmakers R, Akker-Scheek Ivd, Groothoff JW, Zijlstra W, Bulstra SK, Kootstra JW, et al. Reliability and validity of the short questionnaire to assess health-enhancing physical activity (SQUASH) in patients after total hip arthroplasty. BMC Musculoskeletal Disorders. 2008;9:1-9.

53. 山口智也, 矢倉千昭, 加藤木丈英, 白井智裕, 岸田俊二. 人工股関節全置換術術後患者における退院時から退院後 1 ヵ月の歩数変化量と身体機能, 疼痛, 自己効力感との関係. 理学療法科学. 2019;34(6):743-8.
