# Supplementary material for: Association between physical measures of spinopelvic alignment and physical functioning with patient reported outcome measures after total hip arthroplasty: Systematic review and narrative synthesis
Source: PLoS One. 2025 Dec 29;20(12):e0339615. doi: 10.1371/journal.pone.0339615 (PMC12747333; doi:10.1371/journal.pone.0339615)
Supplement: Attachment — Submitted filename: Response to reviewers.docx [file pone.0339615.s007.docx]

| **Reviewers’ comments (reviewer 1)** | **Response to reviewers** |
| --- | --- |
| Reviewer #1: Thank you for the opportunity to review this systematic review on the association between physical measures—especially spinopelvic alignment—and patient-reported outcomes after total hip arthroplasty (THA). The paper is well-written, thorough, and addresses a very relevant and emerging topic in joint arthroplasty. | Thank you so much for your feedback on the content and topic of the systematic review. It’s encouraging to know that the time we invested in developing the topic and conducting the study was worthwhile. |
| The title highlights spinopelvic alignment, but only six of the included studies actually looked at spinopelvic measures. This might be a bit confusing for readers who expect a more focused synthesis on that topic. It would be helpful if the authors could discuss this in the Introduction and/or Discussion. Was this due to a true lack of studies in the field? Or were there strict inclusion criteria that limited the number of relevant studies? Either way, a short explanation would help set expectations. | Thank you for your valuable feedback. Studying the relationship between physical measures of spinopelvic alignment and PROMs was one of the main focuses of this study, developed based on the scoping search, through which we defined the study objectives. Our inclusion criteria required parameters to include one point on the spine and one on the pelvis to focus exclusively on the spinopelvic region. This resulted in six included studies, representing the total available based on our definition.  We have incorporated the following context into the introduction to set clear expectations for the reader upfront, which we believe addresses your concern about potential confusion before reaching the results or discussion sections:  -Line 73-77: *“Although interest in spinopelvic alignment and THA outcomes is growing, systematic reviews focusing specifically on physical measures linking spinal and pelvic reference points remain limited. To address this gap, we applied strict inclusion criteria, requiring measures with one point on the spine and one on the pelvis, resulting in a smaller but more targeted pool of studies, further discussed in the review.”*  We also added the following explanation in the discussion section:  - Line 514-518: *“Only six studies have evaluated this relationship. One possible reason for the limited number of studies in this area may be the stringent inclusion criteria used in this review, which precisely required parameters with one point on the spine and one point on the pelvis, thereby focusing on a specific definition of spinopelvic alignment relevant to biomechanical considerations.”* |
| Since spinopelvic alignment can influence functional outcomes and has been linked to implant positioning—especially personalized cup alignment—it would be good to expand on that point. For example, did any of the included studies look at how spinopelvic mobility (e.g., stiff vs flexible pelvis) affected outcomes or guided component orientation? Consider briefly discussing whether any of the reviewed studies addressed these concepts, or mention this as an area for future research. | Thank you for highlighting the role of spinopelvic alignment in influencing functional outcomes and its link to implant positioning, particularly personalized cup alignment. While these are important factors, the objective of our review was specifically to evaluate the relationship between spinopelvic alignment, not mobility (e.g., stiff vs. flexible pelvis), and patient-reported outcome measures (PROMs). Assessing the relationship between spinopelvic mobility and functional outcomes or implant positioning was outside the current review's scope; therefore, none of the included studies looked at the relationship between spinopelvic mobility with functional outcomes or factors such as cup alignment or component orientation. Nonetheless, we have identified this as an important area for future research and have noted it in the manuscript with the following statement:  - Line 585-587: *“It would also be important for future reviews to examine the relationship between spinopelvic alignment and pelvic alignment including implant positioning or component orientation, and how these are associated with functional outcomes.”* |
| The conclusions accurately reflect the overall limitations in the literature. However, this paper can also serve as a useful roadmap for future work in this area. Consider adding a few specific recommendations, such as: The need for standardized outcome measures. More prospective studies using validated spinopelvic parameters. Better control for variables like lumbar spine pathology or pelvic stiffness. | Thank you for the feedback regarding the conclusion and its reflection on the limitations in literature. We have dedicated a paragraph within the discussion to explain the heterogeneity and the need for standardized outcome measures. As this point is thoroughly covered in the discussion section, we did not repeat it in the recommendations for future research to avoid redundancy. A minor edit was made to this paragraph for clarity.  We have revised the final sentence of the conclusion to emphasize the need for more prospective studies using validated spinopelvic parameters, as follows:  - Line 598 and 599: “*Additionally, more prospective studies are needed that investigate validated spinopelvic parameters and better control for lumbar spine pathology, such as LBP*.” |

| **Reviewers’ comments (reviewer 2)** | **Response to reviewers** |
| --- | --- |
| Reviewer #2: General comments:  The authors present a systematic review article on the relationship between patient-reported outcomes (PROMs) and spinopelvic alignment or physical functioning on total hip arthroplasty patients. Secondary objective was to study the relationship of physical measures with lower back pain. 51 studies in total were included to the review. The main finding was that there was no clear association between specific physical function measurements and PROMs but the quality of studies was low. Similarly, there were only 3 studies that reported lower back pain and no clear outcomes could be reported.  The study methods on gathering data are structured and scientifically sound. The study design was well constructed. The criticism and suggestions for improvements are mainly focused on the reporting of the data. While there is no word limit on the articles, the main text and the tables contain over 21000 words, which is way higher number than in most of the articles. Therefore, I recommend shortening the text and the tables, and possibly including rest of the data as a supplementary data. I also think that it is unnecessary to have multiple objectives on the review article (association of PROMs to three different outcomes). | We greatly appreciate your feedback on the study’s methodology and design. We have carefully considered your comments regarding the word count. To address this, we have provided table 2 on study characteristics and results as a supplementary file. Additionally, we have included supplementary material detailing the results of the eligibility confirmation process at the full-text review stage. These changes have significantly reduced the manuscript’s length: prior to revision, the word count from the introduction to the acknowledgements was 22,424 words; following these revisions, the total word count has been reduced to 7,135, including words in tables and in-text references.  This review was structured with two distinct objectives to address two separate and clinically relevant questions. While both involve physical measures, one focuses on their relationship with PROMs and the other on their relationship with low back pain (LBP). Given the inconsistent reporting in the literature and the limited ability of general PROMs to adequately capture the relationship between these physical measures and LBP, we considered a separate objective and analysis necessary to ensure clarity and precision in this context. |
| Specific comments:  Objectives  - Lines 61-2: “For THA, physical measures of spinopelvic alignment and physical function are important”. This line should be elsewhere, in the next paragraph where the authors describe the spinopelvic alignment. | Your specific feedback is much appreciated.  - Lines 61-2: This line has been moved at the beginning of the following paragraph, where the explanation of spinopelvic alignment is provided. |
| - Line 88-89: Is it necessary to report lower back pain as a separate secondary objective or could it be part of the other PROMs? | - Line 88-89: We appreciate your insightful comment. While LBP can be considered a component of PROMs, we chose to report it as a separate secondary objective due to its high prevalence and specific clinical relevance in the THA population. Additionally, our scoping search revealed that the relationship between physical measures of spinopelvic alignment and physical functioning with LBP has been infrequently and inconsistently reported in the literature. Given that general PROMs may not sufficiently capture the relationship between these physical measures and LBP, we believed it warranted focused analysis as a separate outcome to better understand its role in this context. |
| Materials and methods  - Table 1. The authors mention three outcome measures for inclusion to the review: 1) physical measures of spinopelvic alignment 2) physical measures of physical functioning and 3) PROMs. Later, they find that the studies for spinopelvic alignments and relationship to PROMs are scarce and the main focus stays on the physical functioning measurements. While this is a result as itself, would it be clearer if the article focused only to the physical functioning measurements? Alternatively, the title should be changed so that it emphasizes the physical functioning | We sincerely appreciate your insightful feedback. Our aim was to provide a comprehensive overview by including both physical functioning measures and spinopelvic alignment. While the narrative synthesis showed less heterogeneity in studies on physical functioning, this does not reflect a narrowed focus. Instead, the limited number and heterogeneity of spinopelvic studies made firm conclusions difficult, though they represent the full scope of available evidence. This highlights a critical gap and the need for further research in this area. Additionally, the research question was defined based on a preliminary scoping search, and the review was conducted in accordance with a predefined, published, and PROSPERO-registered protocol. As such, altering the study objectives at this stage could introduce bias and compromise the methodological integrity of the review. For further clarification, the following statement has been added to the synthesis paragraph in the results section:  - Line 320-324: *“Owing to substantial heterogeneity among studies on the association between spinopelvic alignment with PROMs such as variations in physical measures, PROMs, or follow-up timepoints, it was not possible to conduct a cross-sectional or longitudinal synthesis of the findings. Studies on LBP also could not be synthesized due to heterogeneity. Detailed results of individual studies are presented in S5 Appendix.”* |
| - Line 144: ChatGPT was used in the translation of the foreign language text. Although the translation probably works quite well, I’ m not sure if it has been validated as a proper translation of scientific texts. | Thank you for your thoughtful comment. Our decision to use ChatGPT was informed by the literature highlighting its superior ability to preserve stylistic features and complex linguistic structures compared to Google Translate, as well as its good performance in translating medical and technical literature. However, as noted in previous studies, this approach may have some limitations. To ensure accuracy, all translations in our study were reviewed by native speakers of the corresponding language. The following statement has been added to the manuscript:  *-* Line 154-158: *“We selected ChatGPT over Google Translate for its better preservation of stylistic elements, contextual nuances, and strong performance in medical translations[44]. However, as noted in previous studies, this approach may have some limitations [44, 45]. To ensure accuracy, all translations in our study were reviewed by native speakers of the respective languages.”* |
| - Lines 154 and 163: “a third reviewer”. You mean fourth? There was a mention of three authors who extracted data and that there was a separate mediator. | We are grateful for your careful review and valuable suggestion. We had two reviewers act as the second reviewer. One reviewer (SV) conducted the process for half of the studies with RR and the other half with LF—thus, each study was assessed by two reviewers. The third person (AR) served as a mediator and was available to resolve any disagreements that arose. We have clarified this in the revised manuscript as follows:  - Line 165 and 166: “*We had two reviewers act as the second reviewer. One reviewer (SV) conducted the process for half of the studies in collaboration with RR and the other half with LF.*” |
| - Line 176: reference to [49] Pais et al (2020). How is the text related to the reference? | Thank you for your detailed and helpful observations. In the study by Pais et al. (2020), the authors defined thresholds for rating the NIH Quality Assessment Tool as follows: 'good' for >80% of questions rated as 'yes,' 'fair' for 60–80%, and 'poor' for <60%. As we adopted an approach previously used by Pais et al. (2020), we cited their study accordingly.  We also added a reference to support the explanation of publication bias in the GRADE assessment for accuracy. |
| - Tables 3 and 4 are clear. | Thank you very much for your insightful feedback. |
| Results  - Lines 221-236. I think there is way too much text. I find it difficult to follow the narrative. A simple flow chart is enough. | We value your feedback and have addressed your points accordingly. For lines 221 to 232, a supplementary file was created to provide detailed information on eligibility confirmation, including full-text article retrieval, verification of THA indication, and requests for separate THA results. A correction was made in the retrieval summary, as one study had been counted twice in the full-text retrieval narrative. |
| - Table 2. This table has a summary of every article included to the review including name, publication year, study objectives, number of patients and sex, health conditions physical outcome measures, PROMs, follow-up time and summary of results. While its impressive, I find it very difficult to read, mainly due the length of up to 40 pages and the extensive use of abbreviations (footnotes are 15 lines long). Overall, its impossible to form a coherent summary of the results. I think it would be more beneficial if this table was transferred to supplementary data or to the end of the article and this table greatly shortened to include only key results of the each stydy. | We appreciate your thoughtful feedback on Table 2. Since this table contains all the important information on the studies, we have included it as a supplementary file to enhance the readability of the main text. |
| - Lines 209-310: Again, I don’t think it’ s necessary to report lower back pain separately. | Thank you for your feedback. Given the importance of LBP in this population, and the inconsistent findings in existing studies, we defined a secondary objective to provide an overview of the available evidence on the association between physical measures with LBP. This could help address current knowledge gaps and inform future research and clinical practice. |
| - Lines 403-418: The results here are confusing related to the methods of the article. In other studies which have been included to the review, the authors study the correlation between PROMs and actual measured physical functioning. If I have understood correctly, these measurements have been taken at the same time. However, the studies ion this paragraph ([82, 93, 10, 95]) focused on the preoperative measurements and how they predict postoperative outcomes, which in my opinion is a completely different kind of correlation. The authors could describe more clearly what kind of time-intervals were allowed when studying the correlation of PROMs and functional measurements or remove these studies. | We appreciate your precise and thorough review. As outlined in the eligibility criteria, we included all observational studies, retrospective and prospective longitudinal cohorts, as well as cross-sectional designs, that examined associations following THA, from pre- to post-surgery or cross-sectionally after surgery. These studies assessed associations in both cross-sectional and longitudinal formats, with no restrictions on the type of association (e.g., responsiveness, predictive, etc.). This approach was taken to provide a comprehensive overview of the relationships between the measures. The manuscript has been revised to clearly state, in Table 1 (Criteria for Inclusion and Exclusion of Studies), that no restrictions were applied regarding the type of association assessed. |
| - Lines 498-502: I think this paragraph is not needed. | While we understand the reviewer’s suggestion to remove the paragraph, we believe it is important to retain it, as patient partners played a meaningful role in the development of this study and contributed valuable feedback on the interpretation of the findings. To address the reviewer’s concern, we have revised the paragraph to provide a clearer explanation of their involvement and its relevance to the study as follows:  -Line 492-500: *“After presenting the findings to the patient partners, they rated the clarity as very to extremely clear, the alignment between key findings and conclusions as somewhat to extremely clear, and the potential impact as good to excellent, with one rating it as fair. Patients also highlighted the importance of pelvic alignment in understanding physical function and outcomes following THA. Additionally, some also noted hypermobility as a potential confounding factor to consider when interpreting the findings. While we could not incorporate their feedback directly, due to our focus on spinopelvic rather than pelvic parameters, and the heterogeneity that prevented synthesis to assess the effects of hypermobility, we have included these points as recommendations for future research.”*  We also have added their advice as the recommendation for future research in the related paragraph:  *-Line 587 and 588: “Furthermore, assessing the impact of hypermobility on these associations would be valuable.”* |
| Discussion  - Lines 515-516: In the methods it is stated that the objective was to study 1) spinopelvic alignment 2) physical functioning measures and 3) lower back pain and their relationship to PROMs. However, the spinopelvic alignment is barely discussed later on, possibly mainly because of the lack of studies. Again, my suggestion is to either remove the spinopelvic alignment altogether from the methods and focus on the physical functioning or add a proper paragraph to the discussion section. | Thank you for your helpful comment. Our objective was to evaluate the association between physical measures of spinopelvic alignment and physical functioning with PROMs following THA as well as to assess the relationship between these physical measures with LBP following THA. We acknowledge the reviewer’s suggestion and have added clarification in the relevant paragraph explaining why only a limited number of studies on spinopelvic parameters were included:  - Line 513-518: *“There was no synthesized evidence on the association between the physical measures of spinopelvic alignment with PROMs. Only six studies have evaluated this relationship. One possible reason for the limited number of studies in this area may be the stringent inclusion criteria used in this review, which precisely required parameters with one point on the spine and one point on the pelvis, thereby focusing on a specific definition of spinopelvic alignment relevant to biomechanical considerations.”* |
| - Lines 529-530: Again, see my comment on lines 403-418. | Thank you for your valuable feedback. Since we did not limit the type of association, we cited relevant reviews in the discussion, such as those addressing prognostic associations, to compare their findings with ours, highlight areas of agreement, and suggest possible explanations for any differences. We added the following statement to the discussion section, where we compared our findings with relevant reviews, including those on prognostic studies, as follows:  *-* Line 536-537: *“Additionally,* *the lack of consistent findings in our review may be influenced by statistical heterogeneity, as we included studies examining any type of association.”* |
