# Supplementary material for: Association between physical measures of spinopelvic alignment and physical functioning with patient reported outcome measures after total hip arthroplasty: Systematic review and narrative synthesis
Source: PLoS One. 2025 Dec 29;20(12):e0339615. doi: 10.1371/journal.pone.0339615 (PMC12747333; doi:10.1371/journal.pone.0339615)
Supplement: Attachment — Submitted filename: Response_to_reviewers_auresp_2.docx [file pone.0339615.s008.docx]

| **Reviewers’ comments (Editor Comments)** | **Response to reviewers** |
| --- | --- |
| Required: Revise the title to align with the actual evidence base, or clearly state early that evidence on spinopelvic alignment is very limited and that the main synthesis concerns physical functioning. | Thank you for your feedback. It is now clearly stated in the Abstract (page 2) that evidence on spinopelvic alignment is very limited, with the synthesis focusing predominantly on physical measures of physical functioning.  In Table 1 (page 7), under the inclusion criteria, a justification is provided for narrowing the focus to spinopelvic parameters defined by one point on the spine and one on the pelvis. It has also been explained in the Discussion section (page 33). |
| Acknowledge and clarify the inclusion of both prognostic and cross-sectional studies, and note the implications of this heterogeneity. | Thank you for your feedback. This has been addressed in the Design section (page 8) of the inclusion criteria (Table 1), where it is clarified that no restrictions were applied to the type of association, with examples provided in parentheses. Additionally, the following explanation has been added to the limitation section (page 36) to clarify that methodological heterogeneity may have contributed to the observed inconsistencies in the findings.  -Line 589-591: *“Since no limitations were placed on the type of association (e.g., concurrent, predictive, responsiveness, etc.), this methodological heterogeneity may contribute to the observed inconsistencies in the findings.”* |
| Recommended: Expand discussion of the evidence gap on spinopelvic alignment. | Thank you for your feedback. It has been clarified in the discussion (page 33) by revision of the explanation and adding the following explanation:  -Line 520-531: *“Only six studies have evaluated this relationship. However, in the scoping review by Pourahmadi et al. [20] on spinopelvic alignment and LBP following THA, more studies were identified. This discrepancy may reflect differences in how spinopelvic parameters were defined. In this review, spinopelvic alignment was narrowly defined as involving one parameter on the spine and one on the pelvis, which led to the exclusion of studies assessing pelvic parameters alone. The objectives also* *differed. Pourahmadi et al. [20] aimed to provide an overview of spinopelvic alignment and LBP following THA, whereas this review specifically focused on studies evaluating the association between spinopelvic alignment and LBP following THA. The limited number of studies addressing association further highlights a gap in the existing literature. This evidence gap limits our ability to draw firm conclusions about the influence of spinopelvic alignment on PROMs and underscores the need for standardized measurement approaches and systematic evaluation to enable more robust synthesis and clinical interpretation.”* |
| Conduct a final check for clarity and language. | We are grateful for your valuable suggestion. The files have been checked for clarity and language.  In the PRISMA diagram, the number of unretrieved full texts and the conference abstracts of the already included articles were reported together; for clarity, they are now presented separately, and the corresponding files were edited accordingly (Figure 1, S3 Appendix, manuscript).  One full text provided by the author, which had been inadvertently omitted, was added to the diagram in S3 Appendix.  Some minor edits were made to the manuscript, S3 Appendix, S4 Appendix, and S5 Appendix for improved clarity and consistency. |
| **Reviewers’ comments (Reviewer 1)** | **Response to reviewers** |
| The authors did a good job and met all of the reviewers' comments. They edited the manuscript and enhanced the scope of the review. I was delighted reading the current version of their manuscript. | Thank you for your feedback. We are glad to hear that the revisions meet your expectations. |
| **Reviewers’ comments (Reviewer 3)** | **Response to reviewers** |
| Reviewer #3: Major Comments and Suggestions:  Mismatch Between Title, Aims, and Actual Content:  Comment: The title and one of the primary objectives prominently feature "spinopelvic alignment." However, only 6 out of the 51 included studies specifically investigated this parameter under the strict inclusion criteria (requiring measurement points on both the spine and pelvis). This creates a significant discrepancy between the reader's expectation, set by the title, and the actual content of the review, which is predominantly a synthesis of evidence on broader "physical functioning" measures (e.g., strength, gait speed). Suggestions: Primary Suggestion: Revise the title. It is highly recommended to change the title to more accurately reflect the core content. Enhanced Clarification: If the current title is retained, a very clear and prominent statement must be added early in the Abstract and Introduction explicitly stating that the evidence regarding spinopelvic alignment is extremely limited based on the defined criteria, and that the main synthesis pertains to physical functioning measures. The explanations added in the introduction (Lines 73-77) and discussion (Lines 514-518) are necessary but may be insufficient to fully manage reader expectations set by the title. | Thank you for your feedback. We recognize that most of the included studies focused on the physical measures of physical functioning. We also acknowledge that we defined strict criteria for spinopelvic parameters, requiring inclusion of both spinal and pelvic regions to ensure a focused and consistent approach. The six studies included in the review represent all available evidence that met these criteria, highlighting a clear gap in existing literature.  Modifying the study title at this point would conflict with the established methodological framework and could affect the transparency of the review process as the research question was developed following a preliminary scoping search and implemented according to a predefined, published, and PROSPERO-registered protocol.  To provide further clarity, the following sentence has been added to the Abstract (page 2).  -Line 38-40: *“The evidence on spinopelvic alignment was extremely limited (6 studies), with the main synthesis focusing predominantly on physical measures of physical functioning.”*  The following justification for including only parameters with one point on the spine and one point on the pelvis has also been provided in the inclusion criteria (page 7) in Table 1.  -Page 7- Table 1: *“To accurately characterize spinopelvic alignment, parameters defined by one point on the spine and one on the pelvis were included. This approach ensures that the selected measures specifically reflect the interaction between spinal and pelvic regions, rather than isolated regional parameters.”*  Given the expectations set by the title and the limited evidence on the association between spinopelvic alignment and PROMs, the Discussion (page 33) and conclusion section (page 37) have been expanded accordingly.  -Line 520-531: *“Only six studies have evaluated this relationship. However, in the scoping review by Pourahmadi et al. [20] on spinopelvic alignment and LBP following THA, more studies were identified. This discrepancy may reflect differences in how spinopelvic parameters were defined. In this review, spinopelvic alignment was narrowly defined as involving one parameter on the spine and one on the pelvis, which led to the exclusion of studies assessing pelvic parameters alone. The objectives also* *differed. Pourahmadi et al. [20] aimed to provide an overview of spinopelvic alignment and LBP following THA, whereas this review specifically focused on studies evaluating the association between spinopelvic alignment and LBP following THA. The limited number of studies addressing association further highlights a gap in the existing literature. This evidence gap limits our ability to draw firm conclusions about the influence of spinopelvic alignment on PROMs and underscores the need for standardized measurement approaches and systematic evaluation to enable more robust synthesis and clinical interpretation.”*  -Line 609-612: *“A key finding of this review is that the limited and heterogeneous nature of available studies on the association between spinopelvic alignment and PROMs prevented meaningful synthesis, highlighting a clear gap in existing literature.”* |
| Clarity on the Type of "Association" Assessed:  Comment: The manuscript combines results from prognostic studies (e.g., using preoperative measures to predict postoperative outcomes) and cross-sectional studies (assessing correlations at a single time point, e.g., postoperatively). These study designs address different scientific questions (prediction vs. concurrent relationship). Combining them in a narrative synthesis may contribute to heterogeneity and obscure clear interpretations. Suggestion: It is important to explicitly address this in the Methods (e.g., in inclusion criteria) or Results section. A brief statement should clarify that the review included studies examining any temporal association (predictive, concurrent, etc.), and acknowledge that this methodological heterogeneity might be a factor in the inconsistent findings. When presenting results, consider grouping or clearly labeling studies based on the type of association they assessed to aid interpretation. | We appreciate your thorough review. As indicated in the inclusion criteria (Table 1), no restrictions were applied to the type of association. To provide further clarity, the term *“concurrent”* has been added to the list of examples in parentheses within the design section (page 8) of the inclusion criteria (Table 1). Additionally, it has been clarified in the limitation section (page 36) that methodological heterogeneity may have contributed to the observed inconsistencies in the findings.  -Line 589-591: *“Since no limitations were placed on the type of association (e.g., concurrent, predictive, responsiveness, etc.), this methodological heterogeneity may contribute to the observed inconsistencies in the findings.”*  Regarding the synthesis, it has been divided into two parts, with the interpretation separated based on study design. The sections are titled: “Associations between measures in cross-sectional studies” and “Associations between measures in cohort longitudinal studies”. |
| Strengthening the Discussion on Evidence Gaps:  Comment: The discussion correctly notes the scarcity of evidence on spinopelvic alignment. However, this "evidence gap" itself should be framed as a key finding of the review. | Your feedback is greatly appreciated. To better reflect this in the study, we have added to the Conclusion (page 37) that the limited studies on the association between spinopelvic alignment and PROMs prevented meaningful synthesis, underscoring a clear gap in the literature.  -Line 609-612: *“A key finding of this review is that the limited and heterogeneous nature of available studies on the association between spinopelvic alignment and PROMs prevented meaningful synthesis, highlighting a clear gap in existing literature.”* |
